# Chemoenzymatic tandem cyclization for the facile synthesis of bicyclic peptides

**DOI:** 10.1038/s42004-024-01147-w

**Published:** 2024-03-28

**Authors:** Masakazu Kobayashi, Naho Onozawa, Kenichi Matsuda, Toshiyuki Wakimoto

**Affiliations:** Faculty of Pharmaceutical Sciences, Hokkaido University, Kita 12, Nishi 6, Kita-ku, Sapporo, 060-0812 Japan

**Keywords:** Biocatalysis, Peptides

## Abstract

Bicyclic peptides exhibit improved metabolic stabilities and target specificities when compared to their linear or mono-cyclic counterparts; however, efficient and straightforward synthesis remains challenging due to their intricate architectures. Here, we present a highly selective and operationally simple one-pot chemoenzymatic tandem cyclization approach to synthesize bicyclic peptides with small to medium ring sizes. Penicillin-binding protein-type thioesterases (PBP-type TEs) efficiently cyclized azide/alkyne-containing peptides in a head-to-tail manner. Successive copper (I)-catalyzed azide-alkyne cycloaddition generated bicyclic peptides in one-pot, thus omitting the purification of monocyclic intermediates. This chemoenzymatic strategy enabled the facile synthesis of bicyclic peptides bearing hexa-, octa-, and undecapeptidyl head-to-tail cyclic scaffolds.

## Abstract (web summary)

Bicyclic peptides exhibit improved metabolic stabilities, membrane permeabilities, and target specificities over their linear and mono-cyclic counterparts, however, efficient bicyclization remains challenging. Here, the authors develop a one-pot tandem chemoenzymatic bicyclization by combination of penicillin-binding protein-type thioesterase-mediated head-to-tail macrolactamization and copper(I)-catalyzed azide–alkyne cycloaddition.

## Introduction

Macrocyclization of a peptide backbone reduces its conformational flexibility, thus potentially improving the metabolic stability, membrane permeability, and target specificity^1–3^. Although numerous chemical methodologies have been invented, macrolactamization has remained challenging as it inherently competes with the oligomerizing side reaction^4^. Enzyme utilization is a promising alternative to the chemical methodologies, as enzymes efficiently catalyze chemo- and regiospecific cyclization under mild conditions^5^. However, extensively studied biocatalysts derived from ribosomal pathways such as sortase A^6,7^, trypsin^8^, asparaginyl endopeptidases like butelase-I^9^ and OaAEP1b^10^, and subtilisin variants like Omniligase-I and others^11–13^ are generally limited to cyclizing peptides longer than ten amino acid residues, and thus smaller peptides, which potentially exhibit better bioavailability^14^ and less immunogenicity^15^, are beyond the scope of these biocatalysts. There are several enzyme families that can cyclize substantially shorter peptides. These include the subtilisin-like proteases in cyanobactin biosynthesis like PatG^16^, and the type-I thioesterase domains in non-ribosomal peptide biosynthetic pathways like TycC-TE^17^. Their distinctive properties make them intriguing biocatalysts; however, they suffer from narrow substrate scopes^16,18^, requirements for peculiar recognition sequences^19^, or low catalytic efficiencies^20^.

Penicillin-binding protein-type thioesterases (PBP-type TEs) have emerged as a promising family of peptide cyclases^21–30^. PBP-type TEs efficiently cyclize peptides, preferentially those with fewer than ten residues, although they also tolerate substantially larger substrates^28^. PBP-type TEs catalyze head-to-tail cyclization in the biosynthesis of cyclic non-ribosomal peptides (cyclic NRPs). Since the identification of the first PBP-type TE, SurE^21^, several homologous enzymes such as PenA^27^, WolJ^28,31^, and Ulm16^29^ with alternative substrate scopes have been identified from actinobacteria. Although PBP-type TEs require non-peptidyl leaving groups, which must be installed at the *C*-terminus of linear substrates, we have established a solid phase peptide synthesis (SPPS)-based method that enables rapid access to the substrate peptides with a *C*-terminal ethylene glycol (EG) leaving group^28^. The method yields the EG-embedded peptides with sufficient purities to be readily cyclized by PBP-type TEs after cleavage from the solid phase, thus circumventing the laborious purification of intermediates and streamlining the procedures^28^. Notably, the enzymatic cyclization generally exhibits high reaction efficiency and yields pure cyclic products, thereby opening the possibility for direct post-cyclization modification.

**Fig. 1:**
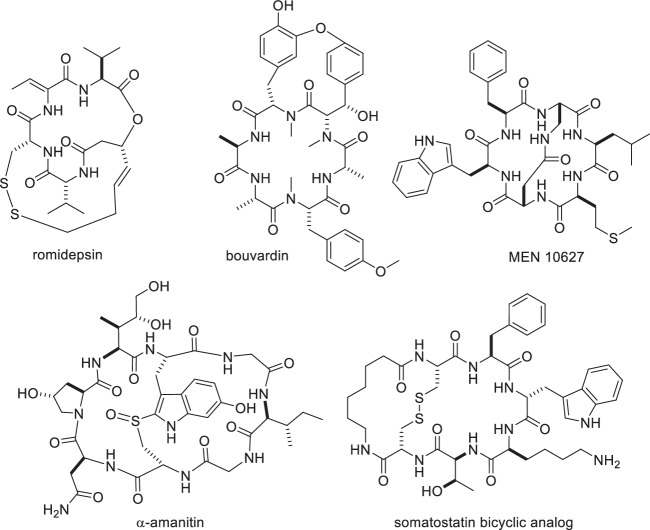
Bicyclic peptides. Representative natural and synthetic bicyclic peptides are shown.

Several natural and synthetic bioactive cyclic peptides possess additional linkages within their macrocyclic scaffolds^32,33^. The linkages confer improved metabolic stability and target specificity due to the enhanced conformational rigidity, compared to their linear or monocyclic counterparts^34–36^. Due to their enormous potential as chemical probes and therapeutics, numerous chemical methodologies for synthesizing and screening bicyclic or multicyclic peptides have been developed^35–38^, such as those involving thioether formation^33^. Enzymes have occasionally been employed in the synthesis of multicyclic peptides^9,39–42^, however, smaller ring scaffolds has remained beyond the scope of enzymatic methodologies despite of their remarkable potential as therapeutics that has been demonstrated through several natural and synthetic precedents^43–47^ (Fig. 1).

**Fig. 2:**
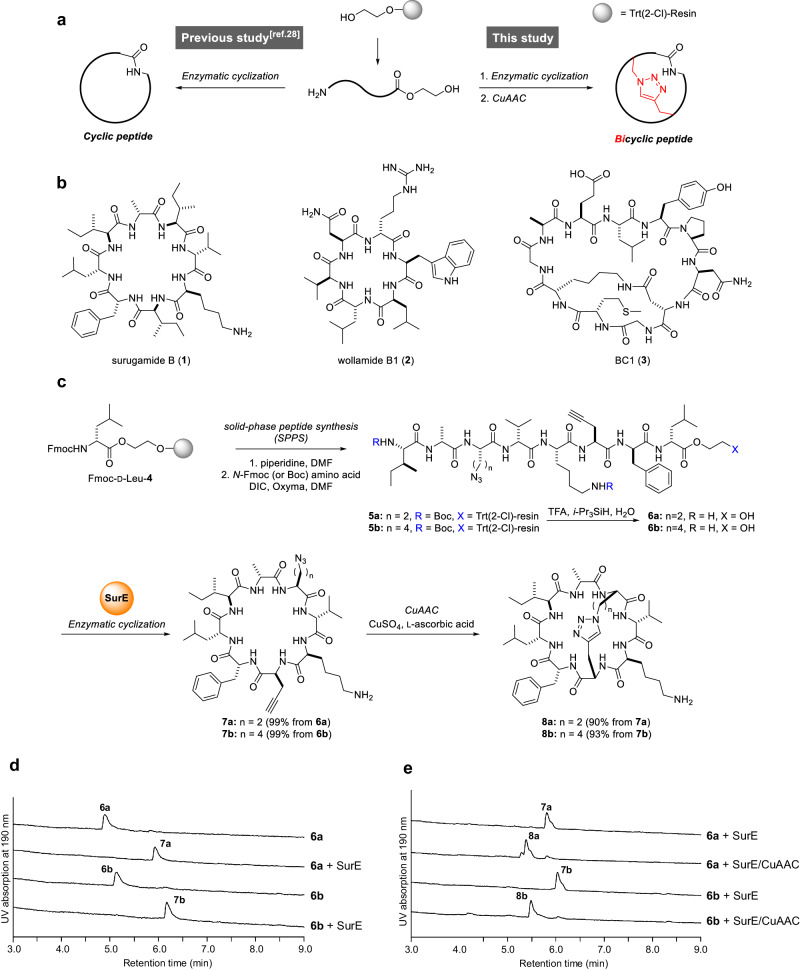
Chemoenzymatic synthesis of bicyclic peptides. **a** Schematic representation of the previous^28^ and present work. **b** Cyclic peptides used as models for chemoenzymatic bicyclization. **c** Synthetic scheme for the bicyclic analogs of surugamide B (**8a**, **8b**). Yields were determined by HPLC. **d** HPLC analysis of SurE-mediated cyclization using **6a** and **6b** as substrates. **e** HPLC analysis of reaction mixtures after CuAAC. Enzymatic reaction mixtures were directly subjected to CuAAC.

Herein we report a chemoenzymatic approach to synthesizing bicyclic scaffolds based on small to medium head-to-tail cyclic peptides. We sequentially performed the PBP-type TE-mediated cyclization and the copper(I)-catalyzed azide-alkyne cycloaddition (CuAAC)^48,49^ in a one-pot manner (Fig. 2a). This tandem cyclization approach facilitated rapid access to the constrained bicyclic scaffolds based on small- to medium-sized head-to-tail cyclic peptides.

## Results and discussion

First, we assessed the compatibility of the enzymatic cyclization with peptide substrates bearing clickable functionalities (i.e., azide and alkyne groups) (Fig. 2c). To this end, we substituted L-Ile3 and L-Ile6 of the *seco*-surugamide B sequence with an azide-containing residue L-Dab(N_3_) and an alkyne-containing residue L-propargylglycine (L-Pra), respectively, to give **6a**. Variant **6b**, bearing L-Lys(N_3_) instead of L-Dab(N_3_), was also designed to investigate the impact of the length of the azide-containing side-chain upon CuAAC. The conventional DIC/Oxyma-mediated Fmoc-based SPPS was performed using the EG-preloaded solid support **4** to afford **5a**/**5b**. Peptides were cleaved and deprotected by treatment with TFA/*i*-Pr_3_SiH/H_2_O ( = 95:2.5:2.5) to give the ethylene glycol (EG)-embedded peptides **6a**/**6b**. Next, 5 mol% of SurE was mixed with **6a**/**6b** and incubated at 30 °C for 2 h. This resulted in the quantitative conversion of **6a**/**6b** to their monocyclic counterparts **7a**/**7b**, with no detectable accumulation of hydrolyzed products (Fig. 2d), showing the perfect compatibility of SurE with clickable functionalities. The enzymatic reaction mixtures were directly subjected to CuAAC by adding CuSO_4_ and L-ascorbic acid and then incubated for twelve hours at room temperature. The LC-MS analysis showed the efficient conversion of **7a**/**7b** to the new compounds **8a**/**8b**, with identical *m/z* values but distinct column retention times (Fig. 2e). MS/MS analysis of **8a**/**8b** showed the disappearance of the fragment ions containing azide/alkyne groups and the generation of fragment ions consistent with the substructures containing a 1,4-disubstituted 1,2,3-triazole moiety (Fig. S1), indicating the successful intramolecular CuAAC. The structures of **8a**/**8b** were finally confirmed by comparison with the authentic standards, which were synthesized via chemical head-to-tail cyclization followed by CuAAC (Fig. S2). Taken together, the bicyclic analogs of surugamide B **8a**/**8b** were successfully synthesized in a one-pot manner from the linear peptides by sequentially performing two site-selective cyclizations: the PBP-type TE-mediated cyclization followed by CuAAC.

**Fig. 3:**
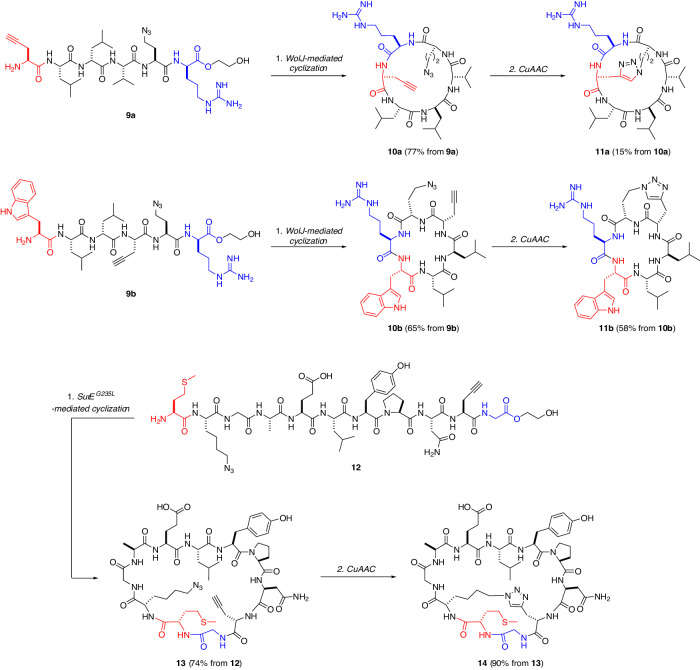
Chemoenzymatic tandem cyclization of hexa- and undecapeptides. Yields were determined by HPLC analysis (Figs. S3 and S7). *N*- and *C*-terminal residues in the enzymatic cyclization are colored red and blue, respectively.

We next attempted to apply this method to peptides with different ring sizes (Fig. 3). As a model for bicyclization, we chose the cyclic hexapeptide wollamide B1 (Fig. 2b), a synthetic analog of the naturally occurring cyclic peptide wollamide B, with improved antituberculosis activity^50^. Previous work demonstrated that WolJ, another member of the PBP-type TEs, efficiently cyclizes the wollamide B1 sequence^28^, making it a feasible target for benchmarking the present synthetic method. An analog of *seco*-wollamide B1 (**9a**), which possesses L-Pra in the 1st position and L-Dab(N_3_) at the 5th position, was synthesized from Fmoc-D-Arg(Pbf)-**4**. To determine the effect of the distance between the alkyne/azide residues in intramolecular CuAAC, we also synthesized the *seco*-wollamide B1 analog (**9b**) with L-Pra in the 4th position and L-Dab(N_3_) at the 5th position. In the in vitro enzymatic reactions, WolJ successfully cyclized both **9a** and **9b** and gave their monocyclic counterparts **10a** and **10b**, respectively, showing its broad substrate tolerance (Fig. S3). As a result of successive CuAAC, both **10a** and **10b** were converted to the bicyclic scaffolds **11a** and **11b**. The conversions were significantly more efficient for **11b**: **10a** to **11a** in 15% whereas **10b** to **11b** in 58%. The decreased conversion for **10a** could be rationalized by the conformational rigidity of the hexapeptide: When using two residues in the (*i*, *i* + 2) positions as in **10a**, the rigid peptide backbone would prevent two clickable moieties from being in proximal space. A larger and flexible cyclic scaffold would be more amenable for CuAAC, as in the case of **8a**/**8b**, in which the click residues are separated by two residues, yet the intramolecular CuAAC proceeded efficiently (Fig. 2d, e). Overall, although we observed certain limitations on the bridging position, these results showed that the tandem cyclization method is compatible with the hexapeptidyl bicyclic scaffold.

We next investigated the impact of reversing the cyclization processes (i.e., CuAAC followed by enzymatic reactions). To this end, octapeptide **6b** and hexapeptides **9a**/**9b** were subjected to CuAAC to give triazole-containing monocyclic peptides, **7b’,** **10a’**, and **10b’**, respectively. When subjected to enzymatic reactions, distinct outcomes were observed. The triazole-octapeptide **7b’** remained intact after the incubation with SurE (Fig. S4), showing that it is unable to form peptide-*O*-enzyme complex. In the case of the triazole-hexapeptide **10a’**, it underwent quantitative hydrolysis of the EG leaving group by WolJ (Fig. S5). The pre-cyclized peptides **7b’** and **10a’** were disfavored probably due to their reduced flexibilities, preventing them from adopting conformations suitable for enzyme recognition. On the other hand, the hexapeptide **10b’** with cross-link between adjacent residues was cyclized to the bicyclic peptide **11b** in 43% conversion (Fig. S6), suggesting that the adjacent cross-linking had less impact on enzyme recognition. Although the conversion remains low compared to the linear substrate **9b** (65% conversion), this is the first demonstration of PBP-type TE-mediated cyclization on a pre-cyclized substrate. Overall, these investigations indicated that constructing bicyclic scaffolds is more feasible when enzymatic cyclization is performed first, followed by CuAAC, rather than in the reverse order.

Having established the feasibility of performing chemoenzymatic tandem cyclization with natural product-based peptide substrates originally associated with PBP-type TEs, the next goal was to synthesize a variant of a biologically active bicyclic peptide that originated from random discovery methods, such as phage display. To this end, we selected BC1 (**3**) as the target for synthesis (Fig. 2b). BC1 is an undecapeptidyl head-to-tail cyclic peptide bridged by an amide linkage between L-lysine and L-aspartate. BC1 selectively binds to the Src homology 2 (SH2) domain of growth-factor-bound protein 2 (Grb2), a well-established target for breast and other cancers^34^. To synthesize BC1 analogs, namely BC1-triazole (**14**), via the tandem cyclization method, the following points were considered: (i) The amide bridge was substituted with its bioisostere 1,4-disubstituted 1,2,3-triazole to enable the intramolecular bridging via CuAAC at the final step of synthesis. (ii) BC1 solely consists of L-amino acids and glycines, thus conflicting with the diligent requirement of the native SurE-mediated cyclization for the D-amino acid at the *C*-terminus of the substrate^21^. In previous work, we manipulated the specificity of SurE by mutating a single residue in the substrate binding pocket and generated the SurE^G235L^ variant that efficiently cyclizes peptides with glycine at their *C*-termini^28^. Accordingly, we set the enzymatic ring-closing site between glycine and L-methionine and designed an undecapeptidyl linear precursor (**12**), and employed the SurE^G235L^ variant for cyclization (Fig. 3). Compound **12** was synthesized from Fmoc-Gly-**4** with 86% efficiency in 21 steps. The SurE^G235L^ variant efficiently cyclized **12** to give **13** (74% conversion) (Fig. S7). Successive CuAAC afforded **14** in one-pot (90% conversion from **13**) (Fig. 3 and S7). A variant **12’** with swapped alkyne/azide positions was converted to bicyclic peptide **14’** with decreased conversion, which may be attributed to a conformational difference affecting the second CuAAC (Fig. S8).

Overall, PBP-type TE-mediated cyclization followed by CuAAC generated bicyclic scaffolds based on head-to-tail cyclic peptides with six, eight, and eleven amino acid residues. The results demonstrated that the appropriate choices of cyclases and cyclization positions enable facile access to bicyclic peptides, including BC1 analogs, which are unrelated to PBP-type TEs in terms of their origins. The presented chemoenzymatic tandem cyclization approach will potentially accelerate the construction of compound libraries with highly constrained bicyclic peptides based on smaller ring sizes. The full compatibility between PBP-type TE-mediated cyclization and CuAAC provides valuable opportunities for post-cyclization modifications, which are not limited to bicyclization but also include intermolecular coupling with diverse molecules such as cyclic peptides, glycosides, or biologics.

## Methods

### General remarks

^1^H and ^13^C NMR spectra were recorded on a JEOL ECZ500 (500 MHz for ^1^H NMR) or a JEOL ECZ400 (400 MHz for ^1^H NMR) spectrometers. Chemical shifts are denoted in δ (ppm) relative to residual solvent peaks as internal standard (DMSO-*d*_6_, ^1^H δ 2.50, ^13^C δ 39.5). ESI-MS spectra were measured by a Thermo Scientific Exactive mass spectrometer or a SHIMADZU LCMS-2050 spectrometer. Optical rotations were recorded on a JASCO P-1030 polarimeter. High-performance liquid chromatography (HPLC) experiments were performed with a SHIMADZU HPLC system equipped with a LC-20AD intelligent pump. MS/MS analysis was performed with amaZon SL-NPC (Bruker Daltonics) using helium gas with an amplitude value 1.0 V. The chemical structures of enzyme substrates and enzymatic reaction products are analyzed by MS/MS (Supplementary data 1) and/or NMR (Supplementary data 2). All reagents were used as supplied unless otherwise noted.

### Preparation of the peptide cyclases

Expression plasmids for SurE, WolJ, and SurE^G235L^, were constructed previously (Table S1)^21,28^. 50 μg/ml kanamycin was used for the selection of *E. coli* host harboring pET28a-based SurE and SurE^G235L^ expression plasmids, whereas 200 μg/ml ampicillin was used for the selection of *E. coli* host harboring pColdII-based WolJ expression plasmids. A single colony of *E. coli* host was inoculated into 10 mL of 2xYT media (1.6% Bacto tryptone, 1.0% Bact yeast extract, 0.5% NaCl) containing an appropriate antibiotic and was cultured at 37 °C overnight as seed culture. 2.0 mL of cultural broth was transferred to 200 mL of 2xYT media containing an appropriate antibiotic and cultured at 37 °C for three hours. The broth was cooled on ice and 0.1 mM of isopropyl-β-D-thiogalactopyranoside (IPTG) was added to induce the expression of recombinant enzymes. *E. coli* was cultured at 16 °C for overnight. Cells were harvested by centrifugation with 3500 × *g* for 10 min at 4 °C and washed with wash buffer (20 mM Tris-HCl pH 8.0, 150 mM NaCl). Cells were resuspended into lysis buffer (20 mM Tris-HCl pH 8.0, 150 mM NaCl, 20 mM imidazole pH 8.0), then successfully homogenized by sonication. Cell debris was precipitated by centrifugation with 20,630 × *g* for 20 min at 4 °C, then the supernatant was subjected to Ni-NTA His-Bind® resin (Merck Millipore), which was equilibrated by lysis buffer (20 mM Tris-HCl pH 8.0, 150 mM NaCl, 20 mM imidazole pH 8.0). The column was washed with additional lysis buffer, then eluted with elution buffer (20 mM Tris-HCl pH 8.0, 150 mM NaCl, 500 mM imidazole pH 8.0). Imidazole was removed by an Amicon Ultra 0.5 ml filter (Merck Millipore). The concentrations of proteins were measured using a Bio-Rad protein assay kit (Bio-Rad).

### Chemical synthesis

The details of the chemical synthesis of compounds (Table S2) are described in the Supplementary methods.

### Enzymatic peptide cyclization

Enzymatic cyclization was initiated by adding 20 μM of enzymes (i.e., SurE, WolJ, or SurE^G235L^ mutant) to 100 μL of reaction mixtures containing 20 mM Tris-HCl (pH 8.0) and 400 μM substrate peptides. Reaction mixtures were incubated at 30 °C for 3 h for cyclizing **6a**/**6b**. Reaction mixtures were incubated at 30 °C overnight for cyclizing other substrates. Reactions were directly subjected to CuAAC as described below.

### Copper(I)-catalyzed cycloaddition (CuAAC)

CuAAC was performed by directly adding 2 mM of CuSO_4_⋅5H_2_O and 2 mM of L-ascorbic acid into the enzymatic reaction mixtures. Mixtures were incubated at 30 °C overnight, and then subjected to downstream analysis.

### HPLC analysis of enzymatic reaction

Samples were diluted 10-fold with methanol, then centrifuged at 20,630 × *g* for 10 min. Resultant supernatants were subjected to the Shimadzu HPLC system coupled with mass spectroscopy (LCMS-2050) operated in positive mode. Samples were separated by a reversed-phase column COSMOSIL 5C_18_-MS-II 2.0 × 150 mm column (nacalai tesque). H_2_O + 0.1% formic acid and acetonitrile + 0.1% formic acid were used as mobile phases A and B, respectively. Samples were eluted with a gradient mode of 10 to 90% for mobile phase B over 10 min with a flow rate of 0.4 ml/min.

### Reporting summary

Further information on research design is available in the Nature Portfolio Reporting Summary linked to this article.

### Supplementary information

Supplementary Information

Description of Additional Supplementary Files

Supplementary Data 1

Supplementary Data 2

Reporting Summary

## Data Availability

Supplementary Information includes supplementary methods and supplementary results. Supplementary Data 1 file contains MS2 spectra. Supplementary Data 2 file contains NMR spectra.
