# Supplementary material for: Chemoenzymatic tandem cyclization for the facile synthesis of bicyclic peptides
Source: Commun Chem. 2024 Mar 28;7:67. doi: 10.1038/s42004-024-01147-w (PMC10978974; doi:10.1038/s42004-024-01147-w)
Supplement: Supplementary file 1 — Supplementary Information [file 42004_2024_1147_MOESM1_ESM.pdf]

Supplementary information

Chemoenzymatic tandem cyclization for the facile synthesis of bicyclic peptides

Masakazu Kobayashi, Naho Onozawa, Kenichi Matsuda*, Toshiyuki Wakimoto*

Faculty of Pharmaceutical Sciences, Hokkaido University, Kita 12, Nishi 6, Kita-ku, Sapporo 060-0812, Japan

Table of contents

Supplementary methods

Chemical synthesis

Table S1. Enzymes used in this study

Table S2. Compounds list

Figure S1. MS/MS spectra of monocyclic peptides (**7a**/**7b**), and bicyclic peptides (**8a**/**8b**)

Figure S2. HPLC comparison of chemoenzymatically synthesized **8a**/**8b** with synthetic standards

Figure S3. Chemoenzymatic tandem cyclization of wollamide sequences

Figure S4. SurE-reaction on CuAAC-cyclized peptide **7b'**

Figure S5. WolJ-reaction on CuAAC-cyclized peptide **10a'**

Figure S6. WolJ-reaction on CuAAC-cyclized peptide **10b'**

Figure S7. Chemoenzymatic tandem cyclization of BC1 sequences **12**

Figure S8. Chemoenzymatic tandem cyclization of BC1 sequences **12'**

Supplementary method

Chemical synthesis

Procedure for solid-phase peptide synthesis (SPPS)

Step 1: Fmoc group of the solid supported peptide was removed by using 20% piperidine/DMF solution. Reaction vessel was shaken for 5 min at room temperature.

Step 2: The resin in the reaction vessel was washed with DMF (×3) and CH_2Cl_2 (×3).

Step 3: To the solution of Fmoc amino acid (4 eq) were added *N,N′*-diisopropylcarbodiimide (DIC, 4 eq, 0.50 M in DMF) and Oxyma (4 eq, 0.50 M in DMF). After 2-3 min of pre-activation, the mixture was injected to the reaction vessel. The resulting mixture was shaken for 30 min at 37°C

Step 4: The resin in the reaction vessel was washed with DMF (×3) and CH_2Cl_2 (×3).

Fmoc Amino acids were condensed onto the solid support by repeating Step 1-4.

Clickable building blocks used in this study

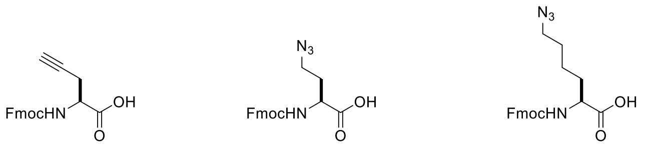

Fmoc-L-propargylglycine (Fmoc-L-Pra-OH) | 4-azido-*N*-Fmoc-L-homoalanine (Fmoc-L-Dab(N_3)-OH) | 6-azido-*N*-Fmoc-L-norleucine (Fmoc-L-Lys(N_3)-OH)

Synthesis of *seco*-surugamide B-EG analogs 6a and 6b

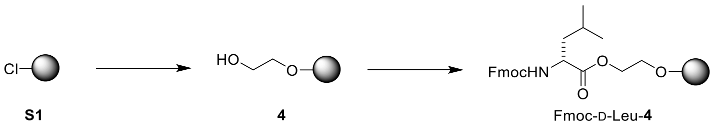

Fmoc-D-Leu-4: 2-chlorotrityl chloride resin **S1** (132 mg, 0.178 mmol) in Libra tube was swelled with CH_2Cl_2 for 10 min, and then excess solvent was removed by filtration. To the resin was added a solution of ethylene glycol (EG) (22.1 mg, 0.356 mmol), and *i*-Pr_2NEt (46.0 mg, 0.356 mmol) in CH_2Cl_2 (2.0 mL) and shaken for 2 h at 37 °C to give EG-2-chlorotrityl resin **4**. To the resin was added a solution of Fmoc-D-Leu-OH (126 mg, 0.356 mmol), DIC (112 μL, 0.713 mmol) and 4-dimethylaminopyridine (DMAP, 2.17 mg, 0.0178 mmol) in CH_2Cl_2 (2.00 mL) and stirred for 3 h at 37 °C to give Fmoc-D-Leu-**4**. Dried Fmoc-D-Leu-**4** was added with 20% piperidine in DMF and stirred

for 1 h. The supernatant was diluted with DMF and was subjected to UV measurement. Loading rate for Fmoc-D-Leu-**4** was calculated to be 0.459 mmolg^{-1} from observed UV absorbance at 301 nm.

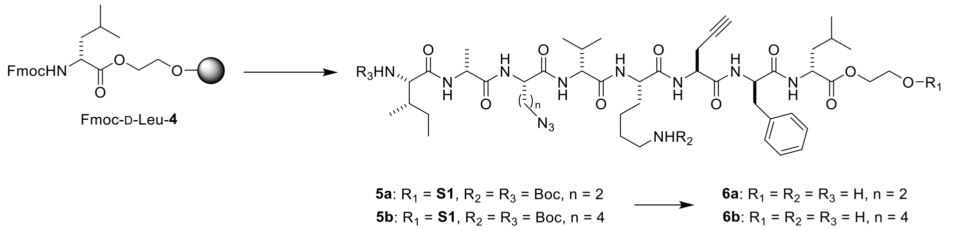

***seco*-surugamide B-EG analogs (6a and 6b)**: Fmoc-D-Leu-**4** (0.0250 mmol) in Libra tube was swelled in CH_2Cl_2 for 10 min, which was subjected to 7 cycles [Fmoc-D-Phe-OH, Fmoc-L-Pra-OH, Fmoc-L-Lys(Boc)-OH, Fmoc-D-Val-OH, Fmoc-L-Dab(N_3)-OH, Fmoc-D-Ala-OH, Boc-L-Ile-OH] of SPPS protocol to afford resin bound peptides **5a**. To afford **5b**, Fmoc-L-Lys(N_3)-OH was used instead of Fmoc-L-Dab(N_3)-OH. To the peptide **5a/5b** were added TFA/H_2O/*i*-Pr_3SiH = 95:2.5:2.5 (1.0 mL), being shaken for 30 min, and then the reaction mixture was filtered. The filtrate was diluted with Et_2O (25 mL) and was chilled (−80 °C), then centrifuged with 3,500 × *g* for 10 min at 4 °C to afford crude **6a** and **6b**, respectively. The crude material was purified by HPLC with COSMOSIL 5C$_{18}$-MS-II 20 mm I.D×250 mm, which was eluted by mobile phase MeCN:H_2O (= 35:65) containing 0.05% TFA with flow rate at 10 ml/min to afford **6a** (21.0 mg, 88.0% for 15 steps). For the purification of **6b**, the same column was eluted by MeCN:H_2O (= 40:60) containing 0.05% TFA with flow rate at 10 ml/min to afford **6b** (15.8 mg, 68.9% for 15 steps). Both compounds were obtained as a colorless amorphous solid.

6a: $[\alpha]_D^{21}$ +6.35 (*c* 2.02, DMSO); ^{1}H NMR (400 MHz, DMSO-*d*$_6$) δ 8.68 (d, *J* = 7.2 Hz, 1H), 8.41 (d, *J* = 7.2 Hz, 1H), 8.26 (q, *J* = 8.4 Hz, 2H), 8.12 (d, *J* = 14.8 Hz, 5H), 7.81 (s, 2H), 7.19-7.09 (m, 5H), 4.55 (s, 2H), 4.45 (t, *J* = 7.0 Hz, 2H), 4.32-4.25 (m, 7H), 4.01 (s, 3H), 3.66 (s, 1H), 3.53-3.46 (m, 2H), 3.25 (q, *J* = 7.2 Hz, 2H), 3.04-2.95 (m, 1H), 2.74-2.61 (m, 3H), 2.23 (m, 1H), 2.12 (m, 1H), 1.89 (m, 2H), 1.72 (m, 2H), 1.59-1.45 (m, 7H), 1.18 (d, *J* = 6.8 Hz, 3H), 1.04 (m, 1H), 0.87-0.75 (m, 18H); ^{13}C NMR (100 MHz, DMSO-*d*$_6$) δ 172.9, 172.1, 172.0, 171.7, 171.4, 171.1, 170.0, 168.0, 138.1, 129.8 (2 ×CH), 128.5 (2×CH), 126.8, 80.8, 73.4, 66.8, 59.4, 57.6, 56.7, 53.9, 52.4, 51.9, 51.0, 50.3, 50.2, 48.8, 48.1, 38.2, 36.6, 32.5, 32.0, 31.7, 27.0, 24.7, 24.5, 23.3, 22.7, 22.6, 22.4, 21.9, 19.7, 19.6, 18.2, 14.9, 11.5; HRMS (ESI) calcd for $C_{46}H_{75}O_{10}N_{12}^+$ $[M+H]^+$ 955.57236, found 955.57085

6b: $[\alpha]_D^{21}$ +4.40 (*c* 1.45, DMSO); ^{1}H NMR (400 MHz, DMSO-*d*$_6$) δ 8.65 (d, *J* = 7.6 Hz, 1H), 8.43 (d, *J* = 7.2 Hz, 1H), 8.25 (d, *J* = 8.0 Hz, 2H), 8.17 (d, *J* = 5.6 Hz, 3H), 8.10 (s, 2H), 7.78 (s, 2H), 7.23 (s, 5H), 4.59-4.54 (m, 3H), 4.36-4.27 (m, 4H), 4.05 (s, 2H), 3.69 (m, 2H), 3.32-3.27 (m, 2H), 3.06 (m, 1H), 2.78-2.65 (m, 4H), 2.30-2.14 (m, 2H), 1.94 (m, 1H), 1.76 (m, 1H), 1.63-1.48 (m, 13H), 1.33-1.20

(m, 7H), 1.13-1.05 (m, 2H), 0.91-0.80 (m, 18H); ^{13}C NMR (100 MHz, DMSO-d_6) δ 172.4, 171.6, 171.6,171.4, 171.2, 170.8, 169.5, 167.3, 137.6, 129.3 (2×CH), 128.0 (2×CH), 126.3, 80.4, 73.0, 66.3, 58.9, 57.0, 56.2, 53.4, 51.9, 51.6, 51.5, 50.6, 50.6 (2×CH), 48.1, 37.8, 36.2, 32.4, 31.6, 31.3, 27.7, 26.6, 24.2, 24.0, 22.8, 22.4, 22.2, 22.2, 21.9, 21.4, 19.4, 19.3, 17.8, 14.5, 11.0; HRMS (ESI) calcd for $C_{48}H_{79}O_{10}N_{12}^+$ $[M+H]^+$ 983.60366, found 983.60174

Synthesis of surugamide B analogs 7a and 7b

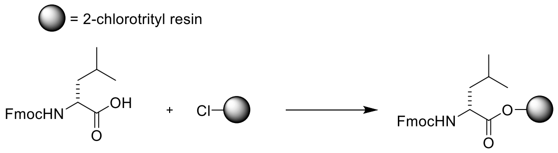

S1 S2

Fmoc-D-Leu-2-chlorotrityl resin (S2): 2-Chlorotrityl resin (111 mg, 0.149 mmol) in Libra tube was swollen with CH_2Cl_2, and then excess solvent was removed by filtration. To the resin were added a solution of Fmoc-D-Leu-OH (106 mg, 0.298 mmol) and *i*-Pr_2NEt (102 µL, 0.548 mmol) in CH_2Cl_2 (0.5 mL), and shaken for 30 min to give **S2**. The reaction mixture was washed with CH_2Cl_2 (× 5). Dried **S2** (5.80 mg) was added with 20% piperidine in DMF and stirred for 1 h. The supernatant was diluted with DMF and was subjected to UV measurement. Loading rate for **S2** was calculated to be 1.05 mmolg^{-1} from observed UV absorbance at 301 nm.

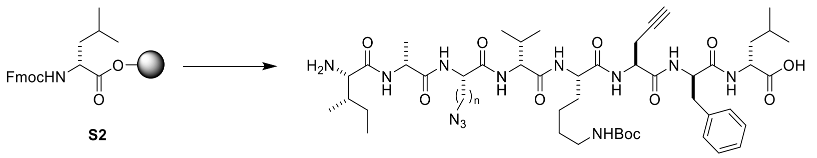

S2

S3a: n = 2
S3b: n = 4

Linear Peptide S3a and S3b: The resin **S2** (0.0275 mmol) in Libra tube was swelled in CH_2Cl_2 for 10 min, which was subjected to 7 cycles [Fmoc-D-Phe-OH, Fmoc-L-Pra-OH, Fmoc-L-Lys(Boc)-OH, Fmoc-D-Val-OH, Fmoc-L-Dab(N_3)-OH, Fmoc-D-Ala-OH, Fmoc-L-Ile-OH] of the SPPS protocol to afford resin-bound peptides. To the resins were added $CH_2Cl_2/(CF_3)_2CHOH$ (= 80:20) (5 mL), being stirred for 10 min, and then the reaction mixture was filtered. This procedure was repeated twice. The filtrates were concentrated to afford crude peptide **S3a**, which were used in the next reaction without further purification. **S3b** was synthesized from 0.0266 mmol of **S2** through the identical procedure, except for the use of Fmoc-L-Lys(N_3)-OH instead of Fmoc-L-Dab(N_3)-OH.

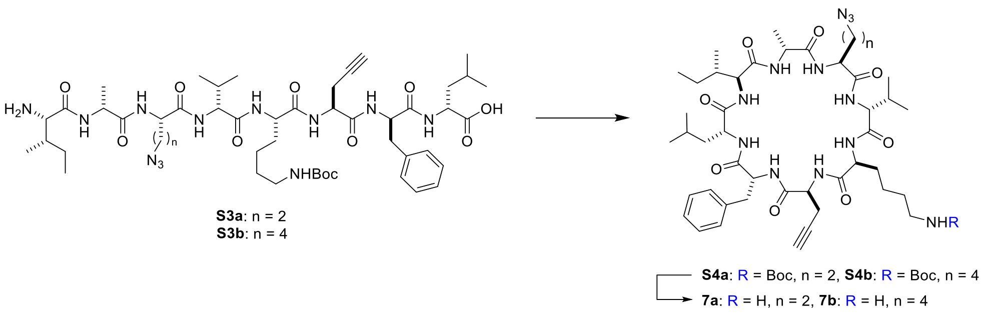

surugamide B analogs 7a and 7b: To a solution of peptide **S3a** (0.0250 mmol) in CH_2Cl_2/DMF (= 9:1) (25.0 mL) were added 2,6-dimethylpyridine (10.7 mg, 0.100 mmol), HOAt (6.81 mg, 0.0500 mmol) and PyBOP (26.0 mg, 0.0500 mmol). After being stirred overnight, the solvent was removed under vacuo to give crude **S4a**. To the residue was added a mixture of TFA/H_2O/*i*-Pr_3SiH (= 95:2.5:2.5) (1.00 mL), and the mixture was stirred for 1 hr. The reaction mixture was diluted with chilled Et_2O (24.0 mL), centrifuged at 3,500 × *g* for 10 min at 4 °C, and Et_2O layer was removed by decantation. The crude peptides were purified by HPLC with COSMOSIL 5C_{18}-MS-II 20 mm I.D× 250 mm, which were eluted by mobile phase MeCN:H_2O (= 45:55) containing 0.05 % TFA with flow rate at 10 ml/min to afford peptide **7a** (15.3 mg, 62.2 % for 17 steps). From **S3b**, the identical procedure afforded **7b** (14.3 mg, 58.4 % for 17 steps). Both **7a** and **7b** were obtained as colorless amorphous solids.

7a: $[\alpha]_D^{21}$ −12.50 (*c* 1.33, DMSO); ^{1}H NMR (400 MHz, DMSO-d_6) δ 8.43 (d, *J* = 6.4 Hz, 1H), 8.32-8.28 (m, 1H), 8.14 (m, 2H), 7.93-7.73 (m, 6H), 7.26-7.23 (m, 5H), 4.48 (m, 2H), 4.32 (s, 1H), 4.20-4.15 (m, 5H), 3.34 (m, 1H), 3.26-3.23 (m, 1H), 3.13 (m, 1H), 2.75 (m, 4H), 2.39 (m, 2H), 1.93-1.83 (m, 2H),1.72 (s, 3H), 1.57-1.48 (m, 6H), 1.33 (d, *J* = 7.2 Hz, 2H), 1.25-1.21 (m, 4H), 1.07 (m, 1H), 0.89-0.80 (m, 18H); ^{13}C NMR (100 MHz, DMSO-d_6) δ173.4, 172.8, 172.3, 172.2, 171.8, 171.6, 171.2, 170.1, 138.3, 129.7 (2×CH), 128.6 (2×CH), 126.8, 80.6, 73.5, 59.1, 57.3, 54.6, 53.3, 52.6, 52.6, 52.3, 50.4, 49.5, 48.0, 37.5, 36.5, 31.3, 31.3, 30.8, 26.9, 24.9, 24.7, 23.5, 22.8, 22.8, 22.1, 21.4, 19.8, 18.8, 18.3, 15.7, 11.2; HRMS (ESI) calcd for $C_{44}H_{69}O_8N_{12}^+$ $[M+H]^+$ 893.53558, found 893.53347

7b : $[\alpha]_D^{21}$ −26.00 (*c* 0.19, DMSO); ^{1}H NMR (400 MHz, DMSO-d_6) δ 8.38 (d, *J* = 10.0 Hz, 1H), 8.23-8.18 (m, 1H), 8.10 (t, *J* = 9.6 Hz, 2H), 7.93 (d, *J* = 5.2 Hz, 1H), 7.87 (s, 1H), 7.75 (s, 1H), 7.64 (s, 3H), 7.22 (m, 5H), 4.45 (s, 2H), 4.34 (s, 1H), 4.28-4.18 (m, 4H), 4.12 (d, *J* = 7.6 Hz, 1H), 3.12 (m 1H), 2.76 (m, 4H), 2.35 (s, 2H), 1.91 (s, 1H), 1.73 (m, 10H), 1.51 (m, 7H), 1.27 (m, 3H), 1.19 (d, *J* = 6.0 Hz, 2H), 1.05 (m, 1H), 0.89-0.80 (m, 18H); ^{13}C NMR (100 MHz, DMSO-d_6) δ 172.9, 172.5, 172.4, 172.2, 171.9, 171.4, 171.0, 170.2, 138.2, 129.7 (2×CH), 128.6 (2×CH), 126.8, 80.6, 73.5, 59.2, 57.2, 54.7, 53.1, 52.6, 52.4, 52.3, 51.1, 49.1, 46.4, 37.7, 36.9, 31.7, 31.6, 31.1, 28.2, 27.0, 26.4, 24.8, 24.7, 23.5, 23.0, 22.7, 22.3, 21.4, 19.8, 18.8, 18.6, 15.6, 11.4; HRMS (ESI) calcd for $C_{46}H_{73}O_8N_{12}^+$ $[M+H]^+$ 921.56688, found 921.56574

Synthesis of surugamide B bicyclic analogs 8a and 8b

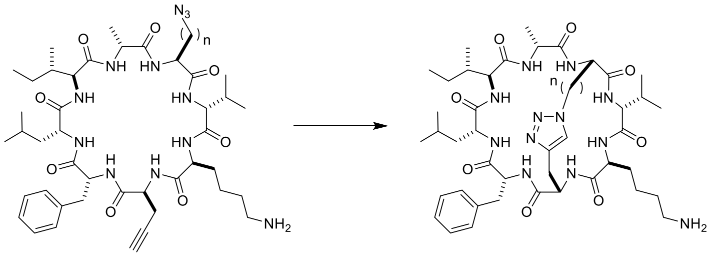

7a: n = 2, **7b**: n = 4 **8a**: n = 2, **8b**: n = 4

surugamide B bicyclic analogs 8a and 8b: To peptide **7a** (0.0170 mmol) dissolved in water (10.0 mL) were added $CuSO_4 \cdot 5H_2O$ (4 eq) and L-ascorbic acid sodium salt (6 eq). After being stirred overnight at 40 °C, the solvent was removed under vacuo to afford crude **8a**. The crude peptide was purified by HPLC with COSMOSIL $5C_{18}$-MS-II 20 mm I.D × 250 mm, which were eluted by mobile phase MeCN:H_2O (= 40:60), containing 0.05 % TFA with flow rate at 10 ml/min to afford peptide **8a** (3.1 mg, 20.4% from **7a**). From **7b** (0.0160 mmol), the identical procedure afforded **8b** (8.60 mg, 58.3% from **7b**). Both compounds were obtained as colorless amorphous solids.

8a: $[\alpha]_D^{20}$ −154.13 (*c* 0.32, DMSO); ^{1}H NMR (500 MHz, DMSO-d_6) δ 8.93 (s, 1H), 8.79 (d, *J* = 6.5 Hz, 1H), 8.52 (d, *J* = 8.0 Hz, 1H), 7.87 (d, *J* = 8.0 Hz, 1H), 7.70 (m, 3H), 7.24-7.12 (m, 7H), 6.81 (d, *J* = 8.0 Hz, 1H), 4.74 (m, 1H), 4.52-4.48 (m, 2H), 4.37 (t, *J* = 12.5 Hz, 1H), 4.27-4.21 (m, 3H), 4.10 (t, *J* = 8.0 Hz, 2H), 3.90 (s, 1H), 3.51 (s, 1H), 3.15 (d, *J* = 14.0 Hz, 1H), 2.96-2.93 (m, 1H), 2.77 (d, *J* = 6.5 Hz, 3H), 2.66 (m, 1H), 2.36 (s, 1H), 2.07-2.00 (m, 1H), 1.81 (m, 2H), 1.68-1.39 (m, 8H), 1.28-1.23 (m, 2H), 1.14 (d, *J* = 7.5 Hz, 3H), 1.04-0.98 (m, 3H),0.92-0.83 (m, 16H); ^{13}C NMR (125 MHz, DMSO-d_6) δ 173.4, 173.3, 172.4, 171.5, 171.1, 170.9, 170.7, 170.2, 140.4, 137.9, 129.9 (2×CH), 128.5 (2×CH), 126.9, 124.5, 70.3, 59.1, 57.5, 54.8, 53.9, 53.1, 52.3, 51.8, 50.6, 45.5, 39.1, 37.0, 34.4, 31.5, 30.8, 30.2, 29.6, 27.0, 24.7, 24.3, 23.6, 23.2, 21.9, 19.2, 19.1, 18.2, 16.4, 11.4; HRMS (ESI) calcd for $C_{44}H_{69}O_8N_{12}^+$ $[M+H]^+$ 893.53558, found 893.53278

8b: $[\alpha]_D^{21}$ +10.28 (*c* 0.63, DMSO); ^{1}H NMR (500 MHz, DMSO-d_6) δ 8.71 (m, 2H), 8.17 (s, 1H), 7.78-7.72 (m, 5H), 7.50 (d, *J* = 7.5 Hz, 2H), 7.34 (t, *J* = 7.5 Hz, 2H), 7.28 (d, *J* = 7.5 Hz, 1H), 7.23-7.14 (m, 2H), 6.73 (m, 1H), 4.66 (m, 1H), 4.56 (s, 1H), 4.41 (t, *J* = 8.5 Hz, 1H), 4.26-4.16 (m, 3H), 4.10-4.05 (m, 1H), 3.96 (d, *J* = 13.0 Hz, 1H), 3.10 (d, *J* = 13.0 Hz, 1H), 2.84-2.72 (m, 4H), 2.67-2.63 (m, 1H), 1.91-1.86 (m, 1H), 1.74-1.70 (m, 4H), 1.64-1.34 (m, 9H), 1.22 (d, *J* = 7.0 Hz, 4H), 1.18-1.10 (m, 2H), 0.89-0.75 (m, 22H); ^{13}C NMR (125 MHz, DMSO-d_6) δ173.9, 171.7, 171.4, 171.3, 171.3, 171.2, 171.2, 170.5, 142.4, 139.6, 130.4 (2×CH), 128.9 (2×CH), 127.3, 122.1, 63.6, 59.1, 57.3, 56.4, 55.5, 51.7,

50.6, 49.6, 48.5, 42.1, 39.0, 35.1, 34.4, 32.3, 32.0, 31.3, 30.3, 29.3, 27.0, 25.5, 24.9, 23.7, 23.2, 22.5, 22.4, 19.3, 19.2, 17.5, 15.5, 10.9; HRMS (ESI) calcd for $C_{46}H_{73}O_8N_{12}^+$ $[M+H]^+$ 921.56688, found 921.56455

Synthesis of *seco*-wollamide B1-EG analog 9a and 9b

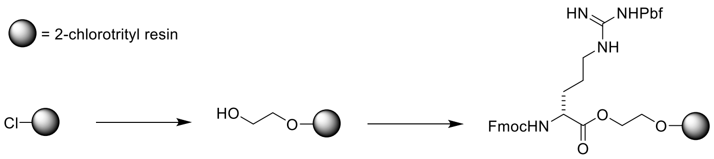

S1 4 Fmoc-D-Arg-**4**

Fmoc-D-Arg(Pbf)-4: 2-chlorotrityl chloride resin **S1** (80.0 mg, 0.101 mmol) in Libra tube was swelled with CH_2Cl_2 for 10 min, and then excess solvent was removed by filtration. To the resin was added a solution of ethylene glycol (EG) (12.5 mg, 0.202 mmol), and *i*-Pr_2NEt (26.1 mg, 0.202 mmol) in CH_2Cl_2 (2.0 mL) and shaken for 2 h at 37 °C to give EG-2-chlorotrityl resin **4**. To the resin was added a solution of Fmoc-D-Arg(Pbf)-OH (131.1 mg, 0.202 mmol), DIC (63.3 μL, 0.404 mmol) and DMAP (1.23 mg, 0.0101 mmol) in CH_2Cl_2 (2.0 mL) and stirred for 3 h at 37 °C to give Fmoc-D-Arg(Pbf)-**4**. Dried Fmoc-D-Arg(Pbf)-**4** was added with 20% piperidine in DMF and stirred for 1 h. The supernatant was diluted with DMF and was subjected to UV measurement. Loading rate for Fmoc-D-Arg(Pbf)-**4** was calculated to be 0.367 $mmolg^{-1}$ based on observed UV absorbance at 301 nm.

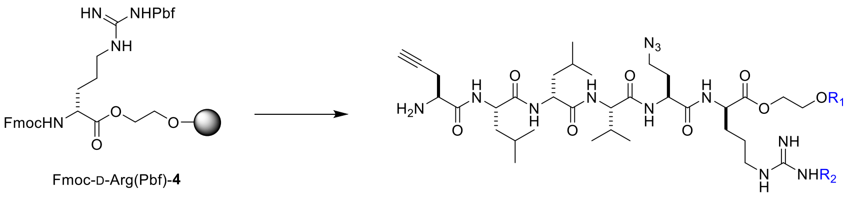

S5a: R_1 = resin, R_2 = Pbf ⟶ **9a**: R_1 = R_2 = H

***seco*-wollamide B1-EG analogs 9a**: Fmoc-D-Arg(Pbf)-**4** (0.0202 mmol) in Libra tube was swelled in CH_2Cl_2 for 10 min, which was subjected to 5 cycles [Fmoc-L-Dab(N_3)-OH, Fmoc-L-Val-OH, Fmoc-D-Leu-OH, Fmoc-L-Leu-OH, Fmoc-L-Pra-OH] of SPPS protocol to afford resin bound peptides. To the peptide **S5a** were added TFA/H_2O/*i*-Pr_3SiH = 95:2.5:2.5 (1.0 mL), being shaken for 30 min, and then the reaction mixture was filtered. The filtrate was diluted with Et_2O (25 mL) and was chilled (–80 °C), then centrifuged with 3,500 × *g* for 10 min at 4 °C to afford crude **9a**. The crude peptides were purified by HPLC with COSMOSIL $5C_{18}$-MS-II 20 mm I.D × 250 mm, which were eluted by mobile phase MeCN:H_2O (= 28:72), containing 0.05 % TFA with flow rate at 10 ml/min to afford peptide **9a** (10.2 mg, 66.0 % for 15 steps). **9a** were obtained as a colorless amorphous solid.

9a: $[\alpha]_D^{17}$ –8.91 (*c* 0.92, DMSO); ^1H NMR (500 MHz, DMSO-d_6) δ 8.58-8.52 (m, 1H), 8.36-8.26 (m,

3H), 8.15-8.11 (m, 2H), 7.94 (d, J = 29.7 Hz, 1H), 7.66-7.36 (m, 4H), 4.36 (d, J = 5.1 Hz, 3H), 4.20 (s, 1H), 4.12 (s, 1H), 4.01-3.97 (m, 3H), 3.52-3.46 (m, 3H), 3.36-3.23 (m, 2H), 3.05-2.98 (m, 3H), 2.69-2.59 (m, 2H), 1.96-1.92 (m, 1H), 1.86-1.83 (m, 1H), 1.78-1.71 (m, 2H), 1.58-1.37 (m, 8H), 1.23-1.19 (m, 1H), 0.83-0.75 (m, 18H); ^{13}C NMR (125 MHz, DMSO-d_6) δ 172.1, 171.6, 171.1, 171.1, 171.0, 166.9, 157.0, 77.7, 75.3, 69.8, 66.3, 58.8, 51.7, 51.1, 50.6, 50.1, 47.4, 41.5, 41.4, 41.2, 31.3, 31.3, 30.3, 28.1, 25.0, 24.2, 24.1, 23.0, 22.8, 21.7, 21.4, 21.1, 19.2, 18.0; HRMS (ESI) calcd for $C_{34}H_{61}O_8N_{12}^+$ $[M+H]^+$ 765.47298, found 765.47236

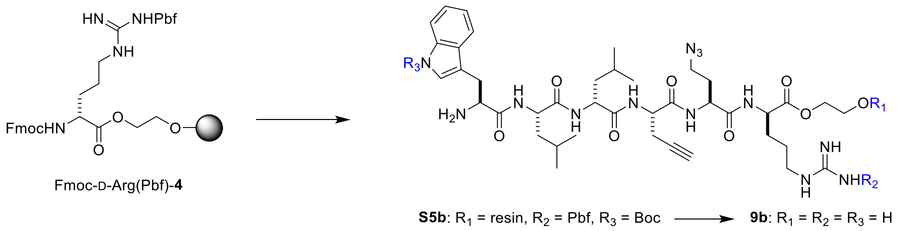

***seco*-wollamide B1-EG analogs 9b**: Fmoc-D-Arg(Pbf)-**4** (0.0204 mmol) in Libra tube was swelled in CH_2Cl_2 for 10 min, which was subjected to 5 cycles [Fmoc-L-Dab(N_3)-OH, Fmoc-L-Pra-OH, Fmoc-D-Leu-OH, Fmoc-L-Leu-OH, Fmoc-L-Trp(Boc)-OH] of SPPS protocol to afford resin bound peptides. To the peptide **S5b** were added TFA/H_2O/*i*-Pr_3SiH = 95:2.5:2.5 (1.0 mL), being shaken for 30 min, and then the reaction mixture was filtered. The filtrate was diluted with Et_2O (25 mL) and was chilled (–80°C), then centrifuged with 3,500 × *g* for 10 min at 4 °C to afford crude **9b**. The crude peptides were purified by HPLC with COSMOSIL 5C_{18}-MS-II 20 mm I.D × 250 mm, which were eluted by mobile phase MeCN:H_2O (= 32.5:67.5), containing 0.05 % TFA with flow rate at 10 ml/min to afford peptide **9b** (15.5 mg, 89.2 % for 15 steps). **9b** were obtained as a colorless amorphous solid.

9b: $[\alpha]_D^{19}$ –0.90 (*c* 1.25, DMSO); ^{1}H NMR (400 MHz, DMSO-d_6) δ 10.90 (d, J = 22.0 Hz, 1H), 8.75 (s, 1H), 8.42-8.22 (m, 4H), 7.96 (m, 3H), 7.73-7.64 (m, 1H), 7.39-7.30 (m, 3H), 7.17-6.99 (m, 4H), 4.48-4.20 (m, 5H), 4.04 (d, J = 4.9 Hz, 3H), 3.55-3.50 (m, 3H), 3.26 (d, J = 11.6 Hz, 3H), 3.06-2.99 (m, 3H), 2.80-2.64 (m, 2H), 1.85-1.23 (m, 13H), 1.03-0.83 (m, 12H); ^{13}C NMR (100 MHz, DMSO-d_6) δ 172.9, 172.2, 172.0, 170.6, 169.0, 168.8, 157.6, 136.9, 127.5, 125.7, 121.7, 119.1, 118.9, 112.0, 107.4, 81.1, 73.3, 66.8, 59.4, 53.1, 53.0, 52.2, 52.0, 50.9, 47.8, 41.9, 41.6, 41.5, 31.8, 28.6, 27.9, 25.5, 24.7, 24.7, 23.6, 23.5, 22.3 (2×CH_3), 22.0 (2×CH_3); HRMS (ESI) calcd for $C_{40}H_{62}O_8N_{13}^+$ $[M+H]^+$ 852.48388, found 852.48361

Synthesis of monocyclic peptides by CuAAC

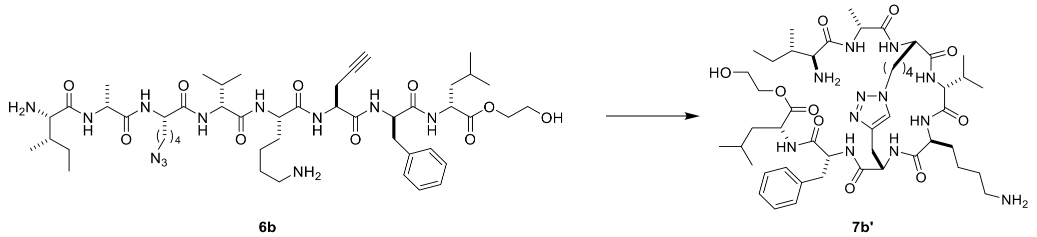

6b → 7b'

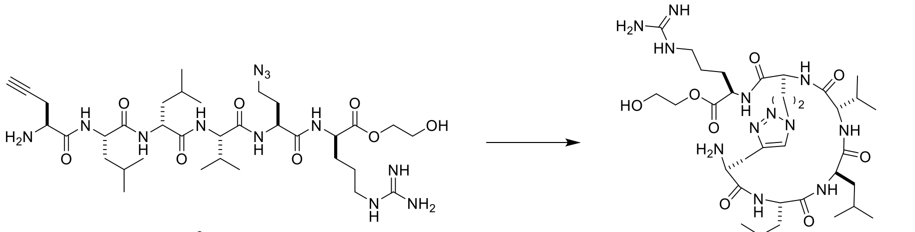

9a → 10a'

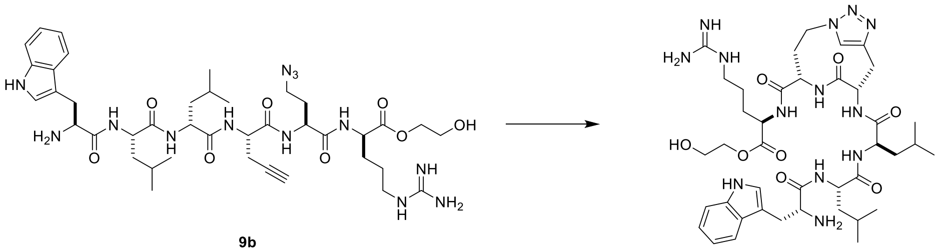

9b → 10b'

Monocyclic peptide **7b'**, **10a'**, and **10b'**: To peptide **6b** (0.005 mmol) dissolved in water (3.0 mL) were added $CuSO_4 \cdot 5H_2O$ (5 eq) and L-ascorbic acid sodium salt (5 eq). After being stirred overnight at 40 °C, the solvent was removed under vacuo to afford crude **7b'**. The crude peptide was purified by HPLC with COSMOSIL 5C$_{18}$-MS-II 20 mm I.D×250 mm, which were eluted by mobile phase MeCN:H_2O (= 30:70), containing 0.05 % TFA with flow rate at 10 ml/min to afford peptide **7b'** (1.9 mg, 38.6% from **6b**). From **9a** (0.005 mmol), the identical procedure afforded **10a'** (2.60 mg, 67.9% from **9a**). From **9b** (0.005 mmol), the identical procedure afforded **10b'** (0.30 mg, 7.03% from **9b**). All compounds were obtained as colorless amorphous solids.

Synthesis of *seco*-BC1-EG analog 12

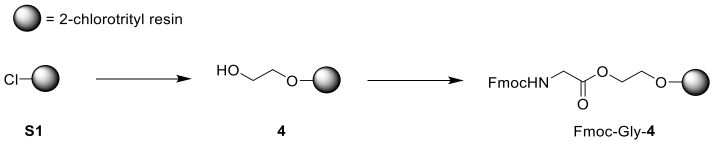

Fmoc-Gly-4: 2-chlorotrityl resin **S1** (132 mg, 0.178 mmol) in Libra tube was swelled with CH_2Cl_2 for 10 min, and then excess solvent was removed by filtration. To the resin was added a solution of ethylene glycol (EG) (22.1 mg, 0.356 mmol), and *i*-Pr_2NEt (46.0 mg, 0.356 mmol) in CH_2Cl_2 (2.0 mL) and shaken for 2 h at 37 °C to give EG-2-chlorotrityl resin **4**. To the resin was added a solution of Fmoc-Gly-OH (106 mg, 0.356 mmol), DIC (112 µL, 0.712 mmol) and DMAP (2.17 mg, 0.0178 mmol) in CH_2Cl_2 (2.0 mL) and stirred for 3 h at 37 °C to give Fmoc-Gly-**4**. Dried resin was added with 20% piperidine in DMF and stirred for 1 h. The supernatant was diluted with DMF and was subjected to UV measurement. Loading rate for Fmoc-Gly-**4** was calculated to be 0.513 mmolg^{-1} based on UV absorbance at 301 nm.

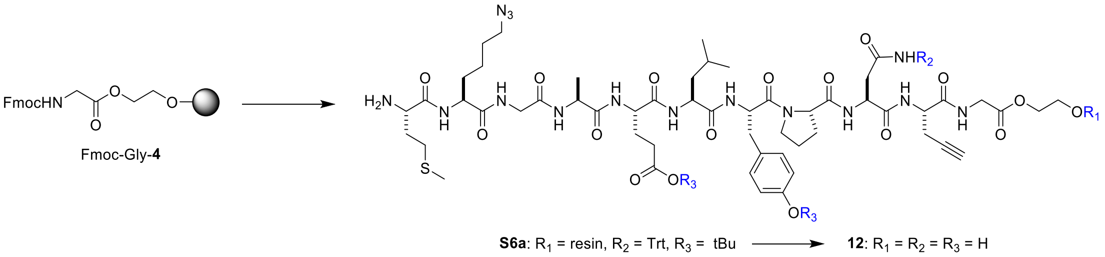

***seco*-BC1-EG analog (12)**: Fmoc-Gly-**4** (0.025 mmol) in Libra tube was swelled in CH_2Cl_2 for 10 min, which was subjected to 10 cycles [Fmoc-L-Pra-OH, Fmoc-L-Asn(Trt)-OH, Fmoc-L-Pro-OH, Fmoc-L-Tyr(tBu)-OH, Fmoc-L-Leu-OH, Fmoc-L-Glu(tBu)-OH, Fmoc-L-Ala-OH, Fmoc-Gly-OH, Fmoc-L-Lys(N_3)-OH, Fmoc-L-Met-OH] of SPPS protocol to afford resin bound peptides **S6a**. To the peptide **S6a** were added TFA/H_2O/*i*-Pr_3SiH = 95:2.5:2.5 (1.0 mL), being shaken for 30 min, and then the reaction mixture was filtered. The filtrate was diluted with chilled Et_2O (25 mL), then centrifuged with 3,500 × *g* for 10 min at 4 °C to afford crude peptides. The crude peptide was purified by reversed phase HPLC with COSMOSIL 5C_{18}-MS-II 20 mm I.D × 250 mm, which was eluted by mobile phase MeCN:H_2O (= 30:70) containing 0.05 % TFA with flow rate at 10 ml/min, to afford peptide **12** (26.8 mg, 86.2% for 21 steps) as a colorless amorphous solid.

***seco*-BC1-EG analog (12)**: $[\alpha]_D^{21}$ −20.89 (*c* 1.62, DMSO); ^{1}H NMR (400 MHz, DMSO-d_6) δ 8.61 (d, *J* = 7.2 Hz, 1H), 8.48-8.43 (m, 1H), 8.32 (t, *J* = 5.2 Hz, 1H), 8.17-8.07 (m, 6H), 7.99 (d, *J* = 7.2 Hz, 1H), 7.75 (d, *J* = 8.4 Hz, 1H), 7.49 (s, 1H), 7.07-6.94 (m, 3H), 6.63 (d, *J* = 8.0 Hz, 2H), 4.55 (d, *J* = 6.8 Hz, 2H), 4.41-4.20 (m, 6H), 4.04 (t, *J* = 4.8 Hz, 1H), 3.93-3.50 (m, 6H), 3.56 (t, *J* = 4.8 Hz, 4H),

3.31 (t, J = 6.8 Hz, 4H), 2.89 (m, 1H), 2.81-2.78 (m, 1H), 2.71-2.54 (m, 3H), 2.50-2.34 (m, 10H), 2.27-2.14 (m, 1H), 2.03-1.60 (m, 10H), 1.52 (m, 3H), 1.34 (m, 3H), 1.17 (d, J = 6.8 Hz, 3H), 0.81 (dd, J = 16.8, 6.4 Hz, 6H); ^{13}C NMR (100 MHz, DMSO-d_6) δ 173.9, 172.1, 171.8, 171.5, 171.3, 171.2, 170.6, 170.5, 170.0, 169.9, 169.2, 168.0, 167.9, 155.6, 130.0 (2×CH), 127.6, 114.8 (2×CH), 80.3, 72.7, 66.1, 59.3, 58.7, 52.5, 52.2, 51.6, 51.4, 51.3, 50.7, 50.4, 49.5, 47.8, 46.6, 41.6, 40.6, 40.2, 36.8, 35.6, 31.3, 30.9, 30.0, 28.8, 27.9, 27.7, 26.9, 24.3, 23.9, 23.0, 22.4, 21.3, 21.2, 18.2, 14.3; HRMS (ESI) calcd for $C_{54}H_{81}O_{17}N_{15}SNa^+$ $[M+Na]^+$ 1266.55478, found 1266.55176

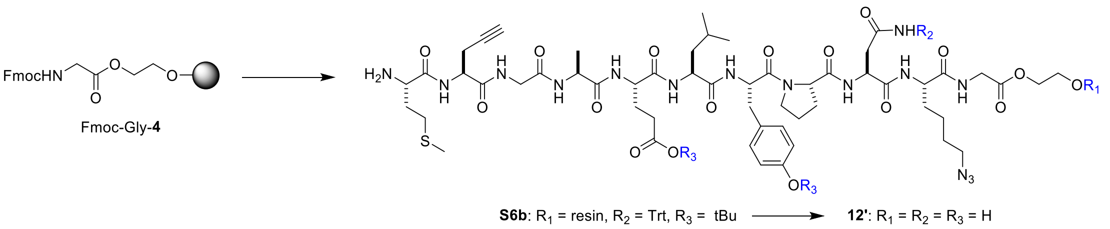

***seco*-BC1-EG analog (12')**: Fmoc-Gly-**4** (0.025 mmol) in Libra tube was swelled in CH_2Cl_2 for 10 min, which was subjected to 10 cycles [Fmoc-L-Lys(N_3)-OH, Fmoc-L-Asn(Trt)-OH, Fmoc-L-Pro-OH, Fmoc-L-Tyr(tBu)-OH, Fmoc-L-Leu-OH, Fmoc-L-Glu(tBu)-OH, Fmoc-L-Ala-OH, Fmoc-Gly-OH, Fmoc-L-Pra-OH, Fmoc-L-Met-OH] of SPPS protocol to afford resin bound peptides **S6b**. To the peptide **S6b** were added TFA/H_2O/*i*-Pr_3SiH = 95:2.5:2.5 (1.0 mL), being shaken for 30 min, and then the reaction mixture was filtered. The filtrate was diluted with chilled Et_2O (25 mL), then centrifuged with 3,500 × *g* for 10 min at 4 °C to afford crude peptides. The crude peptide was purified by reversed phase HPLC with COSMOSIL 5C_{18}-MS-II 20 mm I.D×250 mm, which was eluted by mobile phase MeCN:H_2O (= 30:70) containing 0.05 % TFA with flow rate at 10 ml/min, to afford peptide **12'** (20.3 mg, 64.2% for 21 steps) as a colorless amorphous solid.

***seco*-BC1-EG analog (12')**: $[\alpha]_{20}^{D}$ –40.56 (*c* 2.01, MeOH); ^{1}H NMR (500 MHz, DMSO-d_6) δ 9.21 (d, J = 44.2 Hz, 1H), 8.90-8.83 (m, 1H), 8.55-7.72 (m, 10H), 7.43 (d, J = 38.8 Hz, 1H), 7.18-6.94 (m, 3H), 6.66-6.62 (m, 2H), 4.82 (s, 1H), 4.55-4.45 (m, 3H), 4.33-4.22 (m, 5H), 4.05-3.69 (m, 8H), 3.57-3.50 (m, 3H), 3.38-3.26 (m, 4H), 3.16 (s, 1H), 2.92-2.87 (m, 2H), 2.70-2.57 (m, 4H), 2.30-2.19 (m, 2H), 2.07-1.73 (m, 11H), 1.57-1.23 (m, 9H), 1.17 (d, J = 6.4 Hz, 3H), 0.82 (dd, J = 21.3, 6.4 Hz, 6H); ^{13}C NMR (125 MHz, DMSO-d_6) δ 174.6, 172.7, 172.5, 172.4, 172.1, 171.9, 171.2, 171.1, 170.5, 170.2, 170.1, 168.8, 168.5, 156.3, 130.6 (2×CH_2), 128.2, 115.5 (2×CH_2), 80.9, 73.9, 66.7, 60.0, 59.4, 52.8, 52.7, 52.5, 52.3, 52.1, 51.1, 50.3, 48.5, 47.3, 42.5, 41.3, 41.1, 37.3, 37.3, 36.3, 31.6, 31.6, 30.7, 29.4, 28.6, 28.3, 27.5, 24.9, 24.6, 23.6, 22.8, 22.0 (2 × CH_2), 18.9, 15.0; HRMS (ESI) calcd for $C_{54}H_{82}N_{15}O_{17}S^+$ $[M+H]^+$ 1244.57283, found 1244.57171.

Table S1. Enzymes used in this study.

Enzyme	Description	Amino acid sequence	Reference
N-His6-SurE	Amino acid sequence is derived from SurE (GenBank: BBZ90014.1). Sequence from the expression vector pET28a is underlined.	MGSSHHHHHHSSGLVPRGSHMGAEGAER<br>DAVGALFEELVREHRVTGAQLSVYRDGA<br>LSEYATGLASVRTGEPVTPRTGFPFGSVT<br>KFLTAELVMQFVCDGDLDLDDPLAGLLP<br>DLGRAAGPPLGTATVRQLLSHTAGVVDSI<br>EYDEMRGPSYRRFAAACARQPALFPPGL<br>AFSYSNTGYCLLGAVIEAASGMDWWTA<br>MDSCLLRPLGIEPAFLHDPRPGQGGAARP<br>VAEGHALRAGGERAEHVDHMASLSLAA<br>AGGLVGSATDLVTAARPHLADRKTFAQH<br>DLLPEDAVLAMRTCVPDAEPFGLADGWG<br>LGLMRHGTGDGAWYGHDGAVGGASCNL<br>RIHPDRSLALALTANSTAGPKLWEALVAR<br>LPEAGLDVGHYALPVPDSAPLAPDAGHL<br>GTYANGDLELMVTHDAAGDLFLTRESYS<br>DYRLSLHEDDLFVARSGEPGALPITGRFV<br>REHPAGPVALLQYGGRAMHRL	[2]
N-His6-WolJ	Amino acid sequence are derived from SurE (GenBank: UNO41476.1). Sequence from the expression vector pColdII is underlined.	MNHKVHHHHHHMSAEAKGRGTTISLPLD<br>RLAKEHSVVGGQLAVHHQGTTHTWAFG<br>EEEHDSGRPVSVGTAFALGSTTKALTATS<br>VLQLVSDGDLDLDSPVKTWIEERGAAAD<br>HPALNATLRQLLSHTAGLPSDHFDEEAPS<br>LRRWLAGFLTSGDERARASWPTPGSFSYS<br>NIGYAVAGRAVEAATGLPWWQAVRD<br>YLLTPLGTGIGLLPGAPQDGRPLTAAPGH<br>TAHTTPDGRTVVHVVQSTTDAGSAPSGG<br>LAGSAADLVRFARLHLEEPGDLDRSAVA<br>DPSVLREMAQPVTGAEPFGLADGWGLGL<br>GHFGPAGRHWLGHDGTLDGSTCHLRIEP<br>RQGTVVALTTNTSSGLALWDSVVEELRE<br>CGLDIGLHAPQPPLAAPAATFAECTGTYR<br>NGDLAVTVGIEDQHIVLGLPNGQRDSVAP<br>HTGLFFSGRPGNASLFLGRFVKDVATGEI<br>RALQYSGRTLTREALTARN	[1]
N-His6-SurE^{G235L}	G235L mutants of SurE. Sequence from the expression vector pET28a is underlined.	MGSSHHHHHHSSGLVPRGSHMGAEGAER<br>DAVGALFEELVREHRVTGAQLSVYRDGA<br>LSEYATGLASVRTGEPVTPRTGFPFGSVT<br>KFLTAELVMQFVCDGDLDLDDPLAGLLP<br>DLGRAAGPPLGTATVRQLLSHTAGVVDSI<br>EYDEMRGPSYRRFAAACARQPALFPPGL<br>AFSYSNTGYCLLGAVIEAASGMDWWT<br>AMDSCLLRPLGIEPAFLHDPRPGQGGAAR<br>PVAEGHALRAGGERAEHVDHMASLSLAA<br>ALGLVGSATDLVTAARPHLADRKTFAQH<br>DLLPEDAVLAMRTCVPDAEPFGLADGWG<br>LGLMRHGTGDGAWYGHDGAVGGASCNL<br>RIHPDRSLALALTANSTAGPKLWEALVAR<br>LPEAGLDVGHYALPVPDSAPLAPDAGHL<br>GTYANGDLELMVTHDAAGDLFLTRESYS<br>DYRLSLHEDDLFVARSGEPGALPITGRFV<br>REHPAGPVALLQYGGRAMHRL	[1]

Table S2. Compounds list

Compound	Description
**1**	surugamide B
**2**	wollamide B1
**3**	BC1
**4**	ethylene glycol bound to Trt(2-Cl) resin
**5a**	resin bound **6a** with protecting groups
**5b**	resin bound **6b** with protecting groups
**6a**	*seco*-surugamide B-EG analog harboring Dab(N_3)3 and Pra6
**6b**	*seco*-surugamide B-EG analog harboring Lys(N_3)3 and Pra6
**7a**	surugamide B analog harboring Dab(N_3)3 and Pra6
**7b**	surugamide B analog harboring Lys(N_3)3 and Pra6
**7b'**	**6b** derivative cyclized by CuAAC
**8a**	bicyclic analog of surugamide B with a triazole linkage between Dab(N_3)3 and Pra6
**8b**	bicyclic analog of surugamide B with a triazole linkage between Lys(N_3)3 and Pra6
**9a**	*seco*-wollamide B1-EG analog harboring Pra1 and Dab(N_3)5
**9b**	*seco*-wollamide B1-EG analog harboring Pra4 and Dab(N_3)5
**10a**	wollamide B1 analog with Pra1 and Dab(N_3)5
**10a'**	**9a** derivative cyclized by CuAAC
**10b**	wollamide B1 analog with Pra4 and Dab(N_3)5
**10b'**	**9b** derivative cyclized by CuAAC
**11a**	Bicyclic analog of wollamide B1 with a triazole linkage between Pra1 and Dab(N_3)5
**11a'**	**10a'** derivative without EG leaving group
**11b**	Bicyclic analog of wollamide B1 with a triazole linkage between Pra4 and Dab(N_3)5
**11b'**	**10b'** derivative without EG leaving group
**12**	*seco*-BC1-EG harboring Lys(N_3)2 and Pra10
**12'**	*seco*-BC1-EG harboring Pra2 and Lys(N_3)10
**13**	cyclic **12**
**13'**	cyclic **12'**
**14**	BC1 analog with triazole linkage between Lys(N_3)2 and Pra10
**14'**	BC1 analog with triazole linkage between Pra2 and Lys(N_3)10
**S1**	Trt(2-Cl) resin
**S2**	Fmoc-D-Leu bound **S1**
**S3a**	*seco*-surugamide B analog with harboring Dab(N_3)3 and Pra6
**S3b**	*seco*-surugamide B analog with harboring Lys(N_3)3 and Pra6
**S4a**	**7a** with protecting groups
**S4b**	**7b** with protecting groups
**S5a**	resin bound **9a** with protecting groups
**S5b**	resin bound **9b** with protecting groups
**S6a**	resin bound **12** with protecting groups
**S6b**	resin bound **12'** with protecting groups

(a)

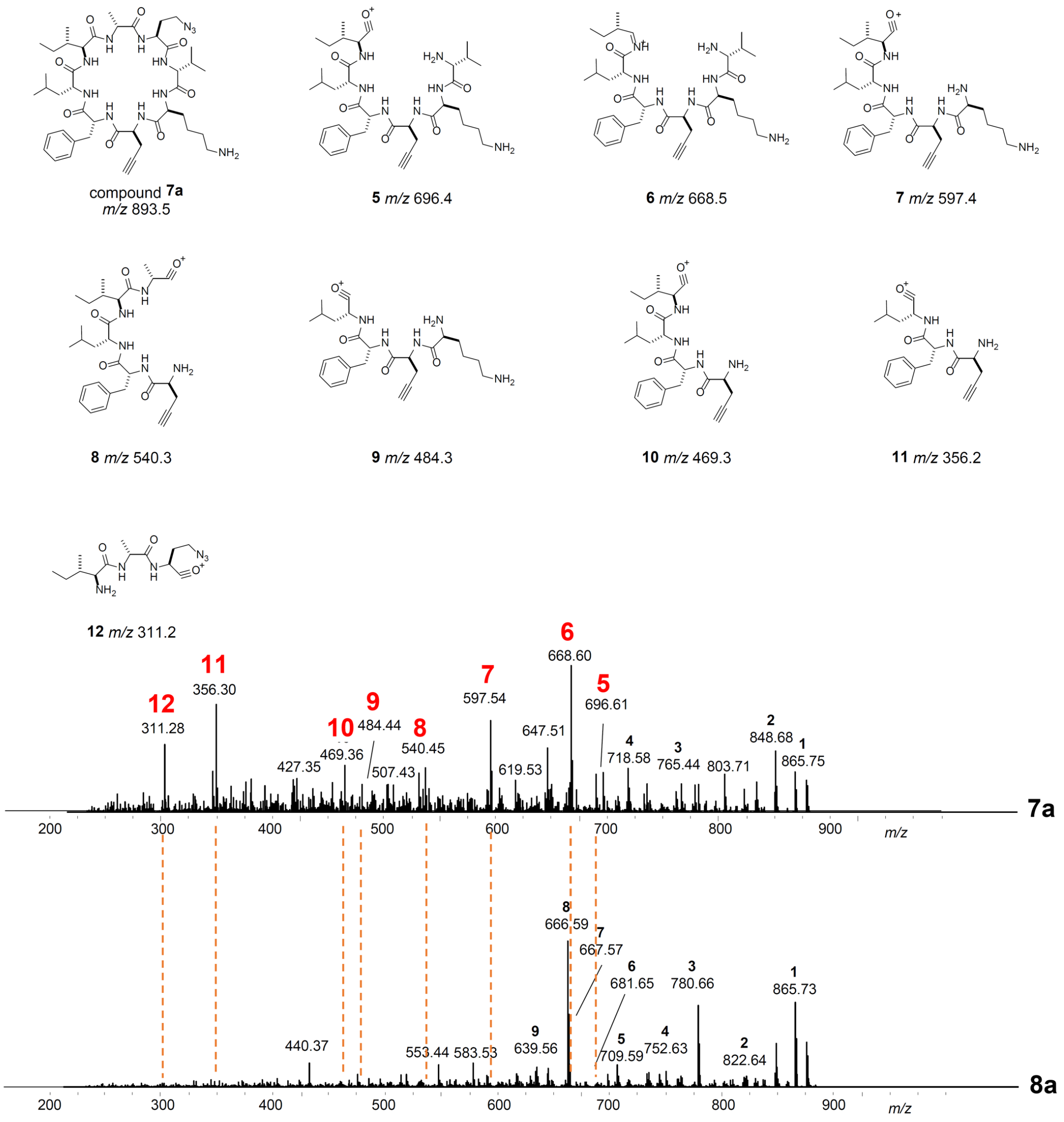

(Figure continues to the next page)

(b)

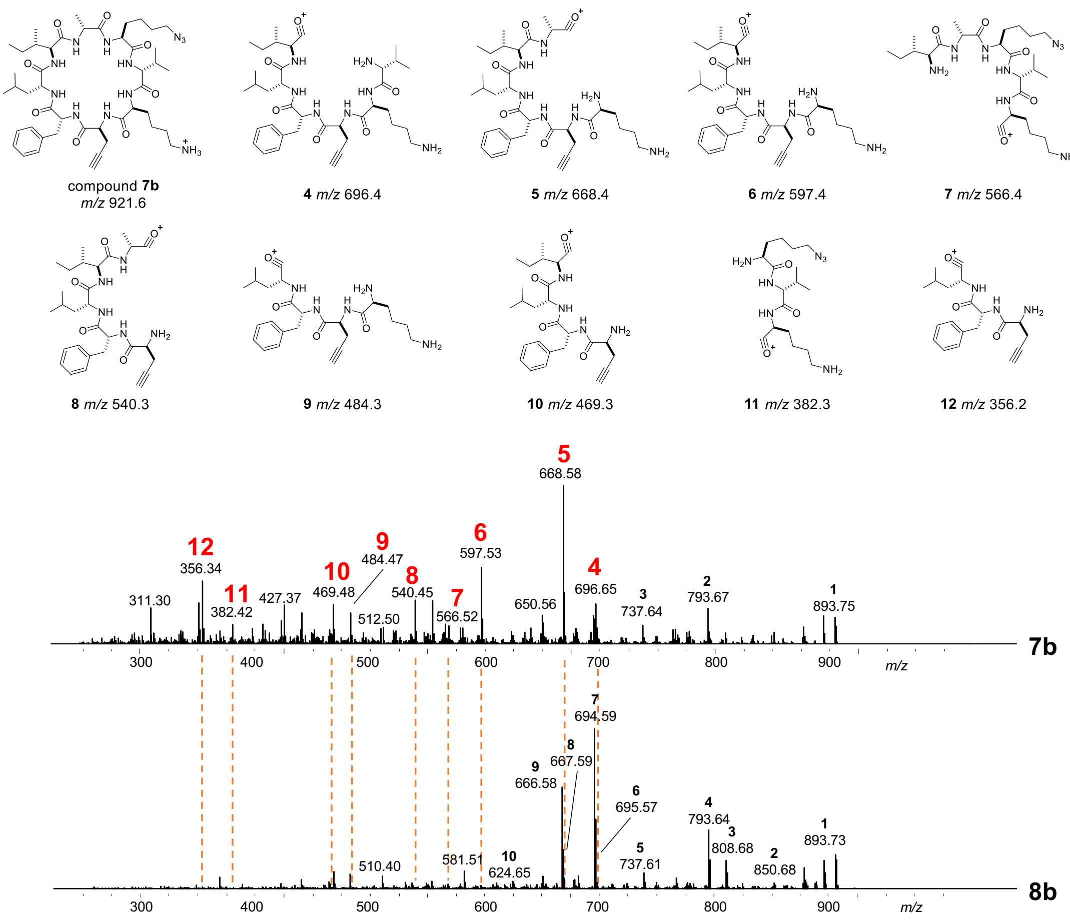

Figure S1. MS/MS spectra of monocyclic peptides (**7a**/**7b**), and bicyclic peptides (**8a**/**8b**). Among the fragment ions derived from monocyclic peptides **7a** and **7b**, the fragment ions containing either L-Pra or L-Dab(N_3)/L-Lys(N_3) were highlighted in red. The assigned structures of each fragment were shown. (a) MS/MS spectra of **7a**/**8a**. (b) MS/MS spectra of **7b**/**8b**.

Fragment ions that contain both L-Pra and L-Dab(N_3)/L-Lys(N_3) were not considered as indications for intramolecular CuAAC, because the formation of triazole does not change *m/z* value, therefore, fragment ions with identical *m/z* value could be observed before and after CuAAC. On the other hand, disappearance of the fragment ion containing either L-Pra OR L-Dab(N_3)/L-Lys(N_3) could be a good indicator for intramolecular CuAAC. Consistently, highlighted fragments disappeared after CuAAC (see dashed orange lines). Extended version of these figures are shown in Figure S11-S14.

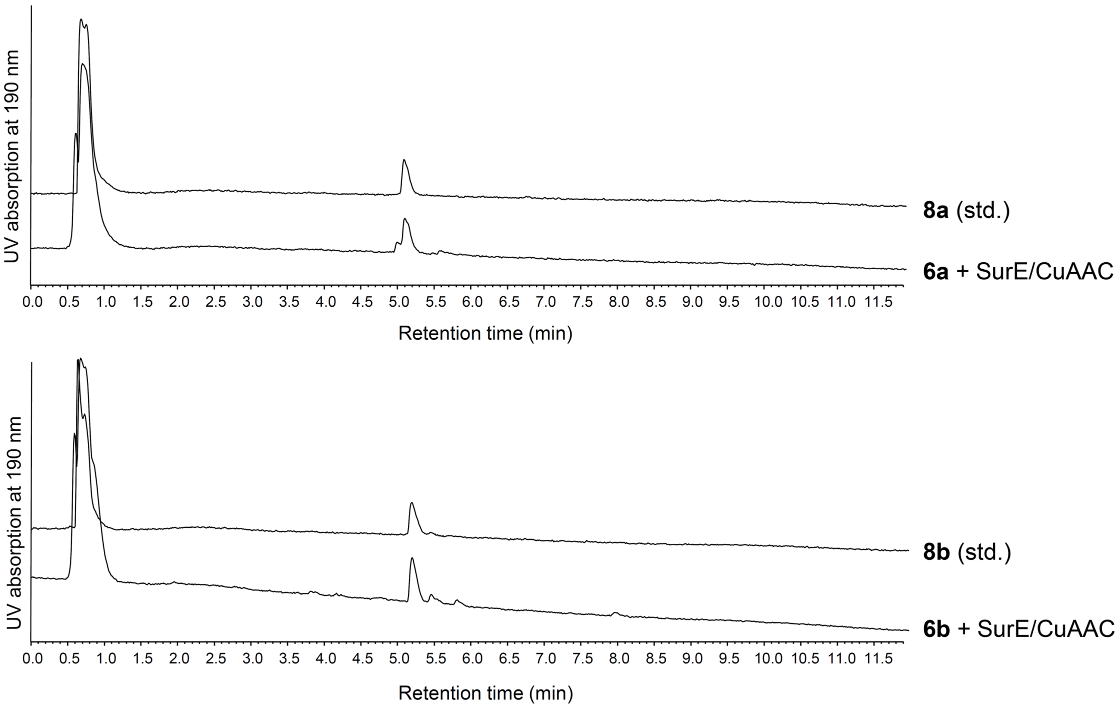

Figure S2. HPLC comparison of chemoenzymatically synthesized **8a**/**8b** with synthetic standards.

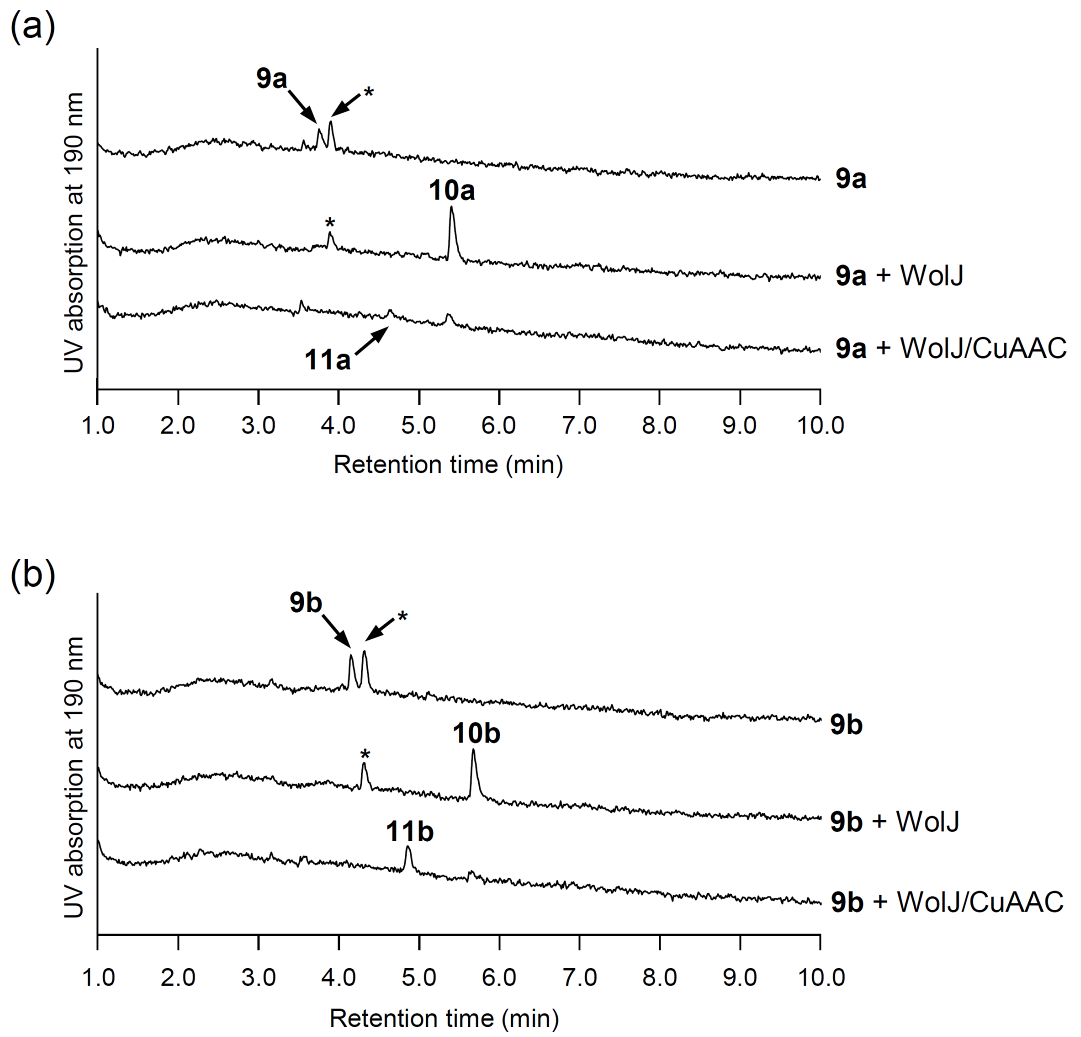

Figure S3. Chemoenzymatic tandem cyclization of wollamide sequences. Samples were analyzed by HPLC. Column elutes were monitored with UV absorption at 190 nm. **9a**/**9b** underwent non-enzymatic spontaneous hydrolysis to release ethylene glycol moieties during incubation. Resultant linear peptides were highlighted by asterisks (*).

a) top: **9a** without WolJ, middle: **9a** with WolJ, bottom: **9a**+WolJ reaction mixture treated with CuAAC.

b) top: **9b** without WolJ, middle: **9b** with WolJ, bottom: **9b**+WolJ reaction mixture treated with CuAAC.

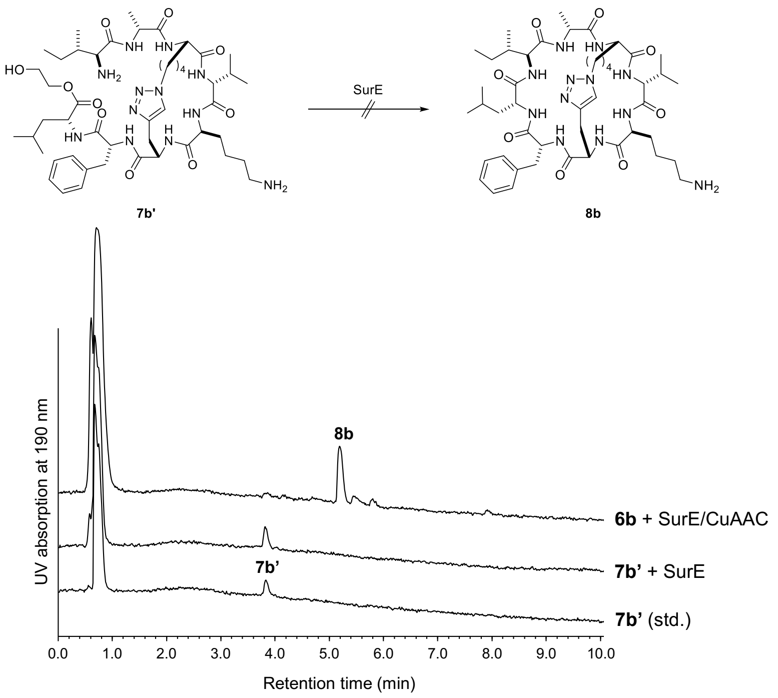

Figure S4. SurE-reaction on CuAAC-cyclized peptide **7b'**. The pre-cyclized peptide **7b'** remained intact after incubation, as it was neither cyclized nor hydrolyzed by SurE. Chromatogram of a reaction mixture of chemoenzymatic tandem cyclization (i.e. 1st SurE, 2nd CuAAC) on linear peptide **6b** is shown on the top for comparison.

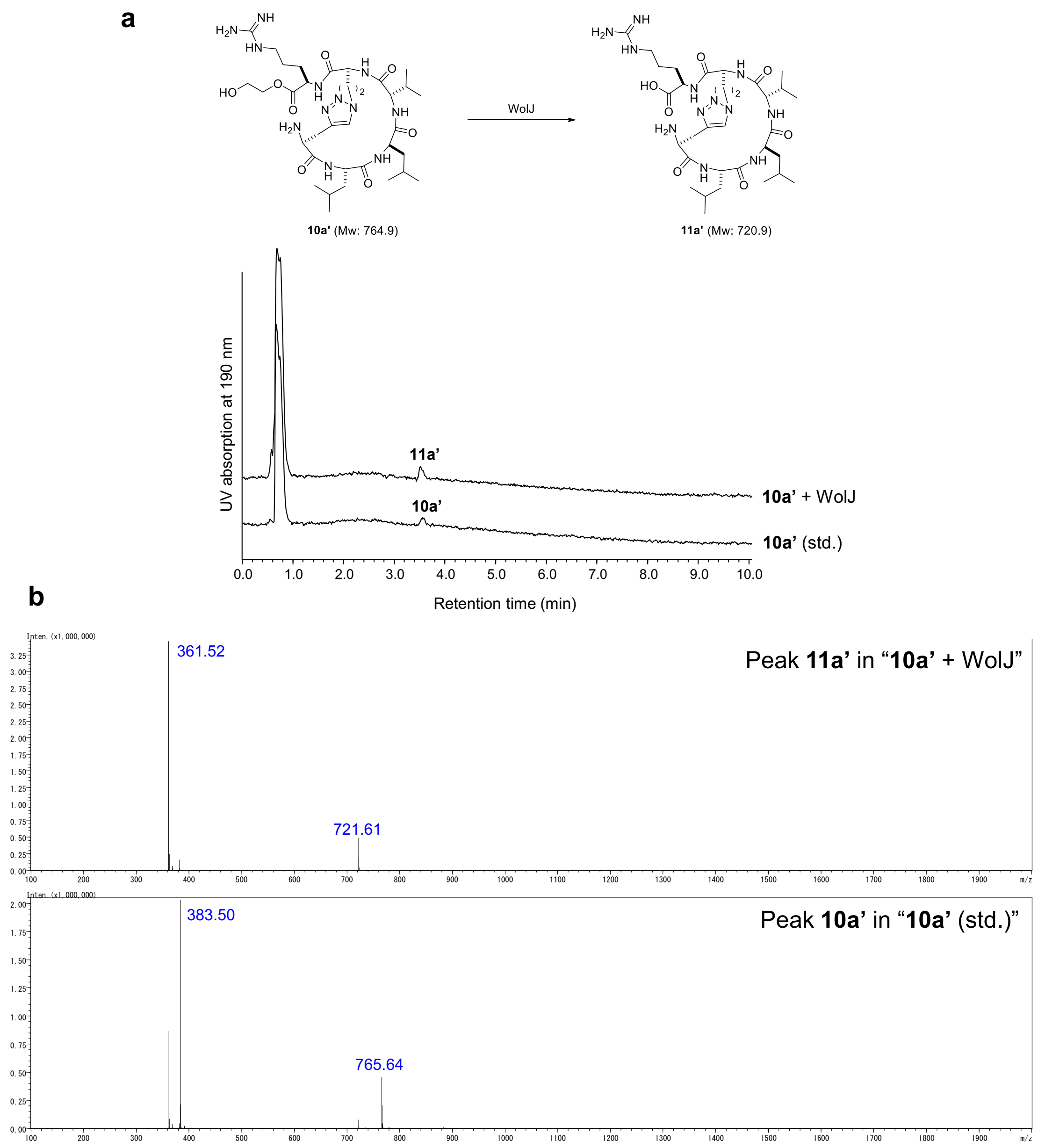

Figure S5. WolJ-reaction on CuAAC-cyclized peptide **10a'**. **10a'** was quantitatively hydrolyzed to give monocyclic peptide **11a'** by WolJ. (a) HPLC analysis of the WolJ reaction mixture with **10a'** as a substrate. (b) MS chromatograms of the peaks corresponding to **10a'** (bottom) and **11a'** (top).

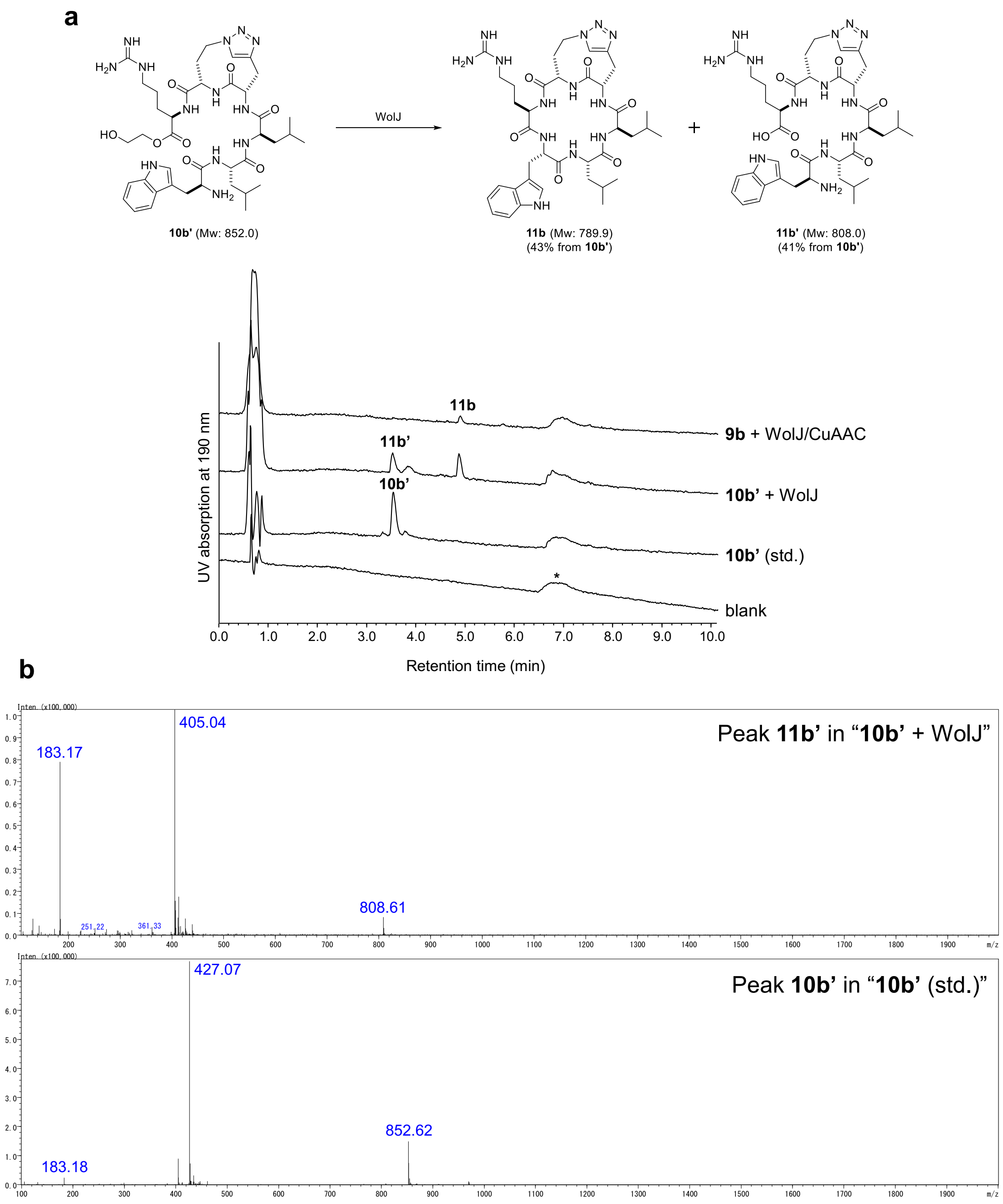

Figure S6. WolJ-reaction on CuAAC-cyclized peptide **10b'**. *: solvent peak. Chromatograms of blank injection and a reaction mixture of chemoenzymatic tandem cyclization (i.e., 1st WolJ, 2nd CuAAC) on the linear peptide **9b** were shown on the bottom and top for comparison, respectively. **10b'** was converted to bicyclic peptide **11b** in 43% conversion. Hydrolyzed mono-cyclic peptide **11b'** was also generated in 41% conversion. (a) HPLC analysis of the WolJ reaction mixture with **10b'** as a substrate. (b) MS chromatograms of the peaks corresponding to **10b'** (bottom) and **11b'** (top).

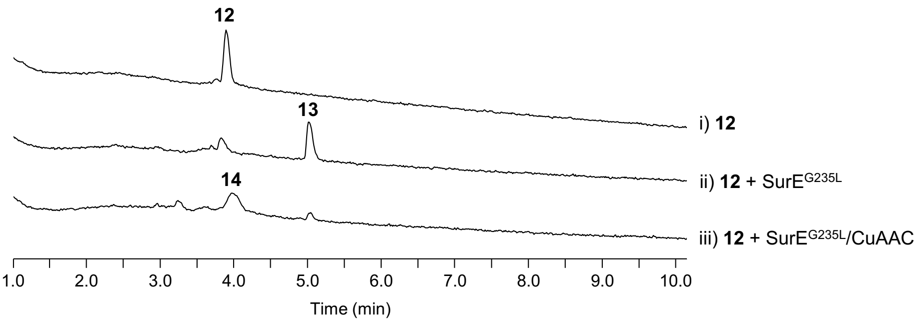

Figure S7. Chemoenzymatic tandem cyclization of BC1 sequences **12**. Samples were analyzed by HPLC. Column elutes were monitored with UV absorption at 190 nm. Top: **12** without SurE^{G235L}, middle: **12** with SurE^{G235L}, bottom: **12**+SurE^{G235L} reaction mixture treated with CuAAC.

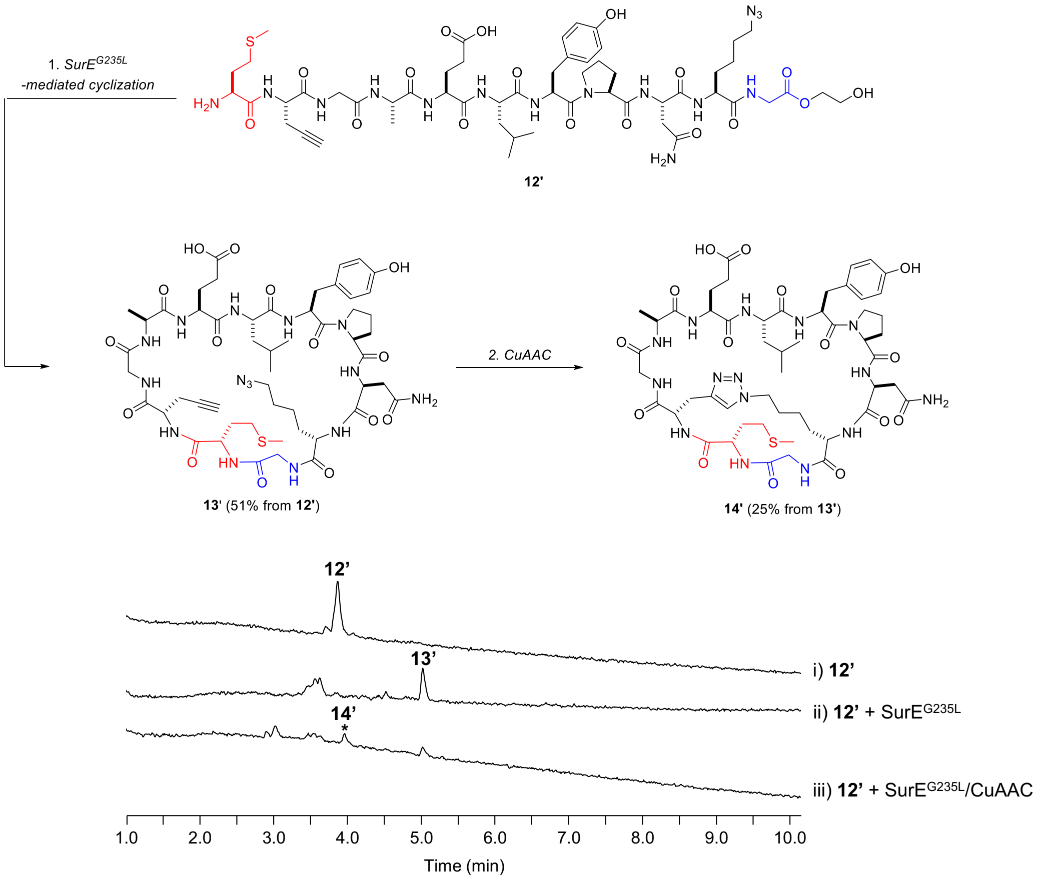

Figure S8. Chemoenzymatic tandem cyclization of BC1 sequences **12’**. Samples were analyzed by HPLC. Column elutes were monitored with UV absorption at 190 nm. Top: **12’** without SurE^{G235L}, middle: **12’** with SurE^{G235L}, bottom: **12’**+SurE^{G235L} reaction mixture treated with CuAAC.

References

1. Kobayashi, M., Fujita, K., Matsuda, K. & Wakimoto, T. Streamlined Chemoenzymatic Synthesis of Cyclic Peptides by Non-ribosomal Peptide Cyclases. *J. Am. Chem. Soc.* **145**, 3270−3275 (2023).
2. Kuranaga, T. *et al.* Total synthesis of the nonribosomal peptide surugamide B and identification of a new offloading cyclase family. *Angew. Chem., Int. Ed.* **57**, 9447−9451 (2018).
