# Supplementary material for: Chemoenzymatic tandem cyclization for the facile synthesis of bicyclic peptides
Source: Commun Chem. 2024 Mar 28;7:67. doi: 10.1038/s42004-024-01147-w (PMC10978974; doi:10.1038/s42004-024-01147-w)
Supplement: Supplementary file 2 — Description of Additional Supplementary Files [file 42004_2024_1147_MOESM2_ESM.pdf]

Description of Additional Supplementary Files

File name: Supplementary Data 1

Description: MS spectra

File name: Supplementary Data 2

Description: NMR spectra
