# Supplementary material for: Chemoenzymatic tandem cyclization for the facile synthesis of bicyclic peptides
Source: Commun Chem. 2024 Mar 28;7:67. doi: 10.1038/s42004-024-01147-w (PMC10978974; doi:10.1038/s42004-024-01147-w)
Supplement: Supplementary file 3 — Supplementary Data 1 [file 42004_2024_1147_MOESM3_ESM.pdf]

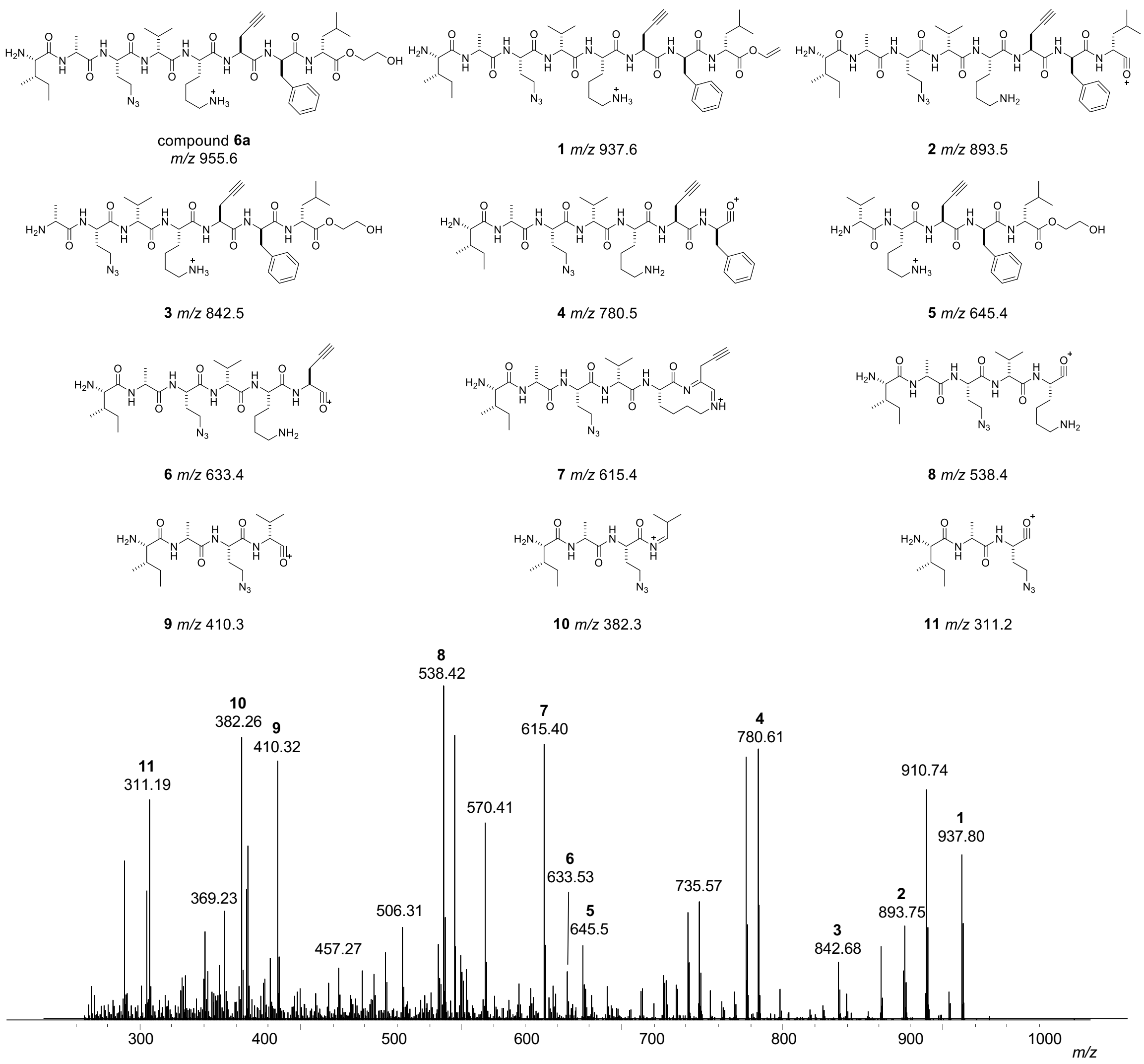

Figure S9. MS2 spectrum of **6a**

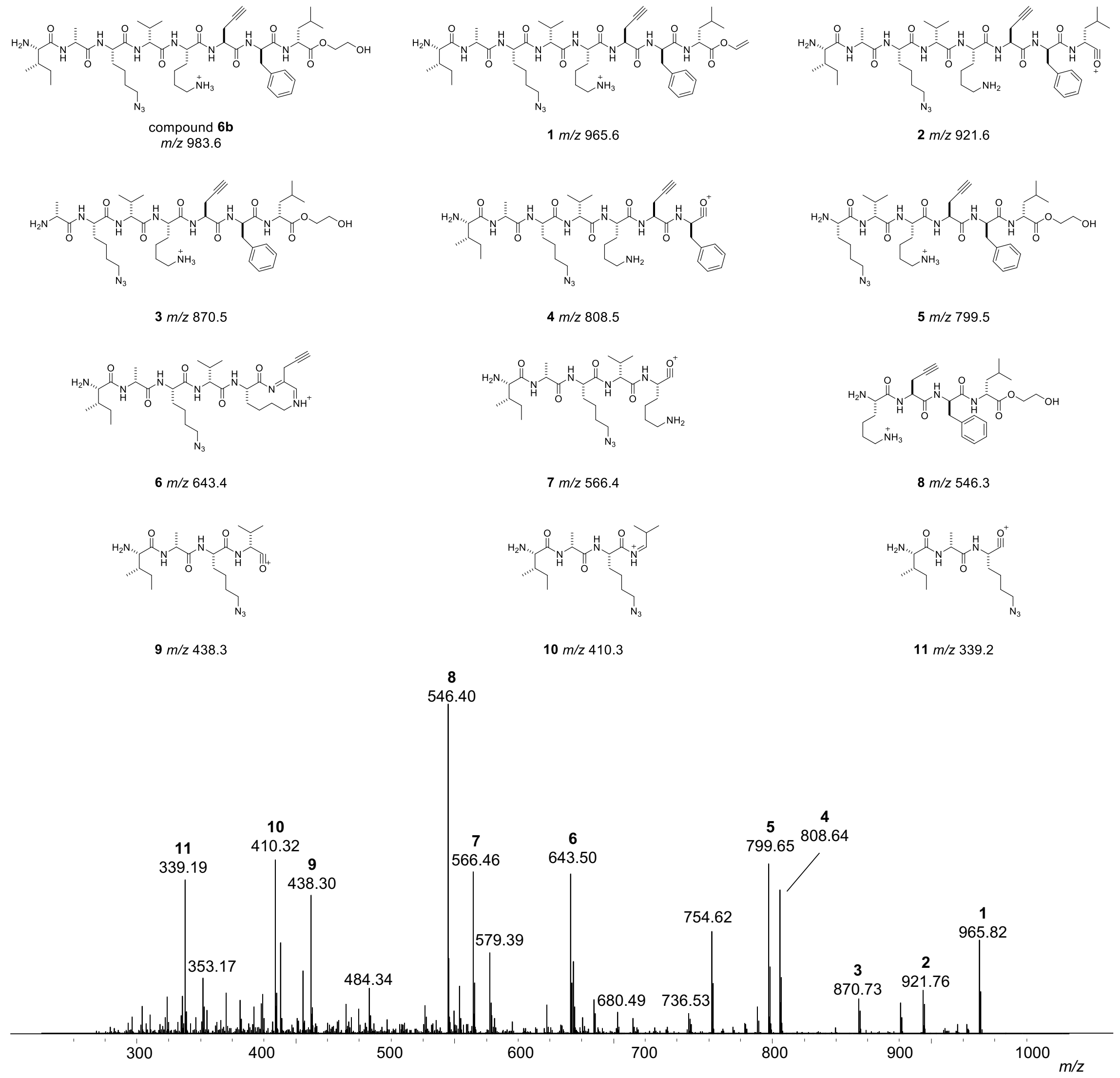

Figure S10. MS2 spectrum of **6b**

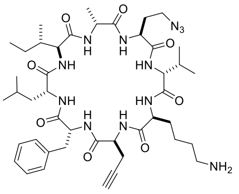

compound **7a**
m/z 893.5

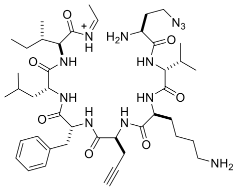

1 *m/z* 865.5

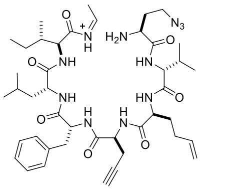

2 *m/z* 848.5

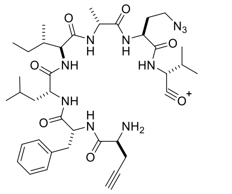

3 *m/z* 765.4

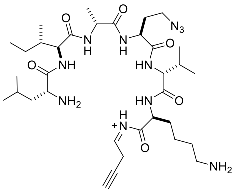

4 *m/z* 718.5

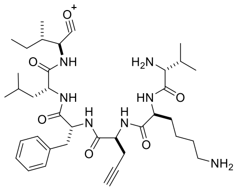

5 *m/z* 696.4

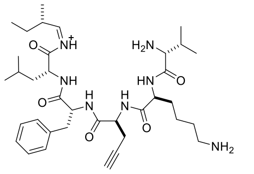

6 *m/z* 668.5

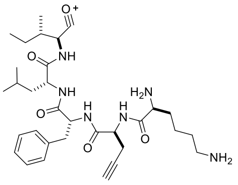

7 *m/z* 597.4

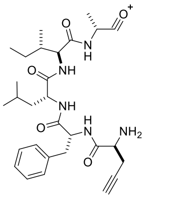

8 *m/z* 540.3

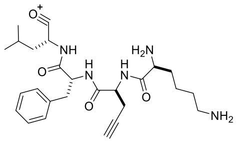

9 *m/z* 484.3

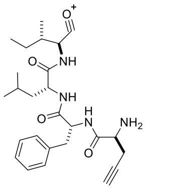

10 *m/z* 469.3

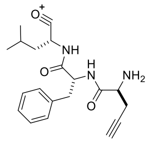

11 *m/z* 356.2

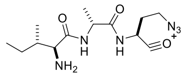

12 *m/z* 311.2

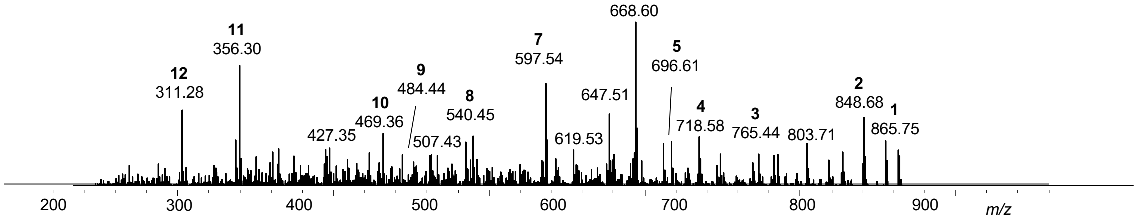

Figure S11. MS2 spectrum of **7a**

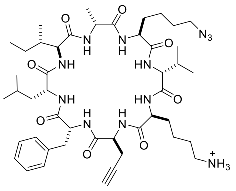

compound **7b**
m/z 921.6

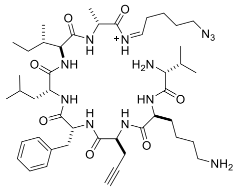

1 *m/z* 893.6

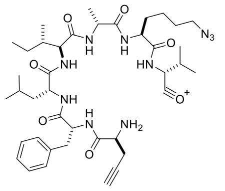

2 *m/z* 793.5

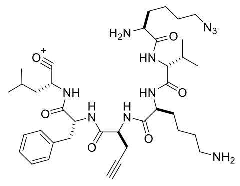

3 *m/z* 737.5

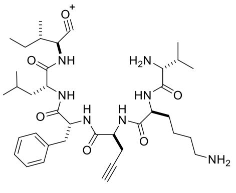

4 *m/z* 696.4

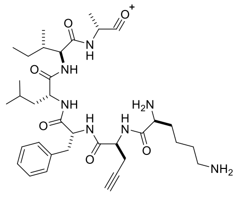

5 *m/z* 668.4

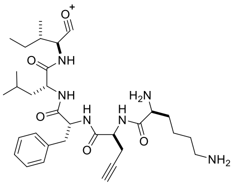

6 *m/z* 597.4

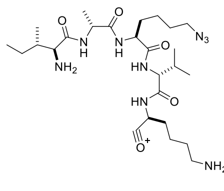

7 *m/z* 566.4

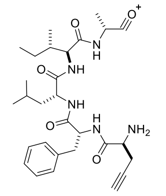

8 *m/z* 540.3

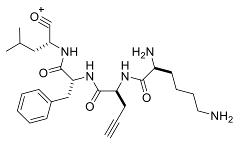

9 *m/z* 484.3

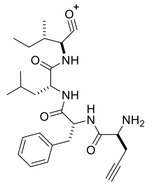

10 *m/z* 469.3

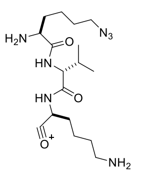

11 *m/z* 382.3

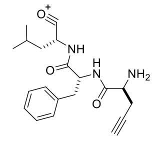

12 *m/z* 356.2

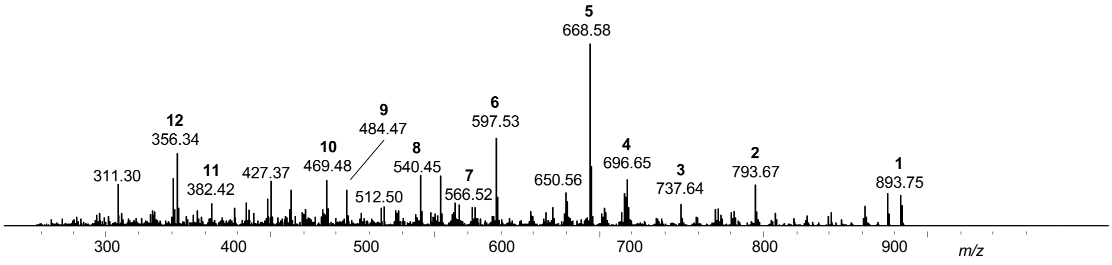

Figure S12. MS2 spectrum of **7b**

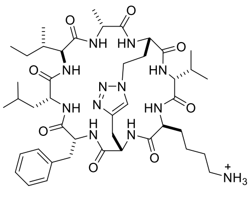

compound **8a**
m/z 893.5

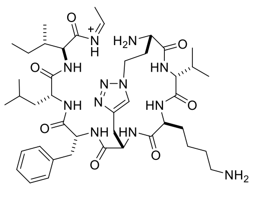

1 *m/z* 865.5

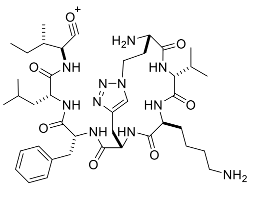

2 *m/z* 822.5

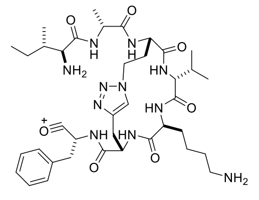

3 *m/z* 780.5

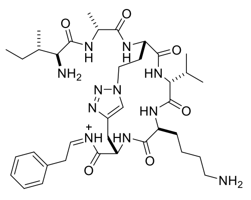

4 *m/z* 752.5

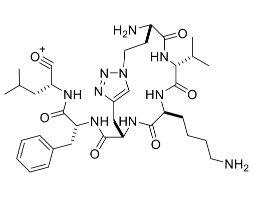

5 *m/z* 709.4

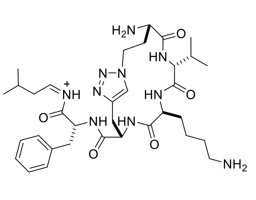

6 *m/z* 681.4

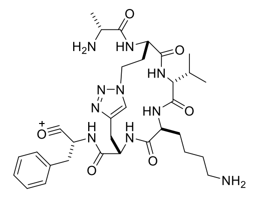

7 *m/z* 667.4

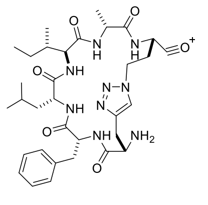

8 *m/z* 666.4

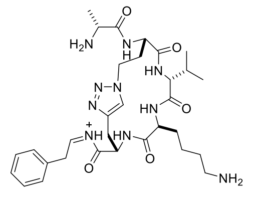

9 *m/z* 639.4

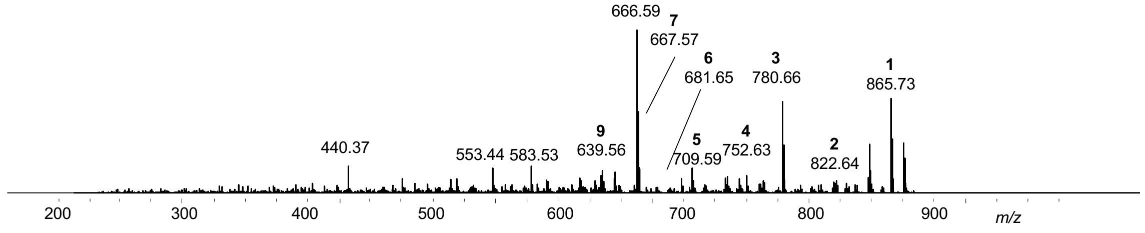

Figure S13. MS2 spectrum of **8a**

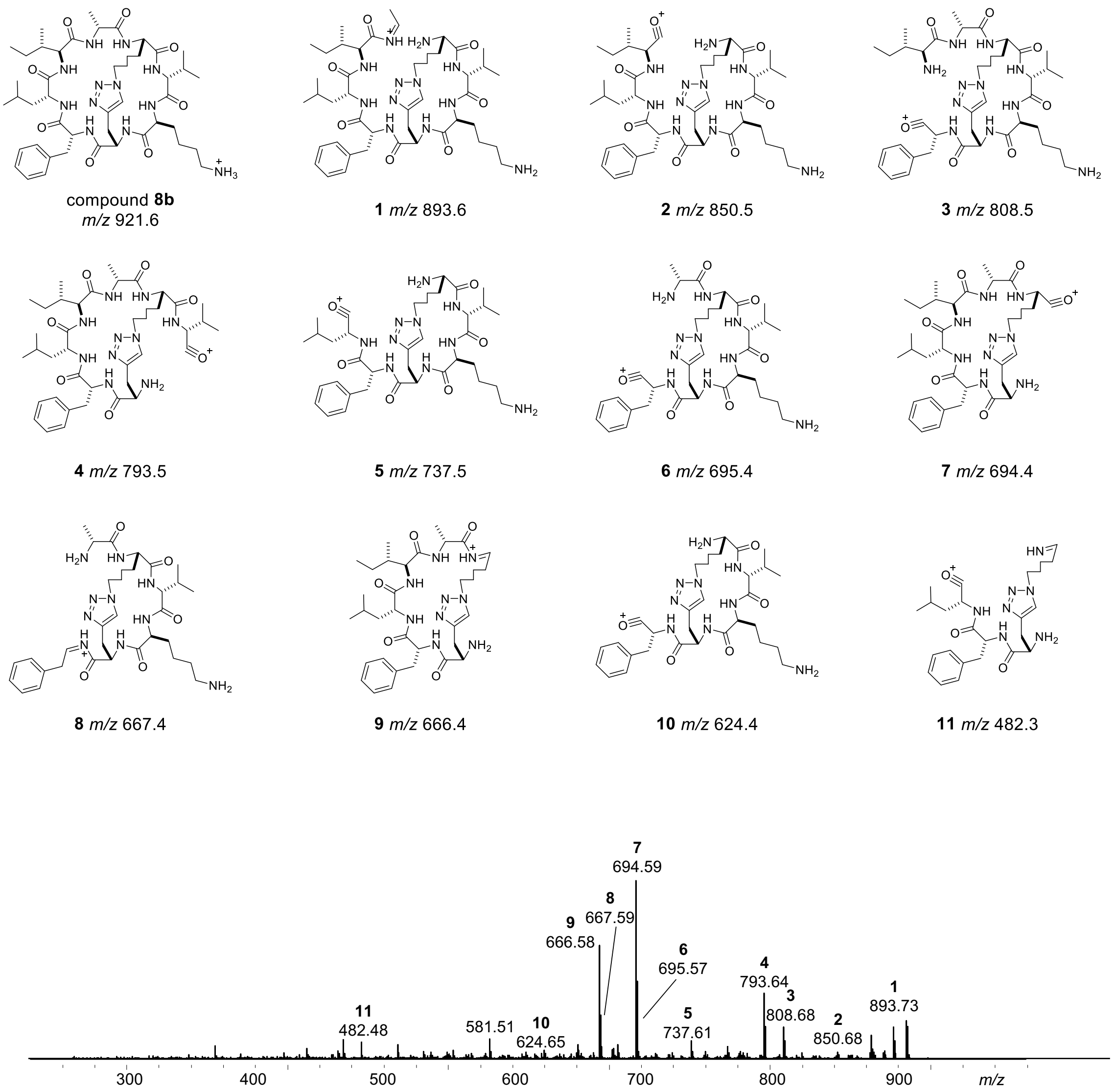

Figure S14. MS2 spectrum of **8b**

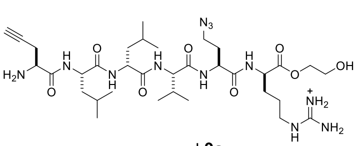

compound **9a**
m/z 765.5

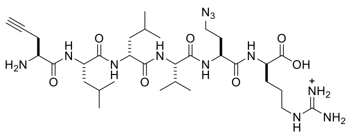

1 *m/z* 721.4

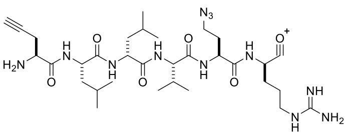

2 *m/z* 703.4

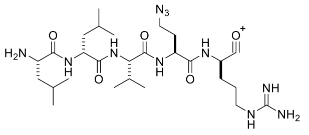

3 *m/z* 608.4

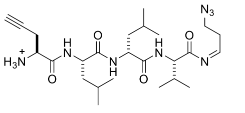

4 *m/z* 519.3

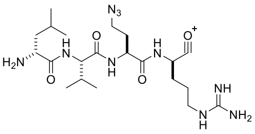

5 *m/z* 495.3

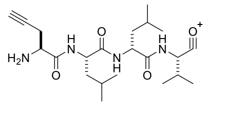

6 *m/z* 421.3

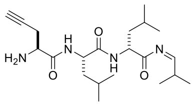

7 *m/z* 393.3

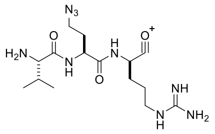

8 *m/z* 382.2

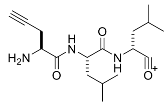

9 *m/z* 322.2

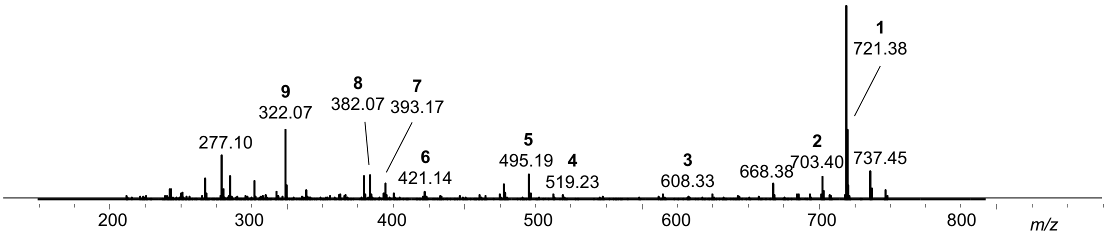

Figure S15. MS2 spectrum of **9a**

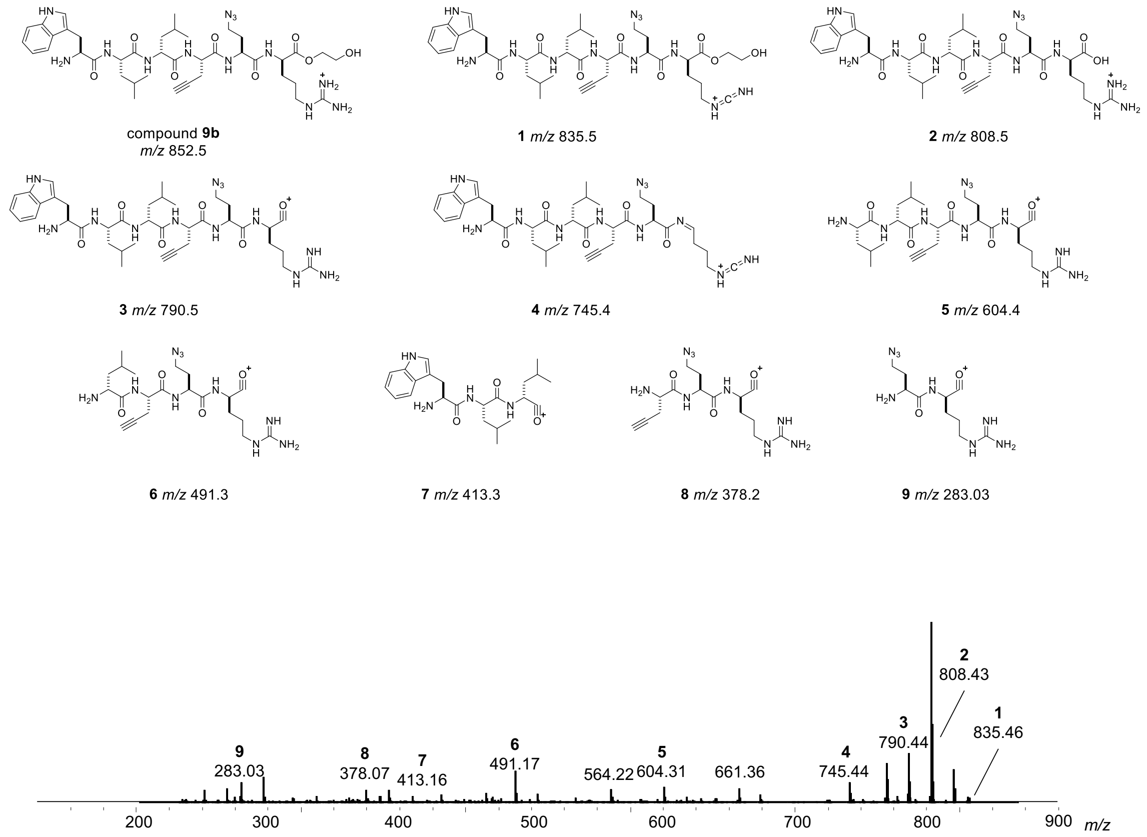

Figure S16. MS2 spectrum of **9b**

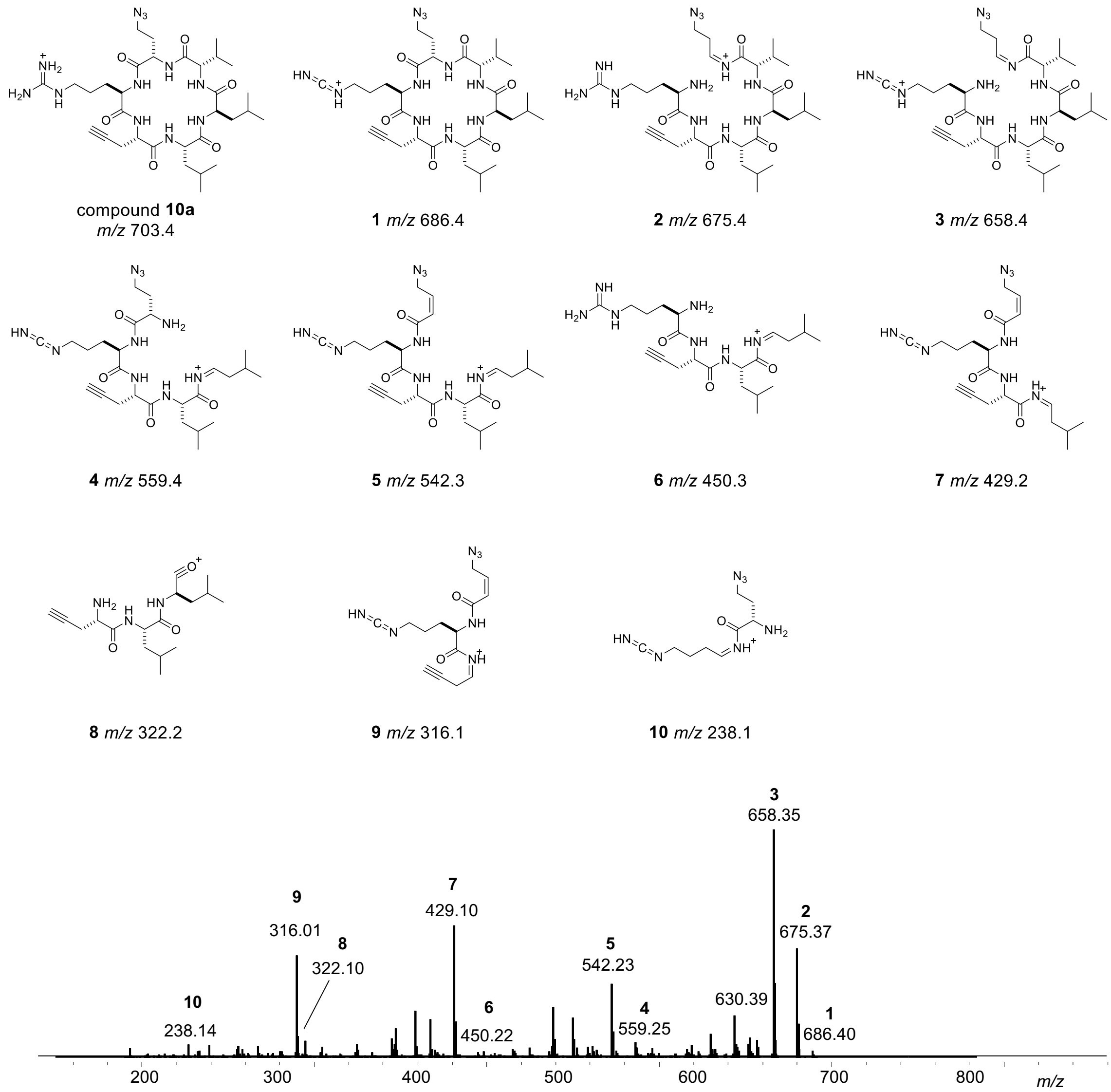

Figure S17. MS2 spectrum of **10a**

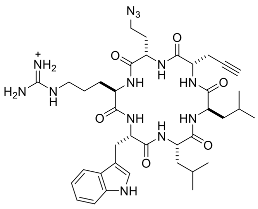

compound **10b**
m/z 790.5

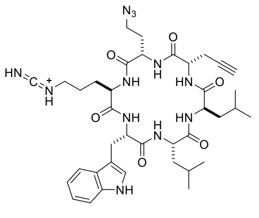

1 *m/z* 773.4

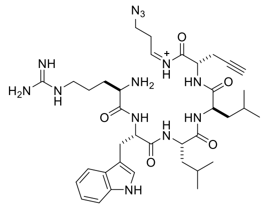

2 *m/z* 762.5

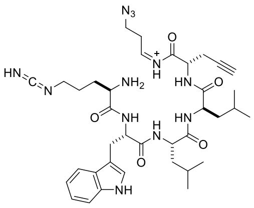

3 *m/z* 745.4

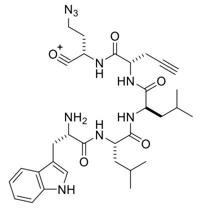

4 *m/z* 634.4

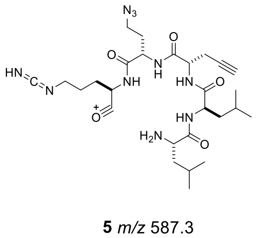

5 *m/z* 587.3

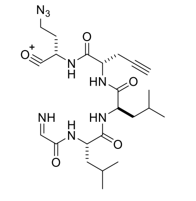

6 *m/z* 503.3

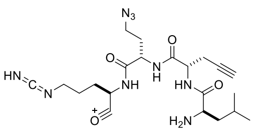

7 *m/z* 474.3

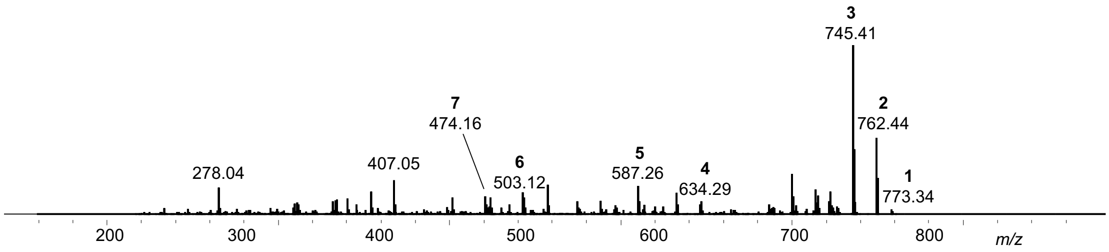

Figure S18. MS2 spectrum of **10b**

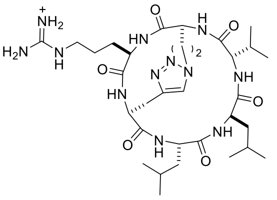

compound **11a**
m/z 703.4

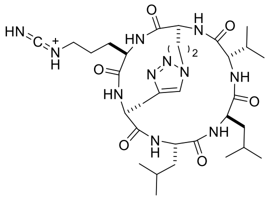

1 *m/z* 686.4

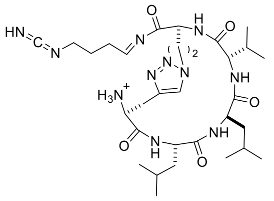

2 *m/z* 658.4

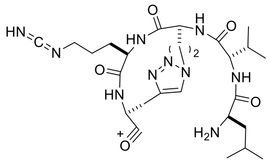

3 *m/z* 573.3

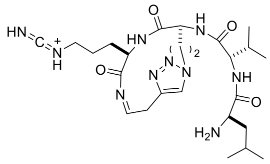

4 *m/z* 545.3

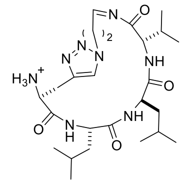

5 *m/z* 519.3

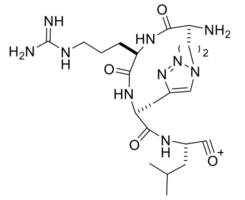

6 *m/z* 491.3

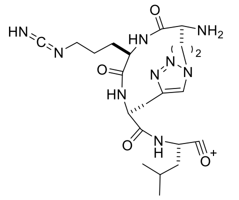

7 *m/z* 474.3

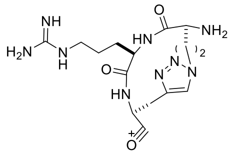

8 *m/z* 378.2

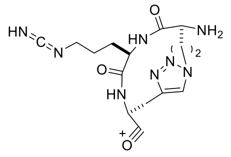

9 *m/z* 361.2

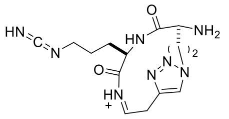

10 *m/z* 333.2

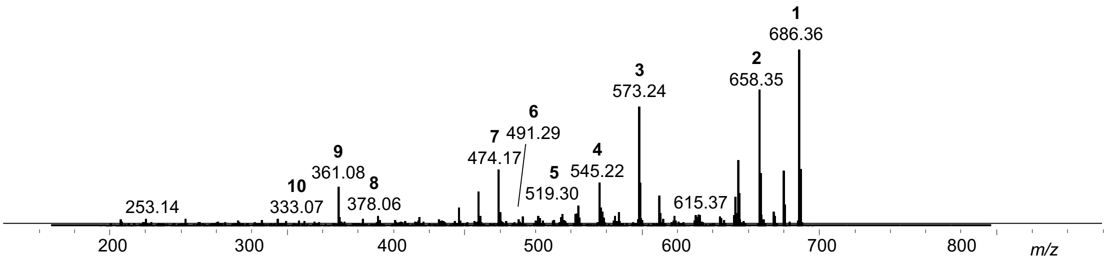

Figure S19. MS2 spectrum of **11a**

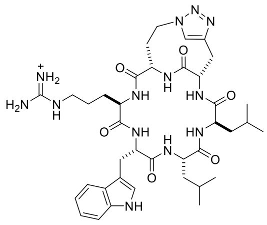

compound **11b**
m/z 790.5

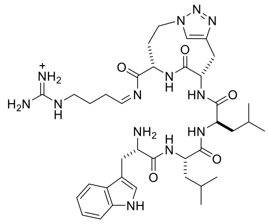

1 *m/z* 762.5

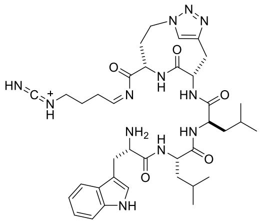

2 *m/z* 745.4

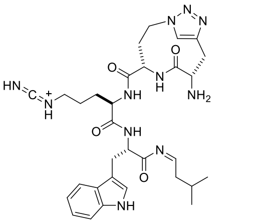

3 *m/z* 632.3

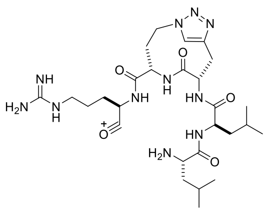

4 *m/z* 604.4

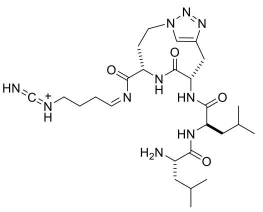

5 *m/z* 559.4

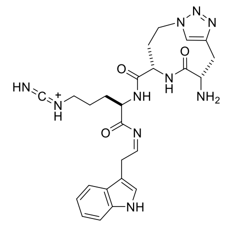

6 *m/z* 519.3

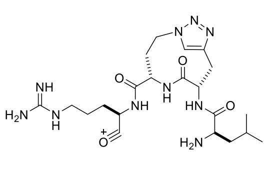

7 *m/z* 491.3

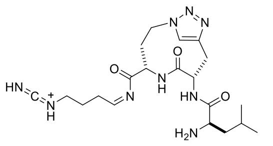

8 *m/z* 446.3

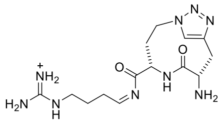

9 *m/z* 350.2

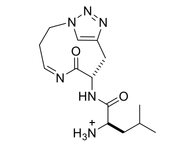

10 *m/z* 307.2

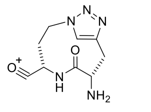

11 *m/z* 222.1

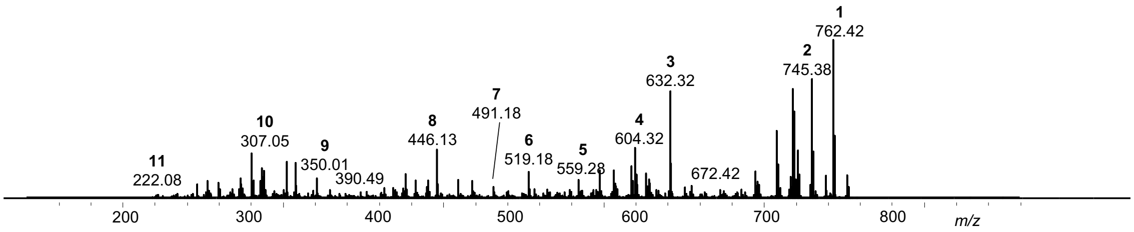

Figure S20. MS2 spectrum of **11b**

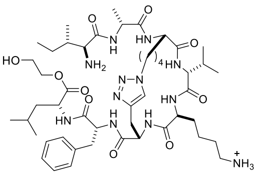

compound **7b'**
m/z 983.6

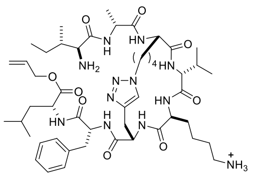

1 *m/z* 965.6

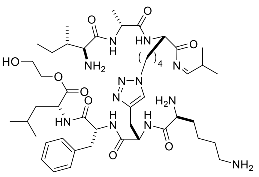

2 *m/z* 955.6

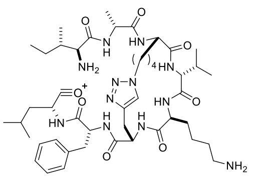

3 *m/z* 921.6

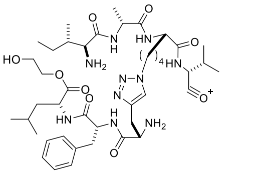

4 *m/z* 855.5

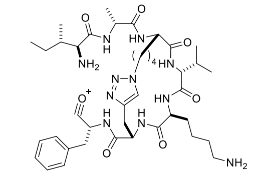

5 *m/z* 808.5

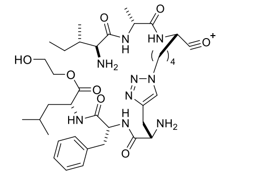

6 *m/z* 756.4

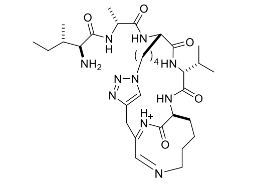

7 *m/z* 643.4

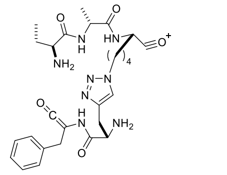

8 *m/z* 581.3

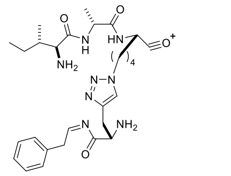

9 *m/z* 553.3

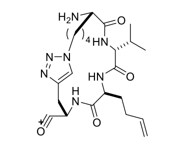

10 *m/z* 460.3

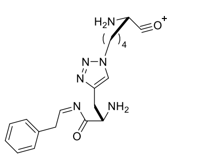

11 *m/z* 369.2

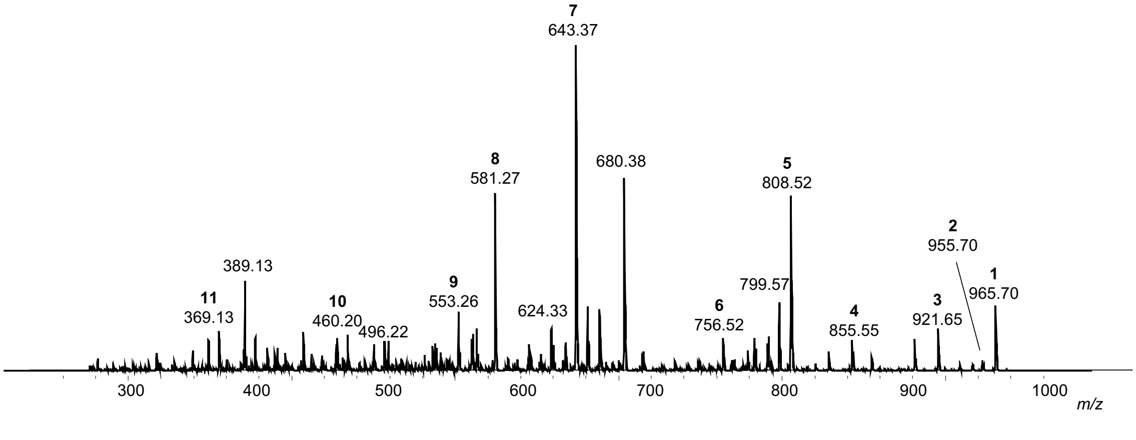

Figure S21. MS2 spectrum of **7b'**

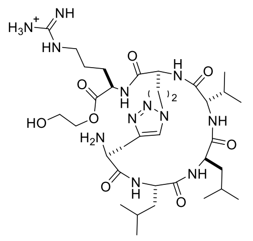

compound **10a'**
m/z 765.5

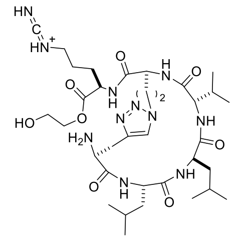

1 *m/z* 748.5

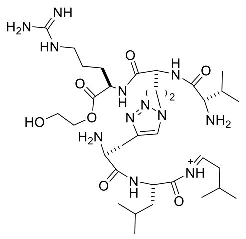

2 *m/z* 737.5

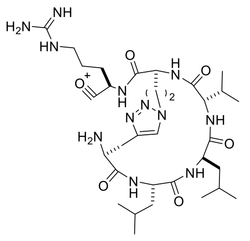

3 *m/z* 703.4

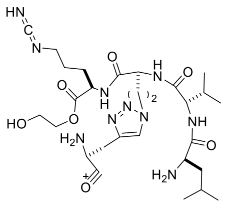

4 m/z 635.4

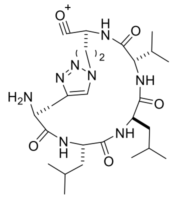

5 *m/z* 547.3

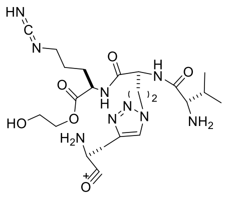

6 m/z 522.3

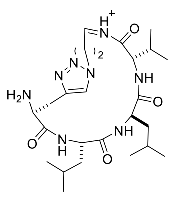

7 *m/z* 519.3

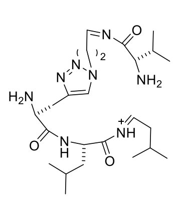

8 *m/z* 491.4

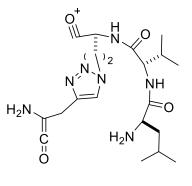

9 *m/z* 434.3

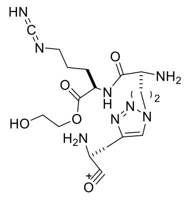

10 *m/z* 423.2

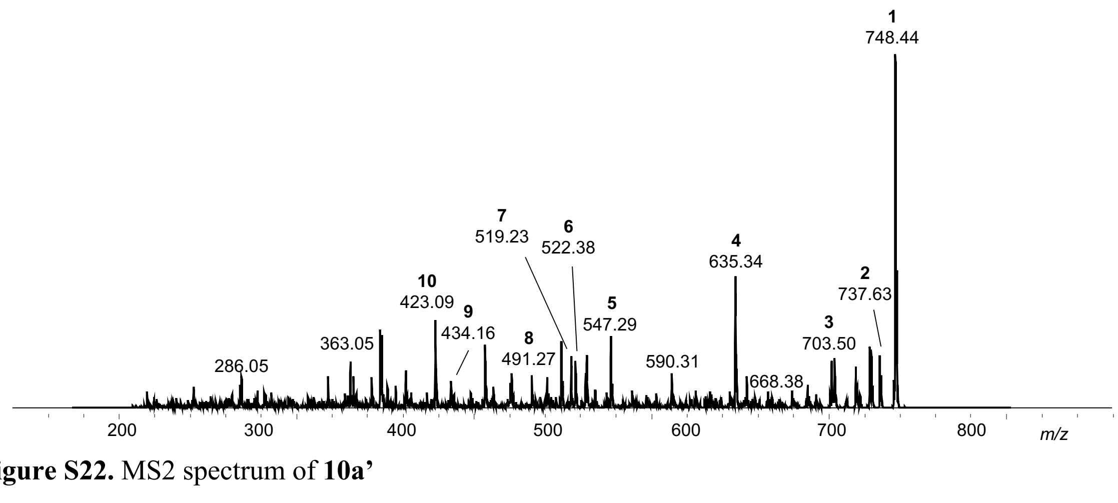

Figure S22. MS2 spectrum of **10a'**

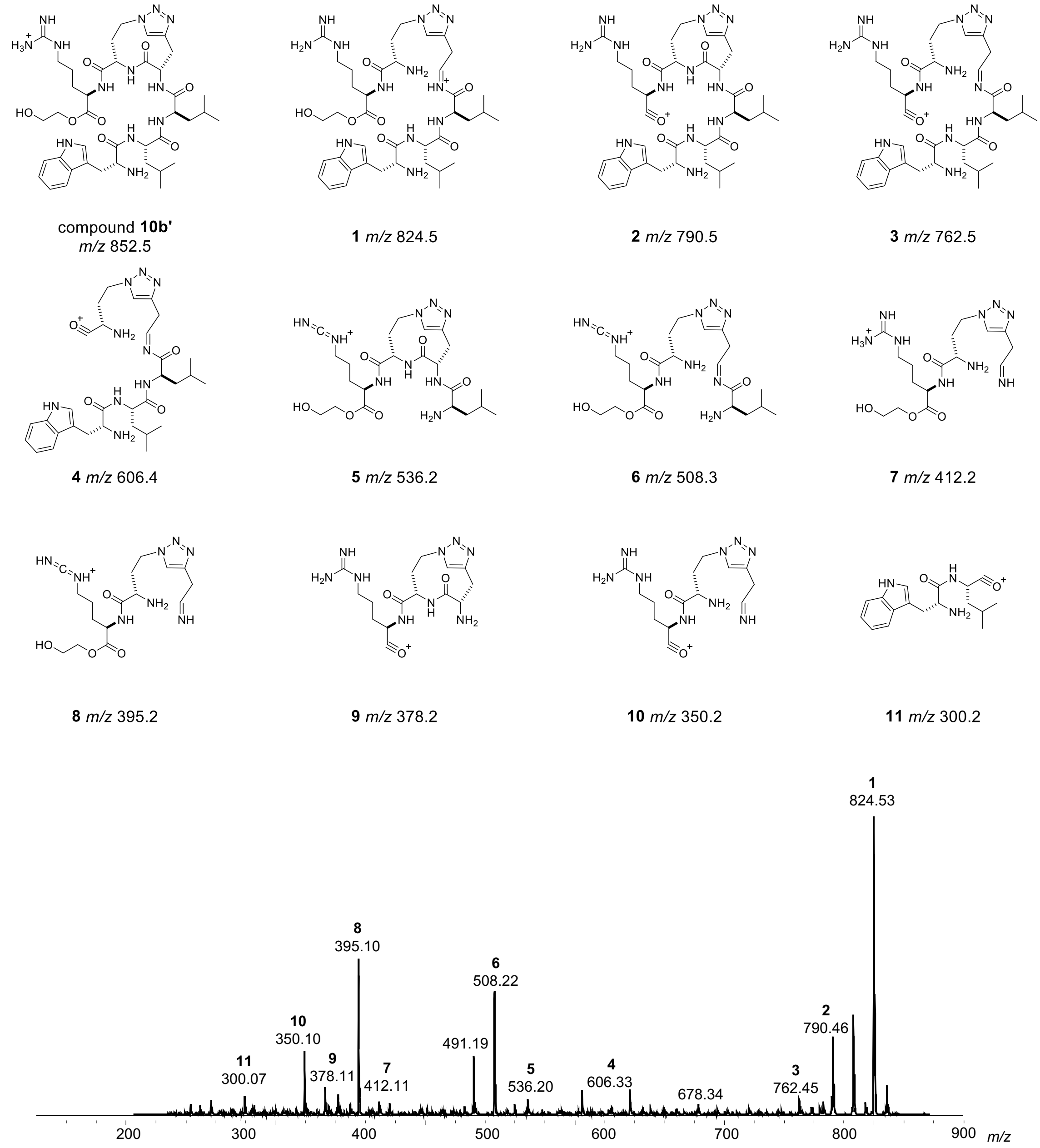

Figure S23. MS2 spectrum of **10b'**

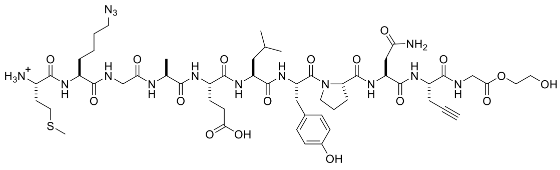

compound **12**
m/z 1244.6

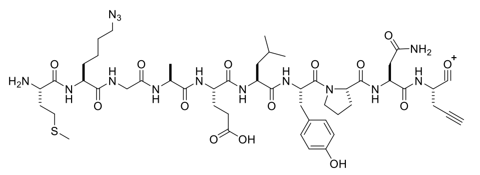

1 *m/z* 1125.5

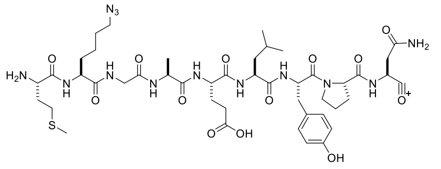

2 *m/z* 1030.5

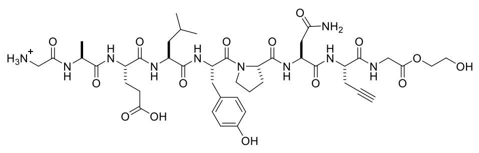

3 *m/z* 959.5

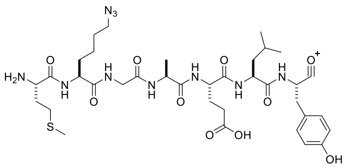

4 *m/z* 819.4

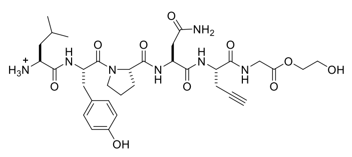

5 *m/z* 702.4

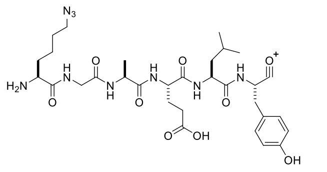

6 *m/z* 688.3

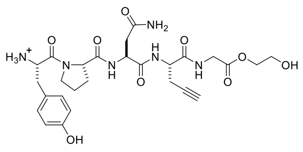

7 *m/z* 589.3

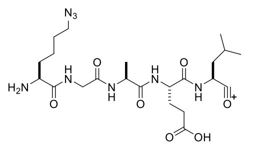

8 *m/z* 525.3

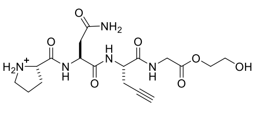

9 *m/z* 426.2

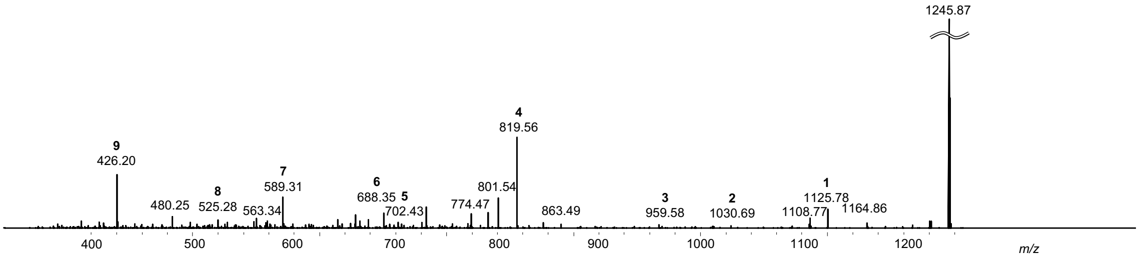

Figure S24. MS2 spectrum of **12**

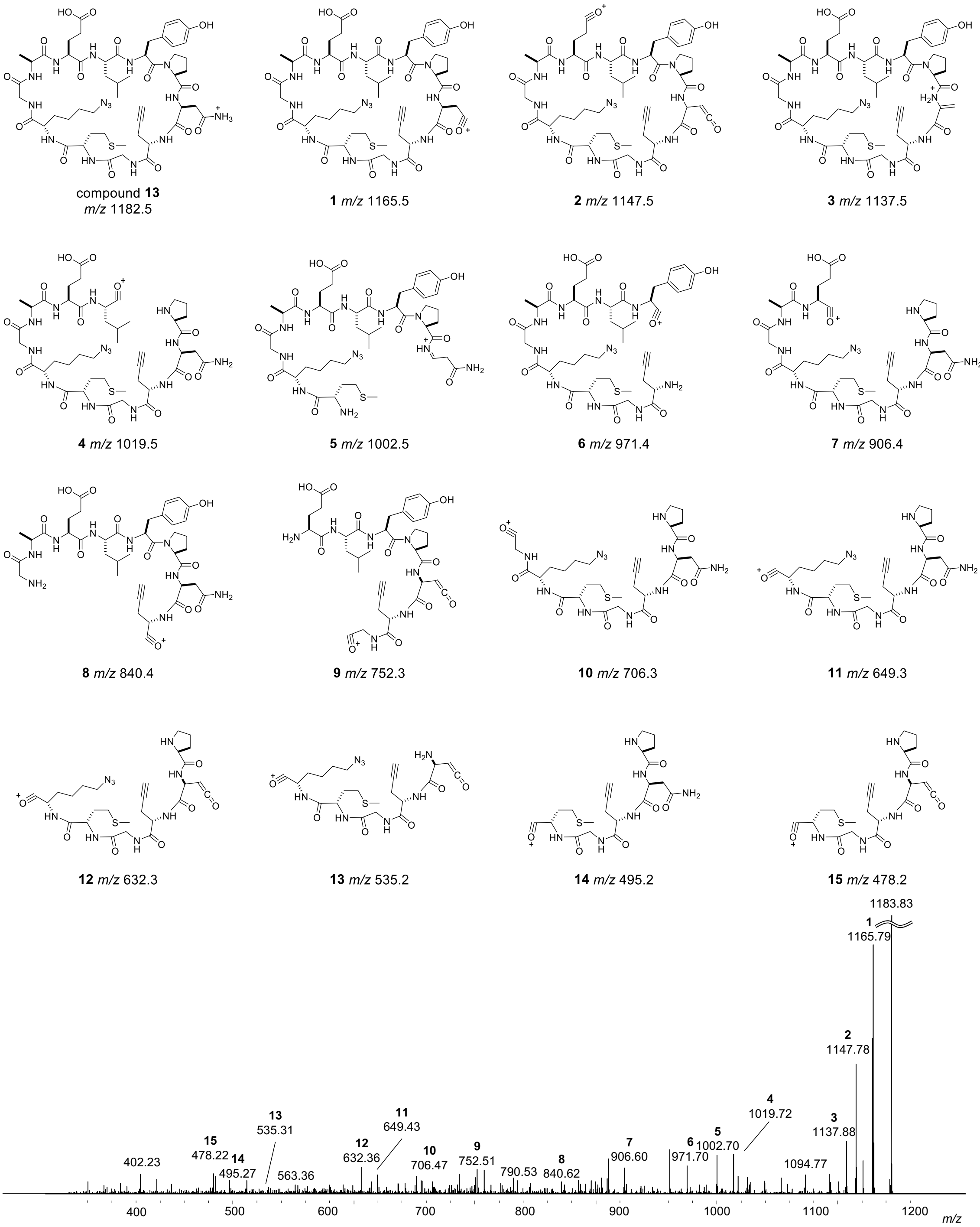

Figure S25. MS2 spectrum of **13**

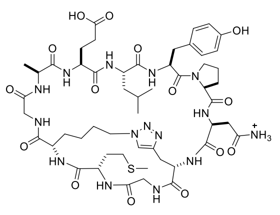

compound **14**
m/z 1182.5

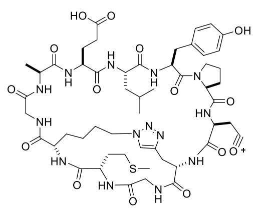

1 *m/z* 1165.5

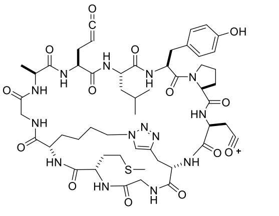

2 *m/z* 1147.8

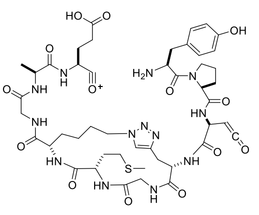

3 *m/z* 1052.4

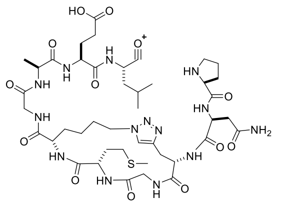

4 *m/z* 1019.5

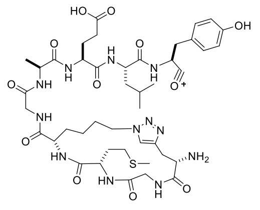

5 *m/z* 971.4

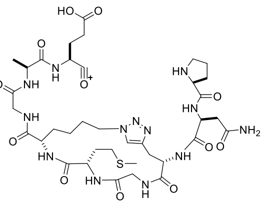

6 *m/z* 906.4

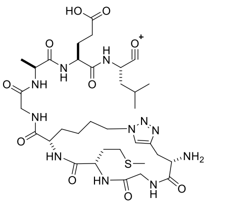

7 *m/z* 808.4

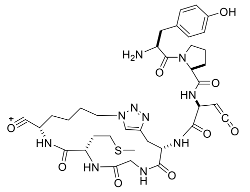

8 *m/z* 795.3

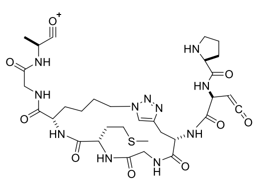

9 *m/z* 760.3

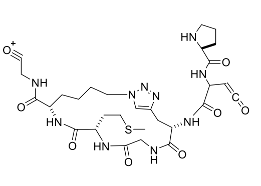

10 *m/z* 689.3

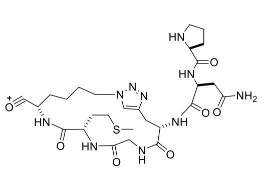

11 *m/z* 649.3

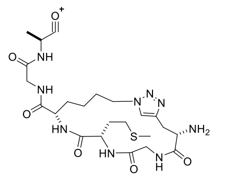

12 *m/z* 566.3

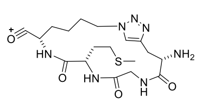

13 *m/z* 438.2

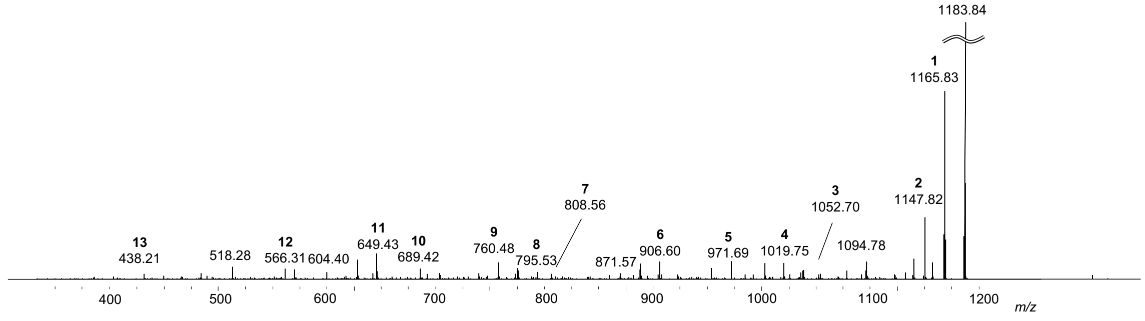

Figure S26. MS2 spectrum of **14**

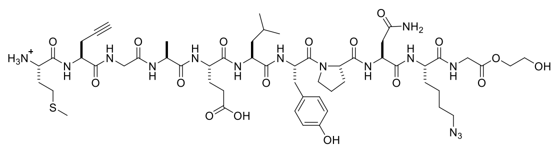

compound **12'**
m/z 1244.6

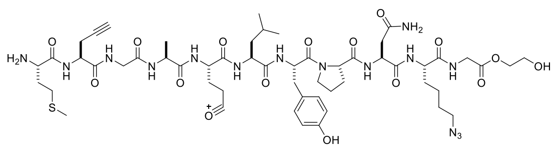

1 *m/z* 1226.6

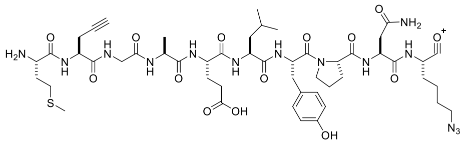

2 *m/z* 1125.5

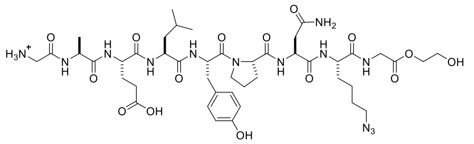

3 *m/z* 1018.5

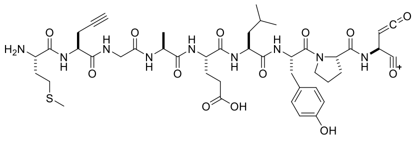

4 *m/z* 954.4

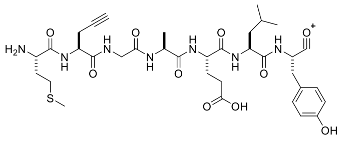

5 *m/z* 760.3

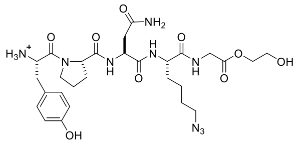

6 *m/z* 648.3

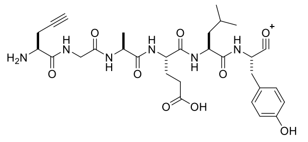

7 *m/z* 629.3

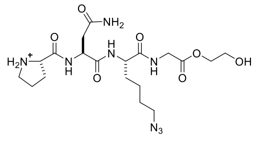

8 *m/z* 485.3

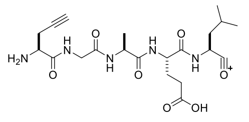

9 *m/z* 466.2

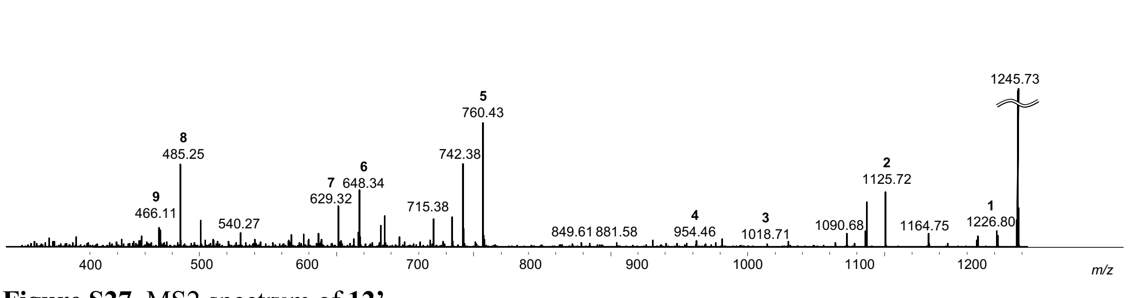

Figure S27. MS2 spectrum of **12’**

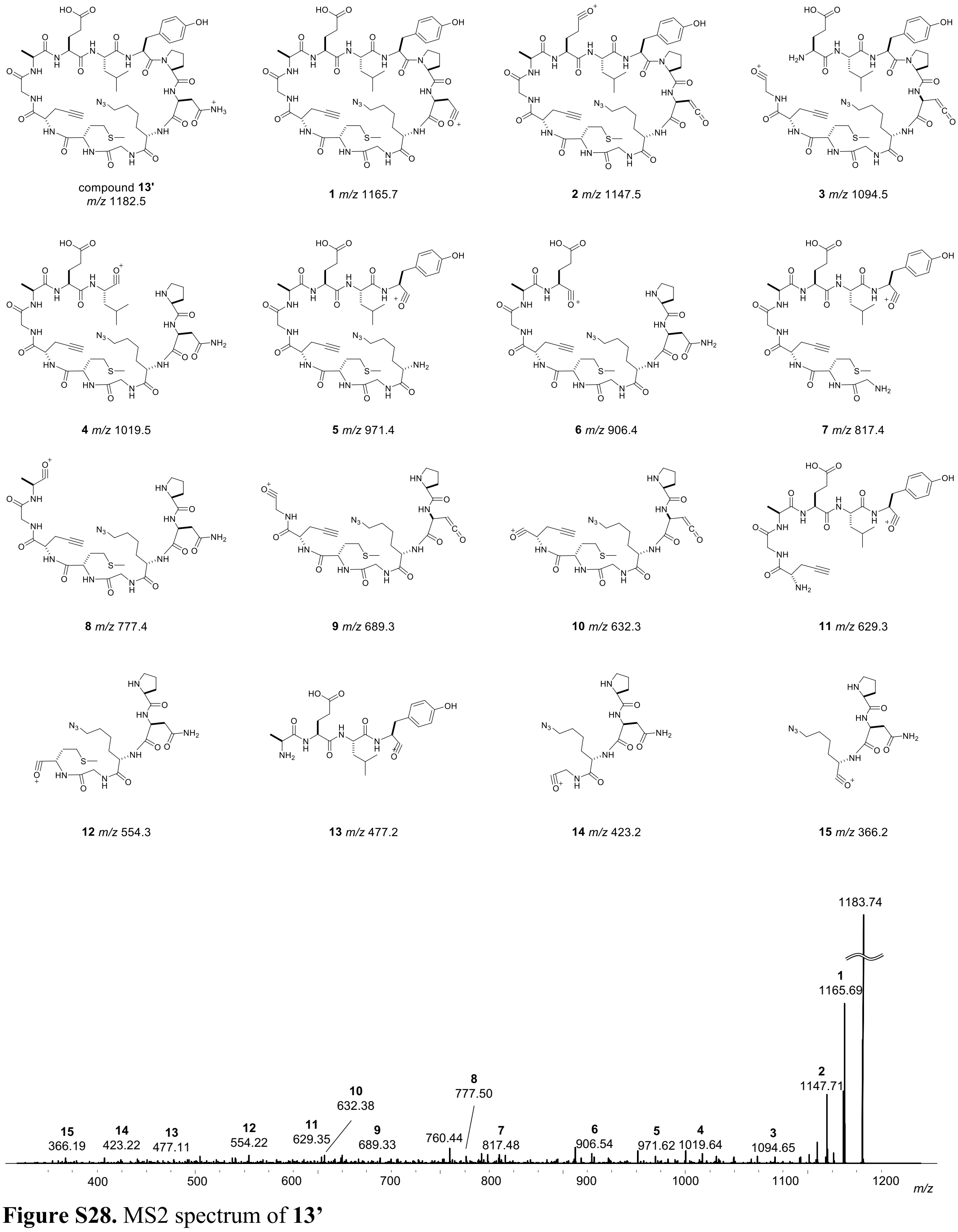

Figure S28. MS2 spectrum of **13’**

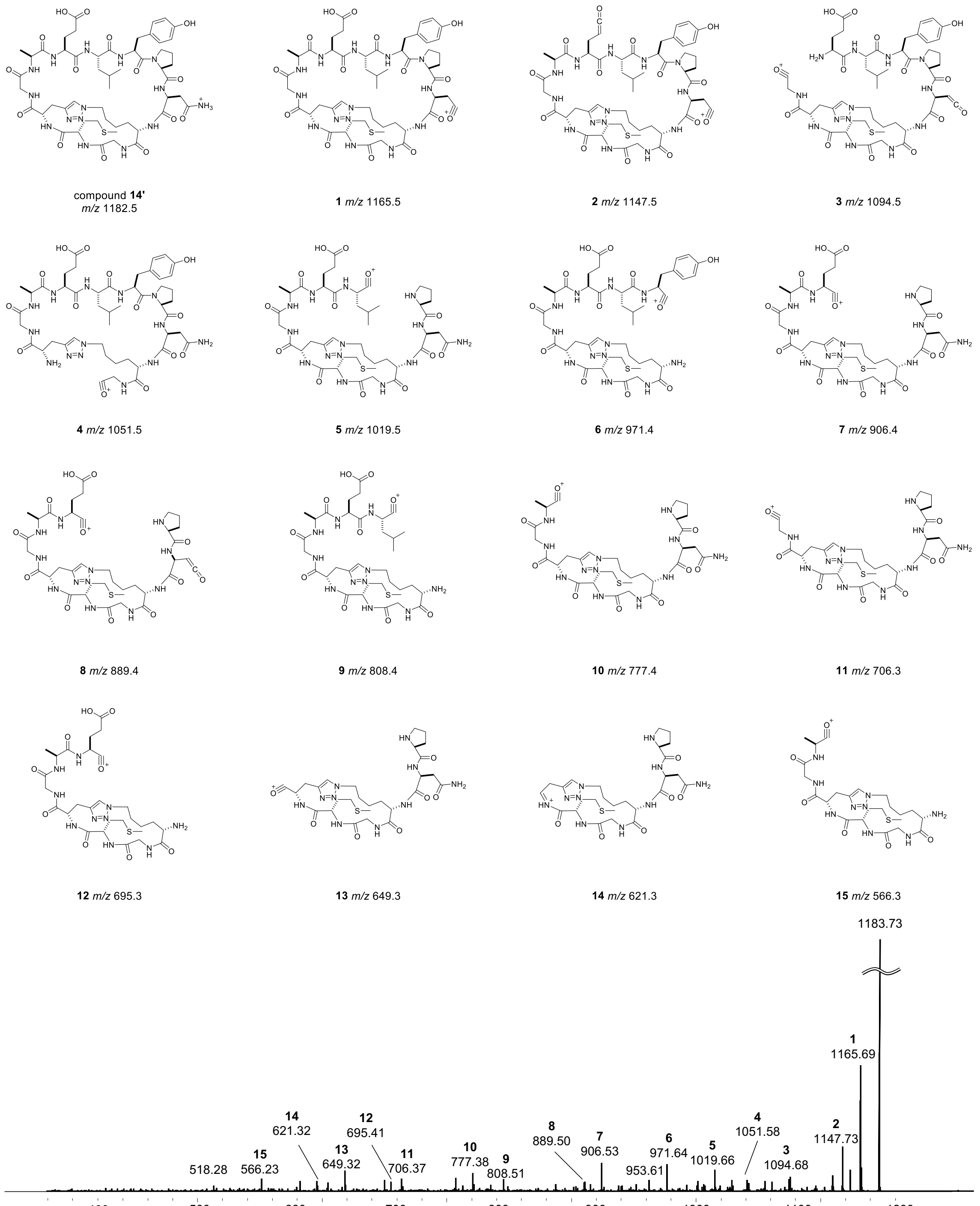

Figure S29. MS2 spectrum of **14'**
