# Supplementary material for: Chemoenzymatic tandem cyclization for the facile synthesis of bicyclic peptides
Source: Commun Chem. 2024 Mar 28;7:67. doi: 10.1038/s42004-024-01147-w (PMC10978974; doi:10.1038/s42004-024-01147-w)
Supplement: Supplementary file 4 — Supplementary Data 2 [file 42004_2024_1147_MOESM4_ESM.pdf]

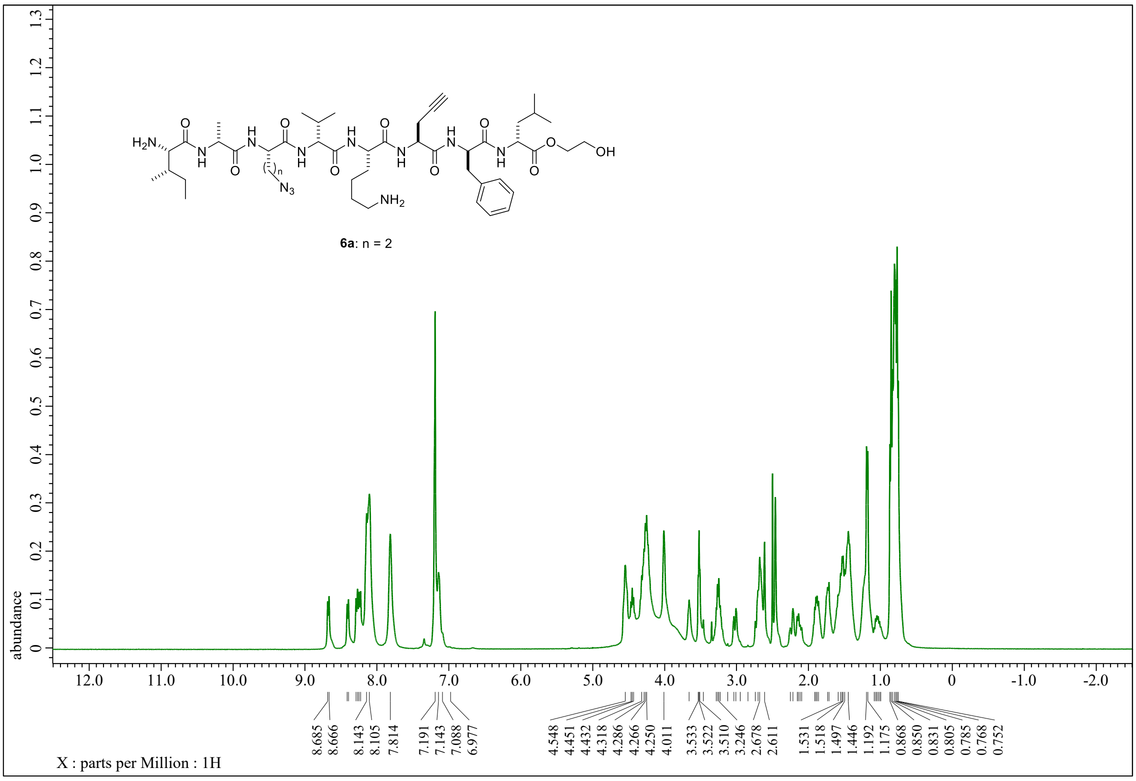

Figure S30. ^{1}H NMR spectrum of **6a** in DMSO-*d*$_6$ (400 MHz)

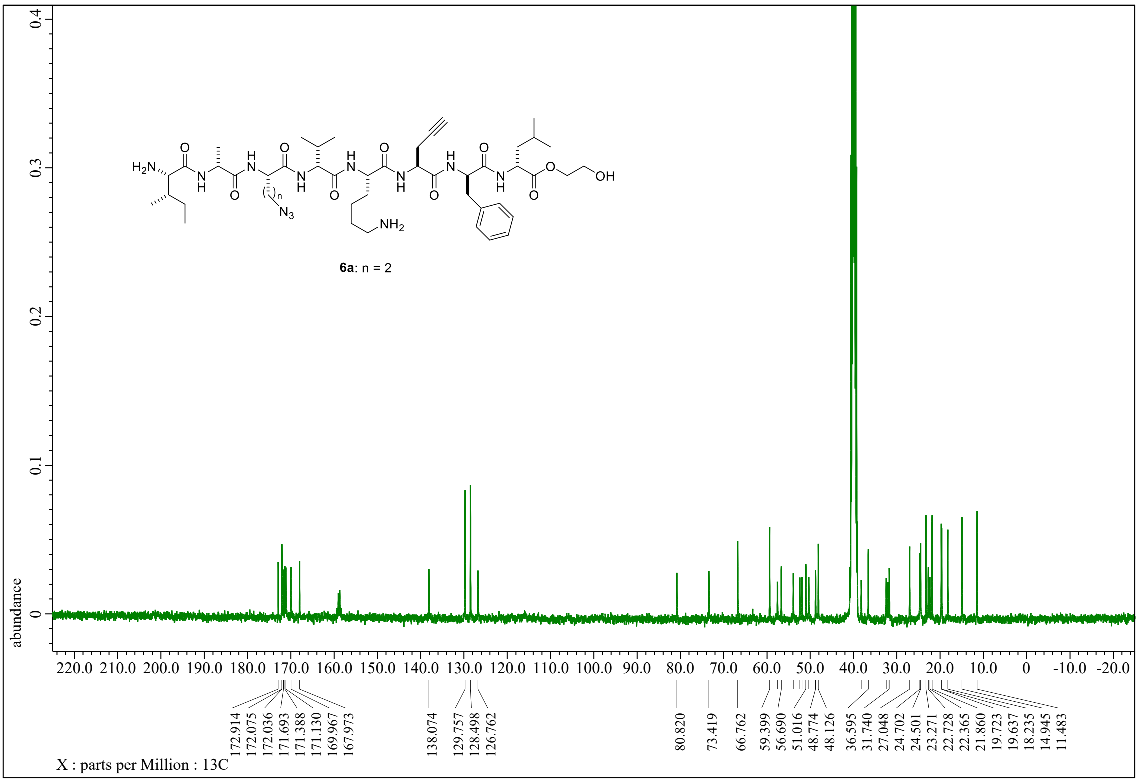

Figure S31. ^{13}C NMR spectrum of **6a** in DMSO-*d*$_6$ (100 MHz)

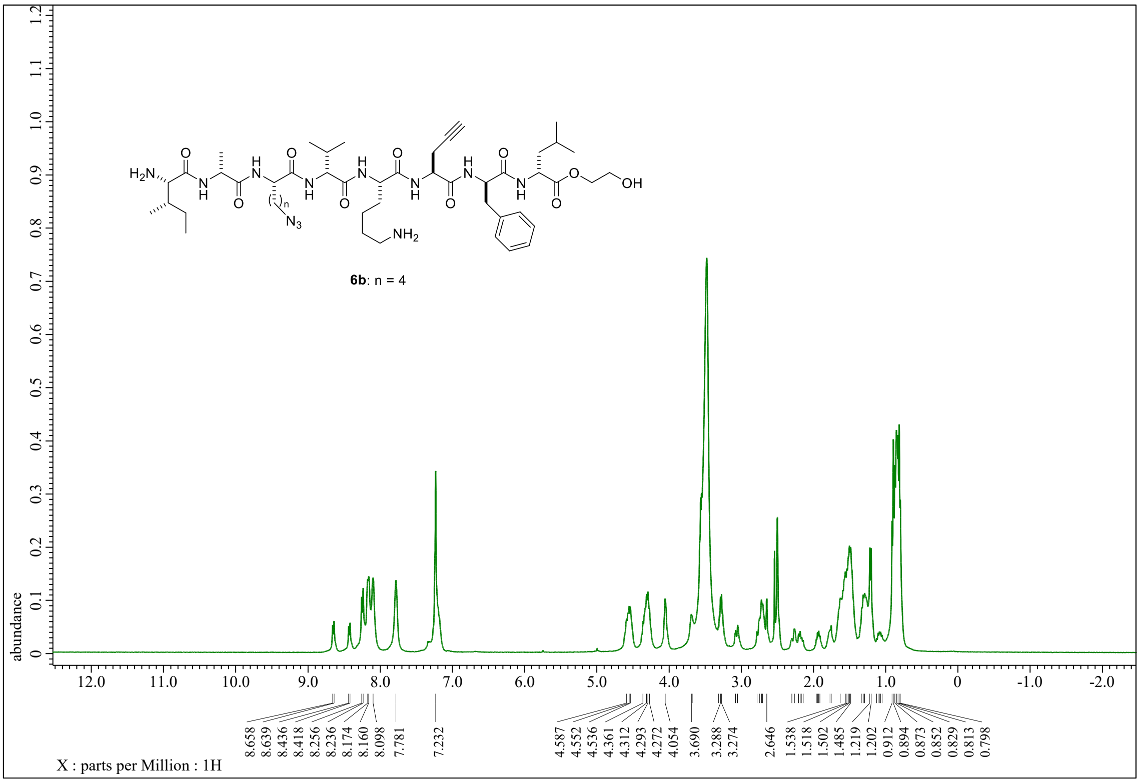

Figure S32. ^{1}H NMR spectrum of **6b** in DMSO-d_6 (400 MHz)

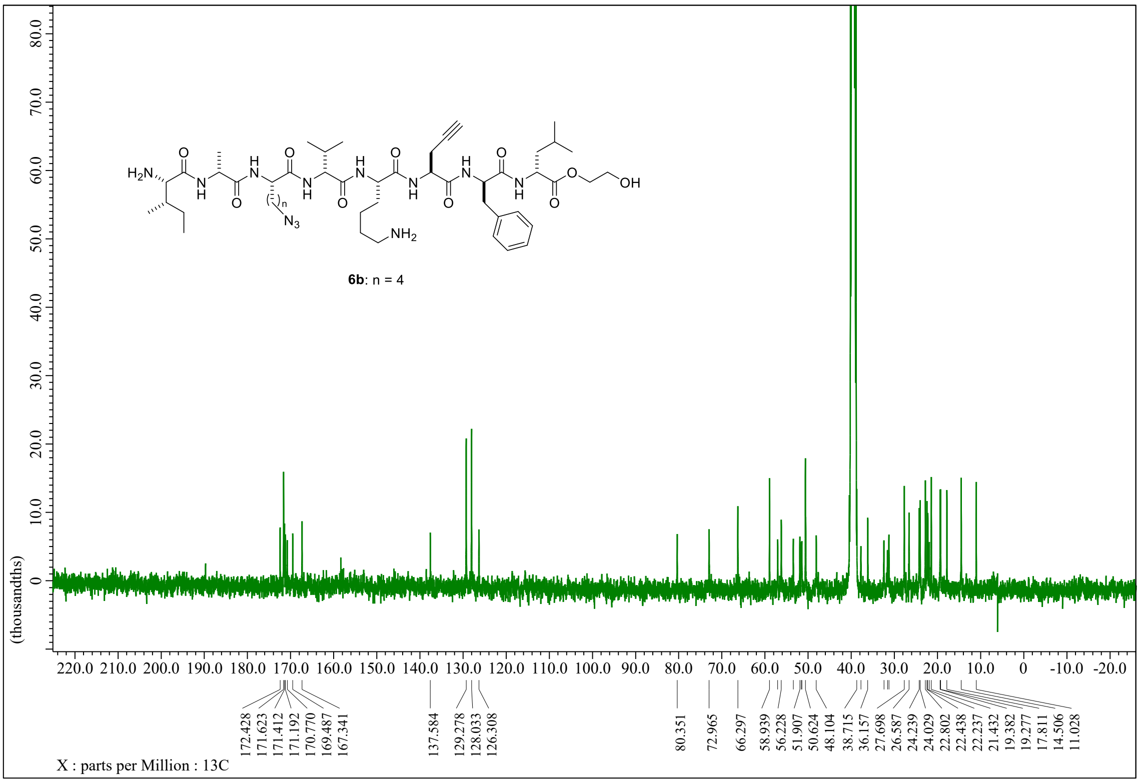

Figure S33. ^{13}C NMR spectrum of **6b** in DMSO-d_6 (100 MHz)

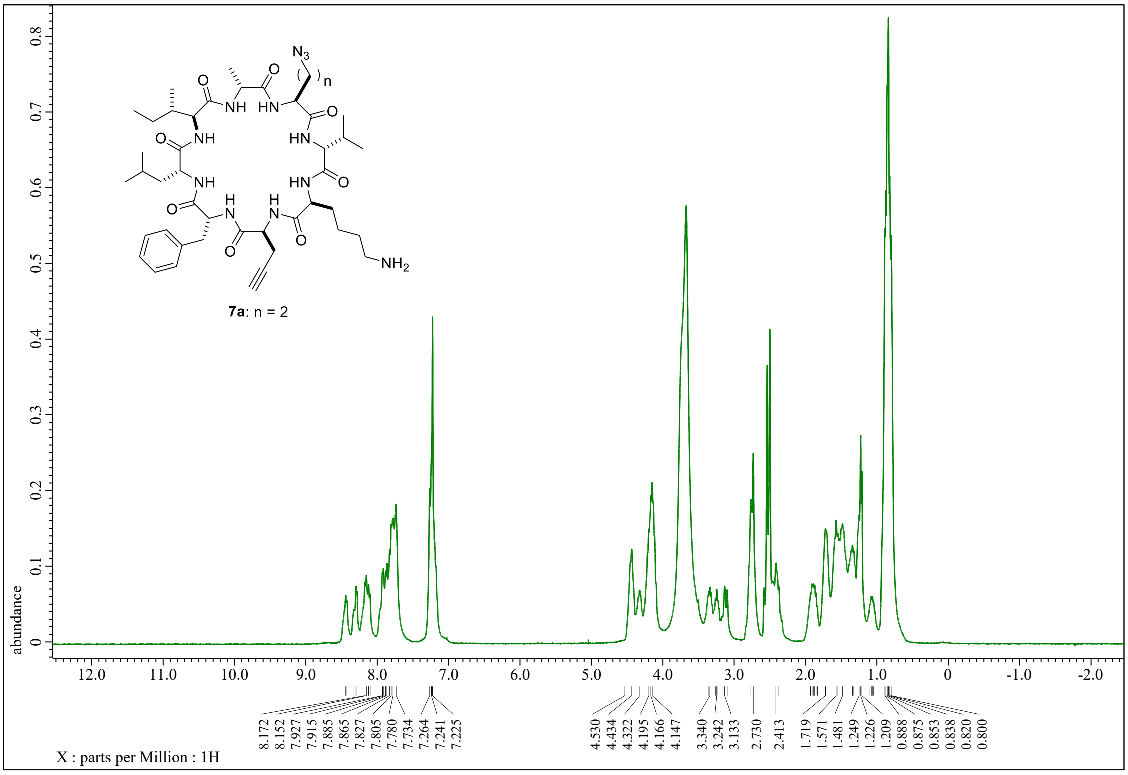

Figure S34. ^{1}H NMR spectrum of **7a** in DMSO-d_6 (400 MHz)

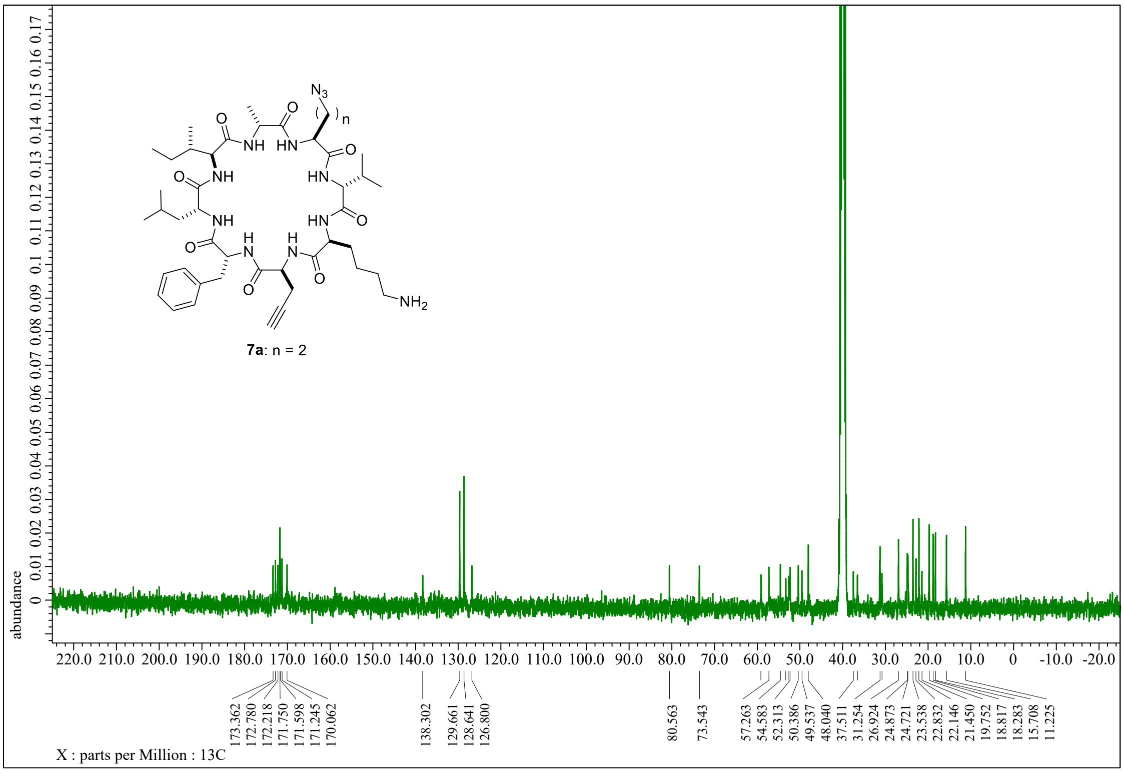

Figure S35. ^{13}C NMR spectrum of **7a** in DMSO-d_6 (100 MHz)

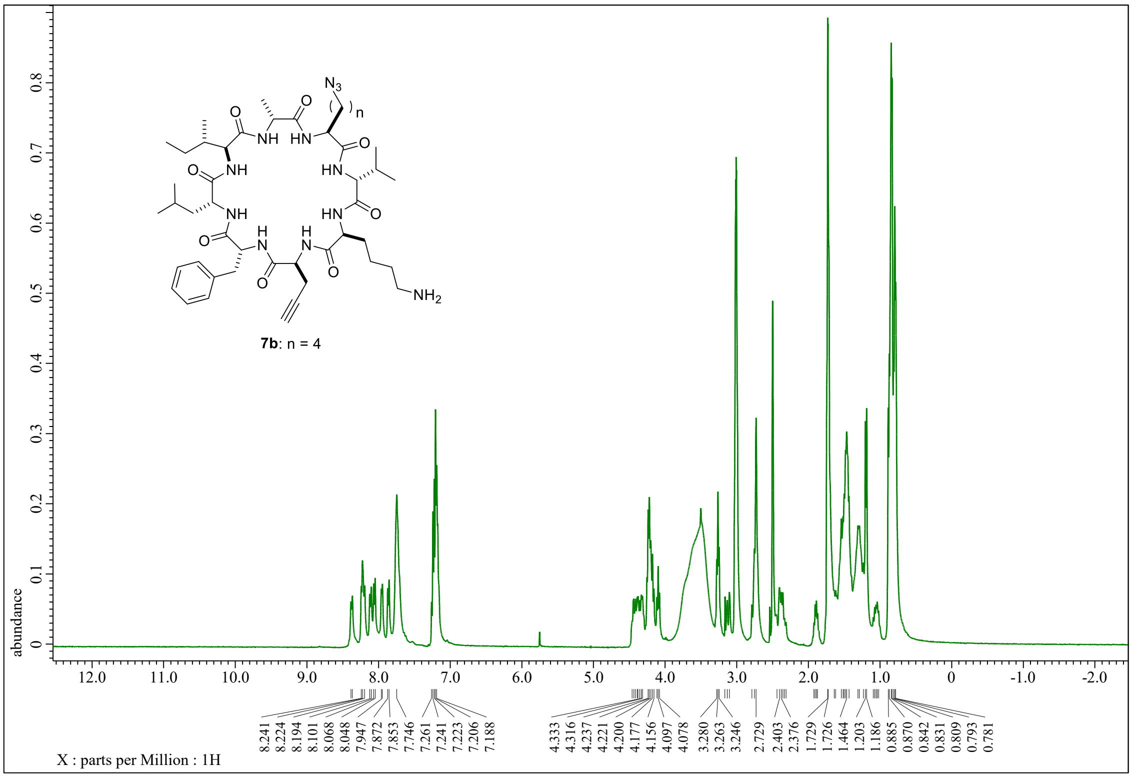

Figure S36. ^{1}H NMR spectrum of **7b** in DMSO-d_6 (400 MHz)

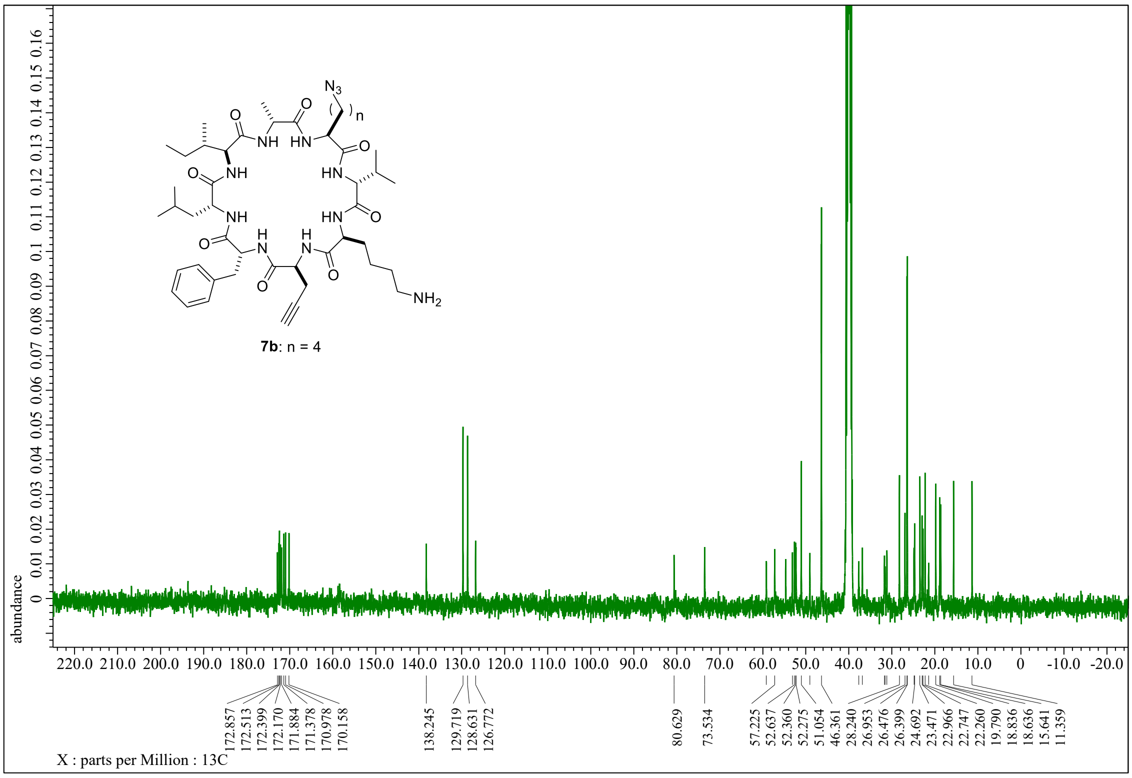

Figure S37. ^{13}C NMR spectrum of **7b** in DMSO-d_6 (100 MHz)

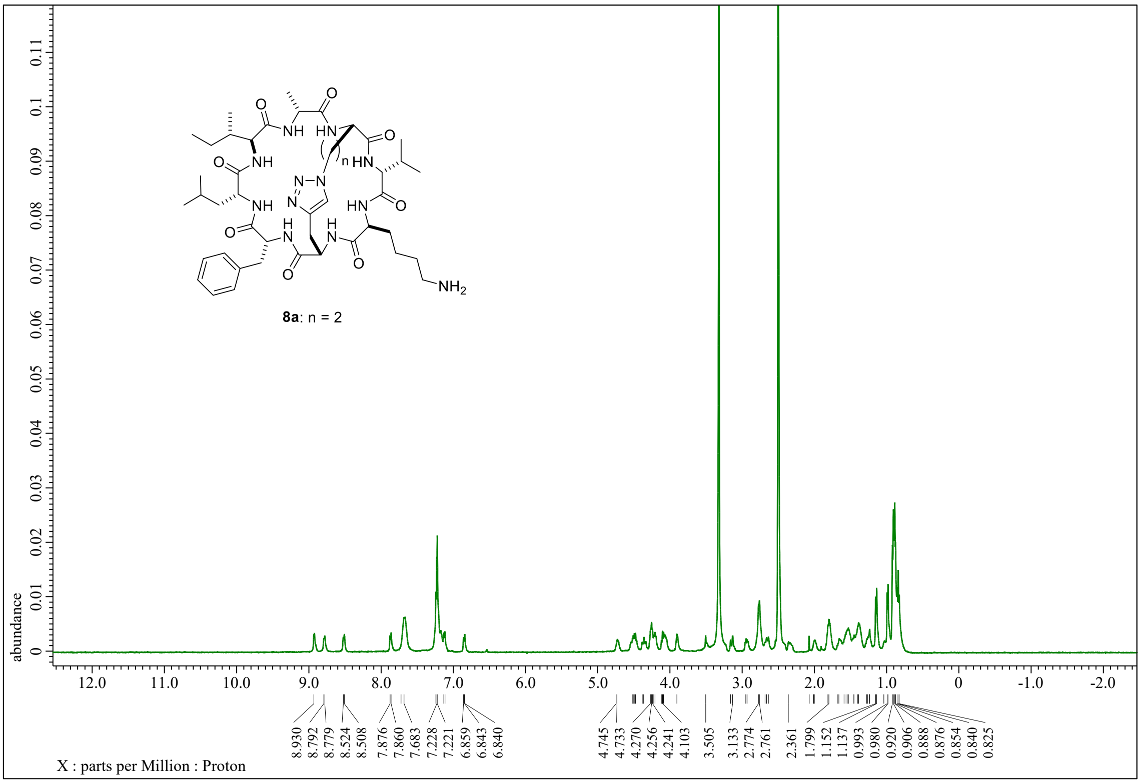

Figure S38. ^{1}H NMR spectrum of **8a** in DMSO-*d*6 (400 MHz)

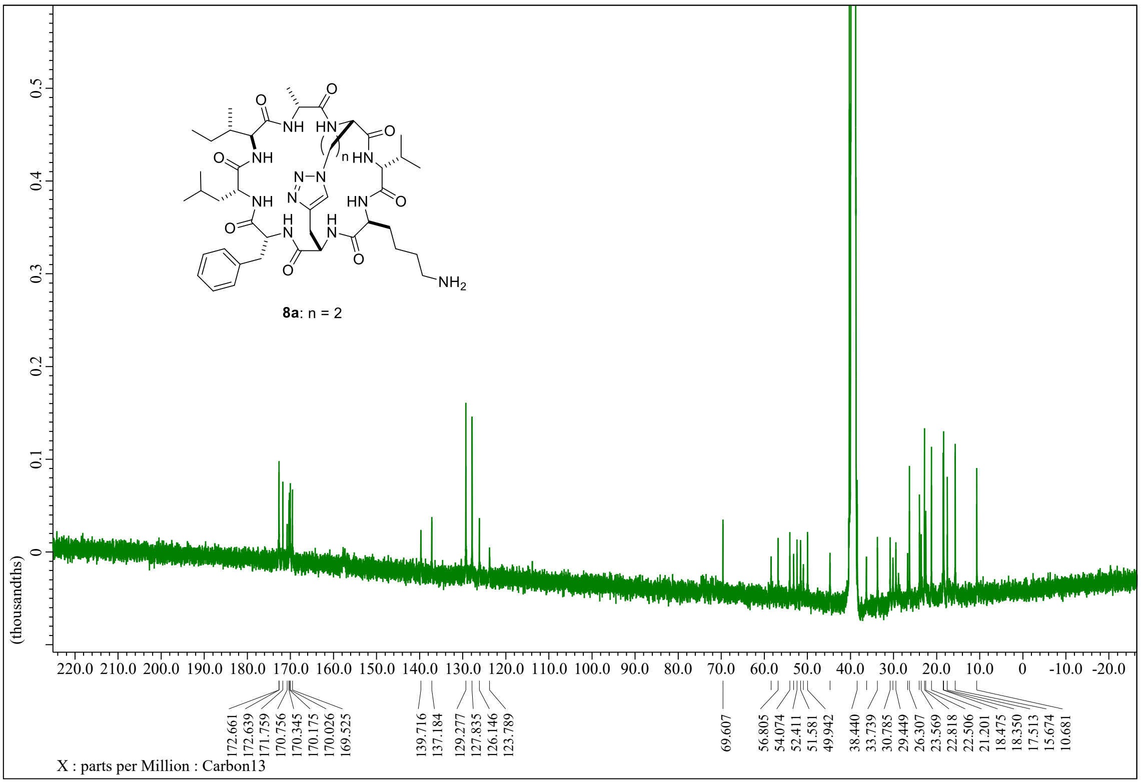

Figure S39. ^{13}C NMR spectrum of **8a** in DMSO-*d*6 (100 MHz)

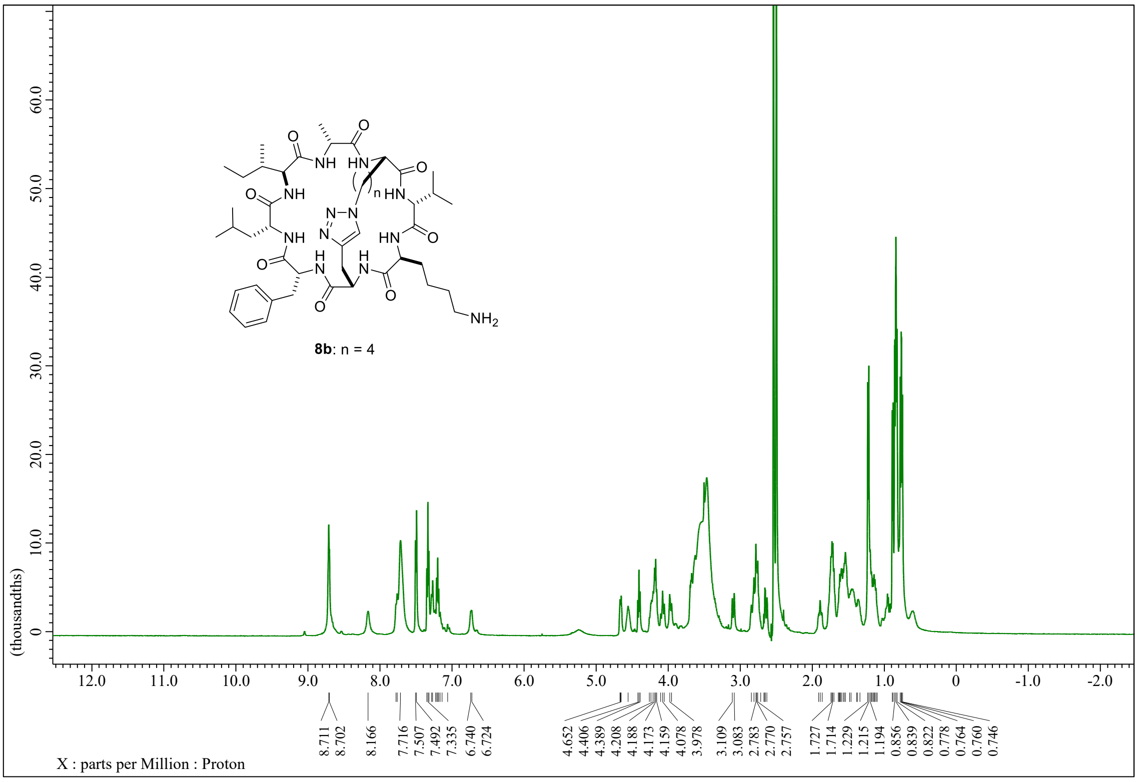

Figure S40. ^{1}H NMR spectrum of **8b** in DMSO-d_6 (400 MHz)

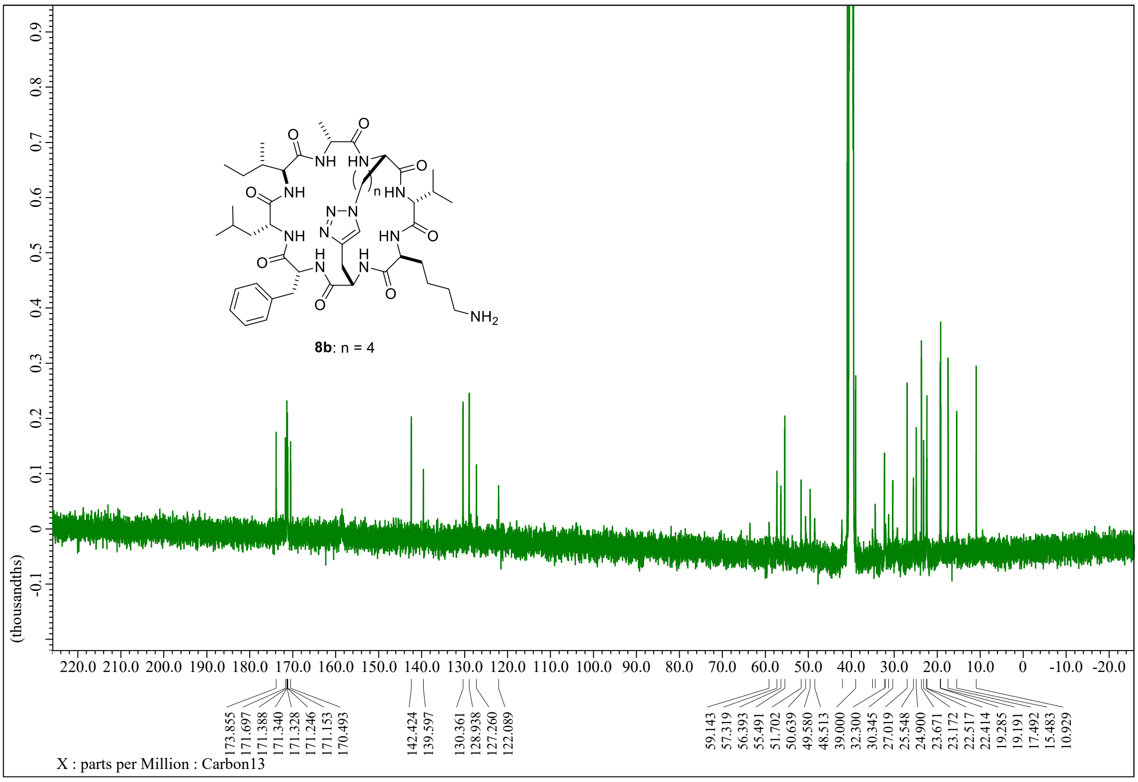

Figure S41. ^{13}C NMR spectrum of **8b** in DMSO-d_6 (100 MHz)

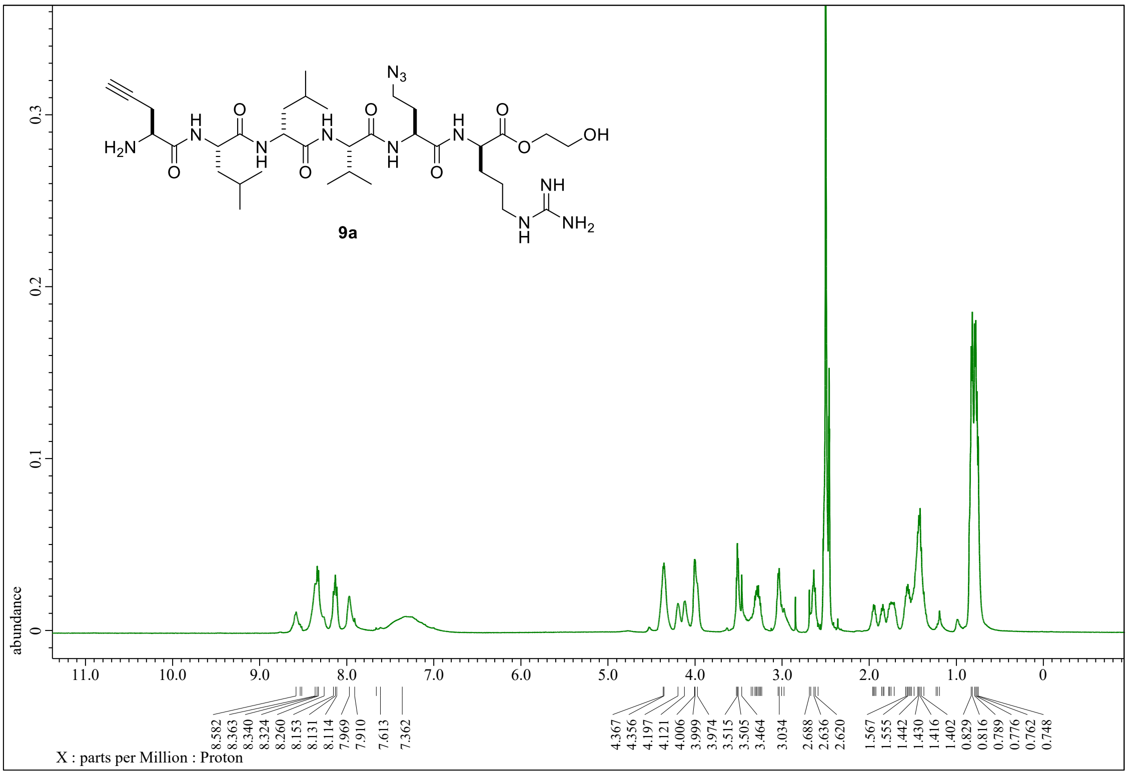

Figure S42. ^{1}H NMR spectrum of **9a** in DMSO-*d*6 (400 MHz)

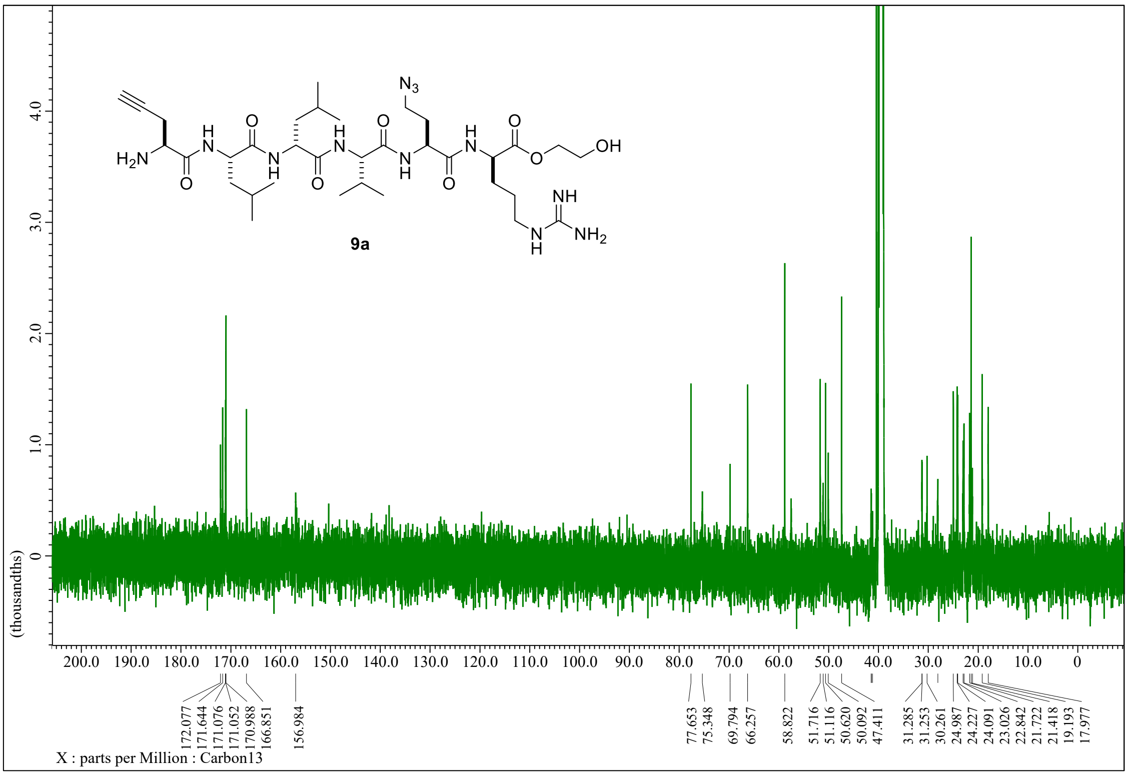

Figure S43. ^{13}C NMR spectrum of **9a** in DMSO-*d*6 (100 MHz)

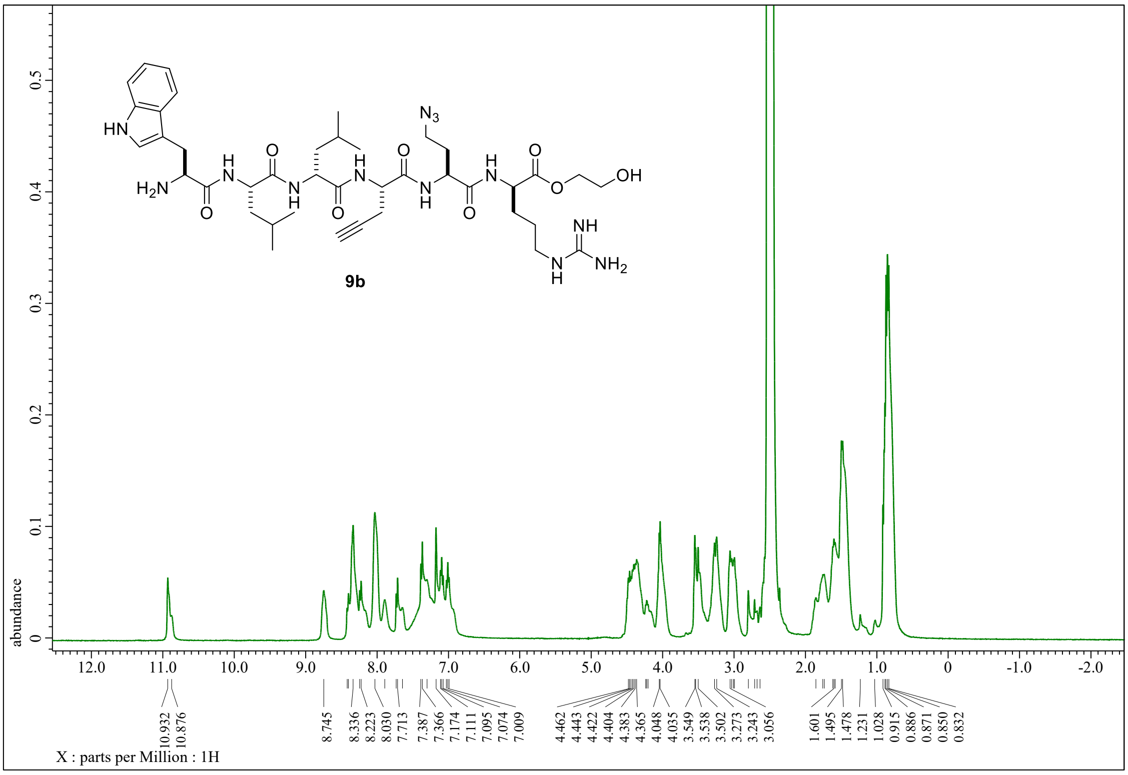

Figure S44. ^{1}H NMR spectrum of **9b** in DMSO-d_6 (400 MHz)

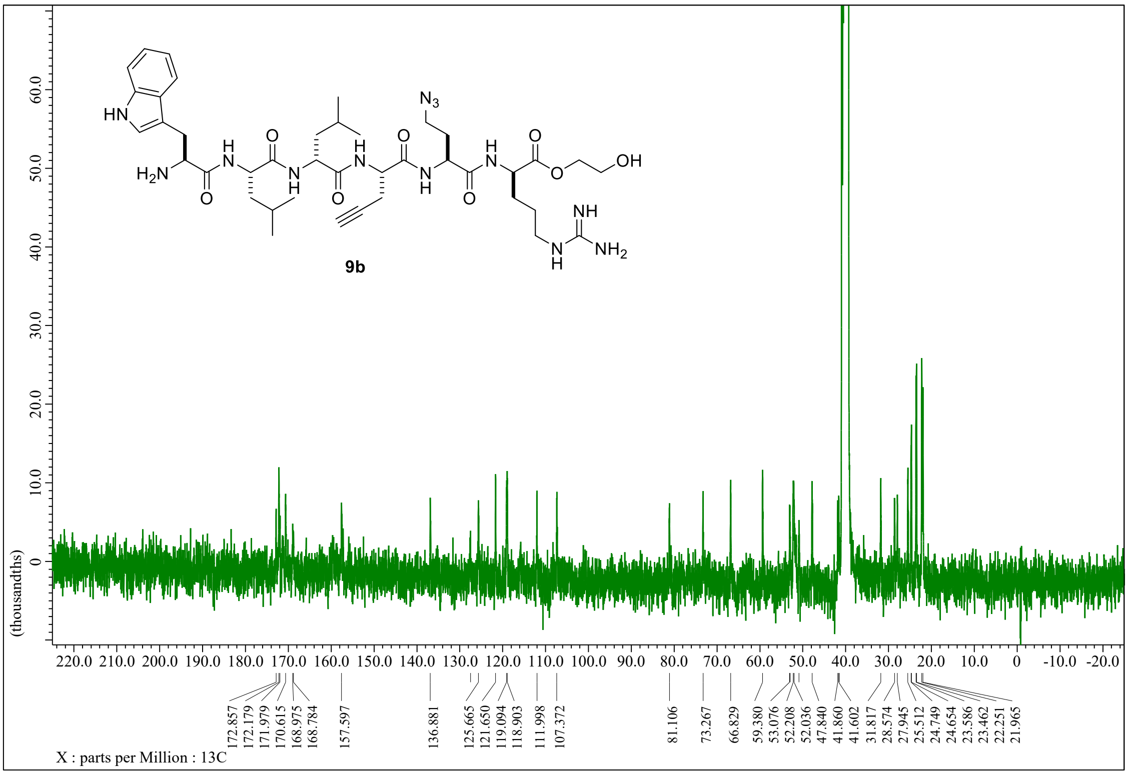

Figure S45. ^{13}C NMR spectrum of **9b** in DMSO-d_6 (100 MHz)

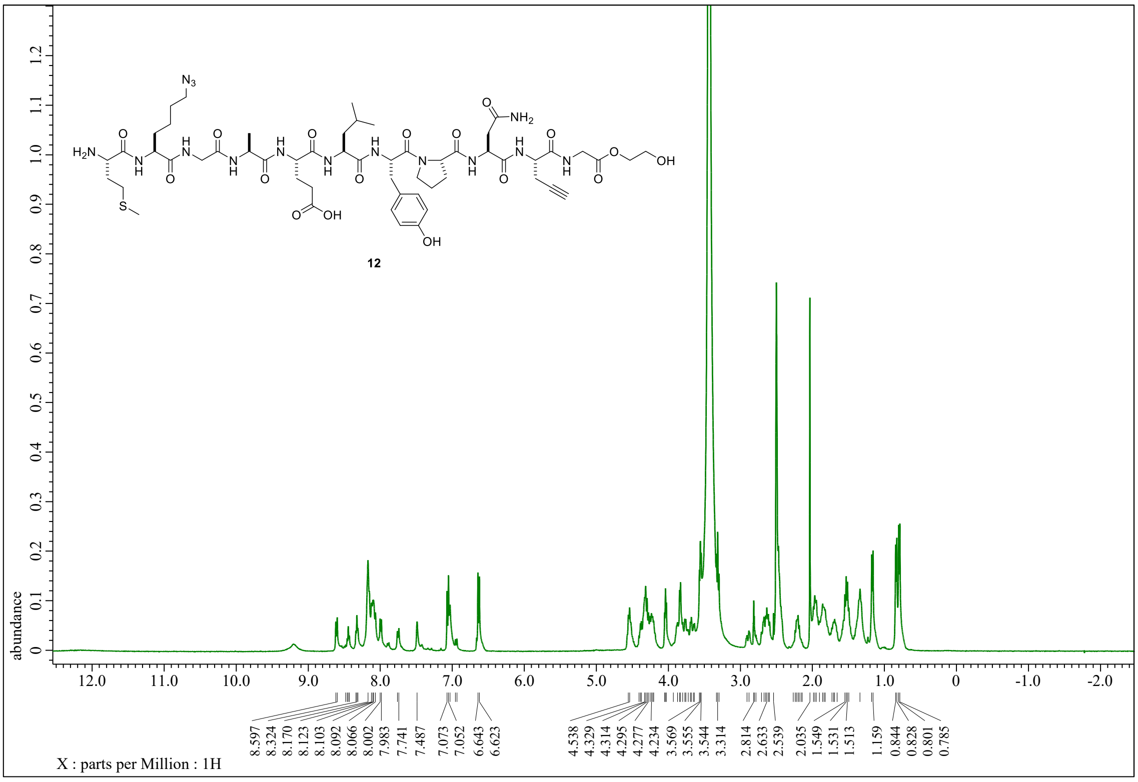

Figure S46. ^{1}H NMR spectrum of **12** in DMSO-d_6 (400 MHz)

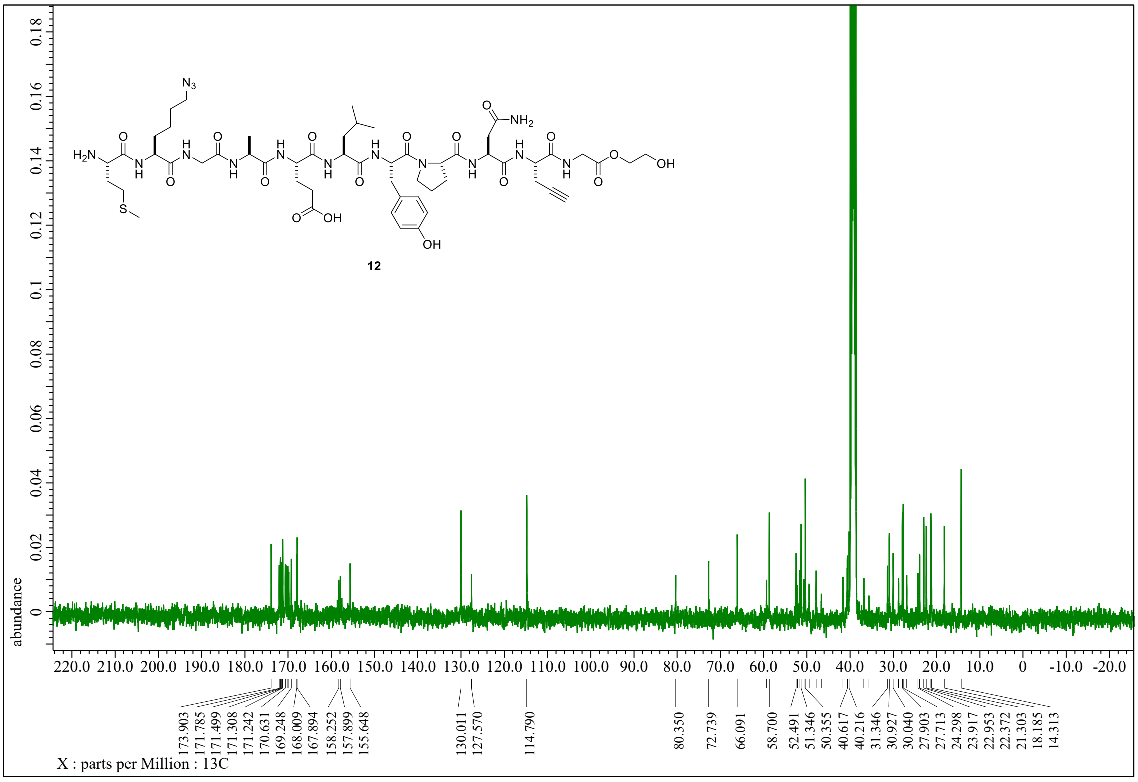

Figure S47. ^{13}C NMR spectrum of **12** in DMSO-d_6 (100 MHz)

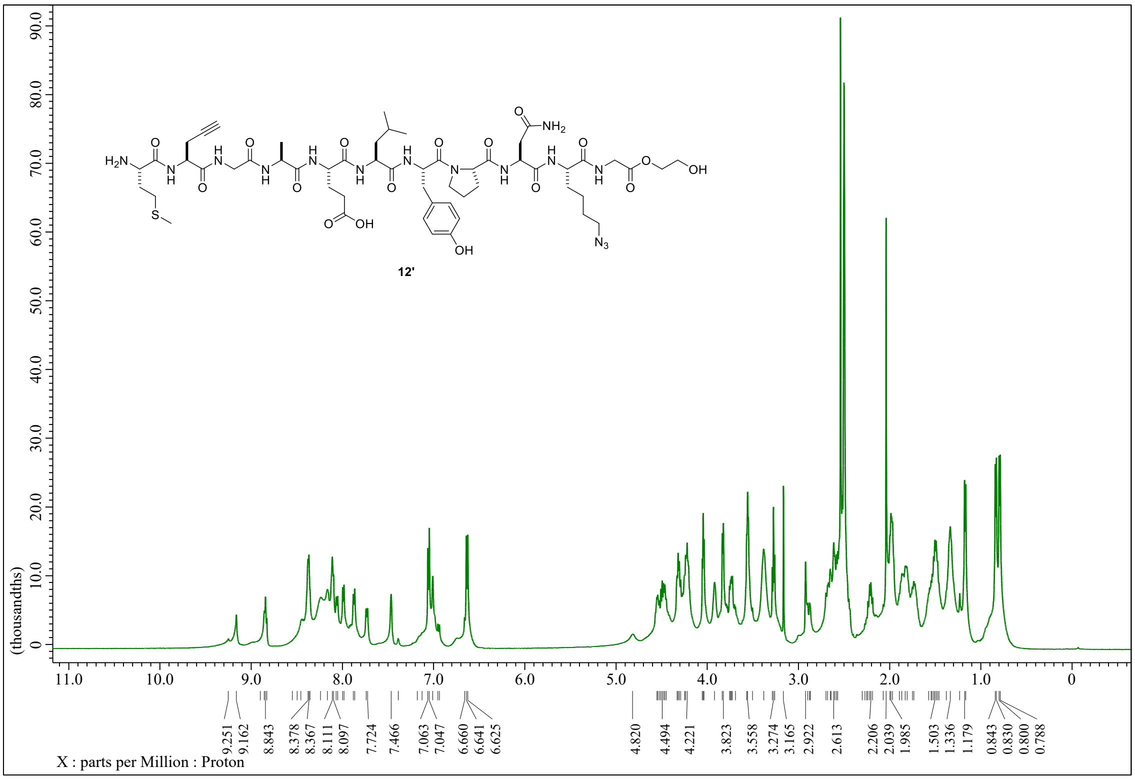

Figure S48. ^{1}H NMR spectrum of **12’** in DMSO-d_6 (500 MHz)

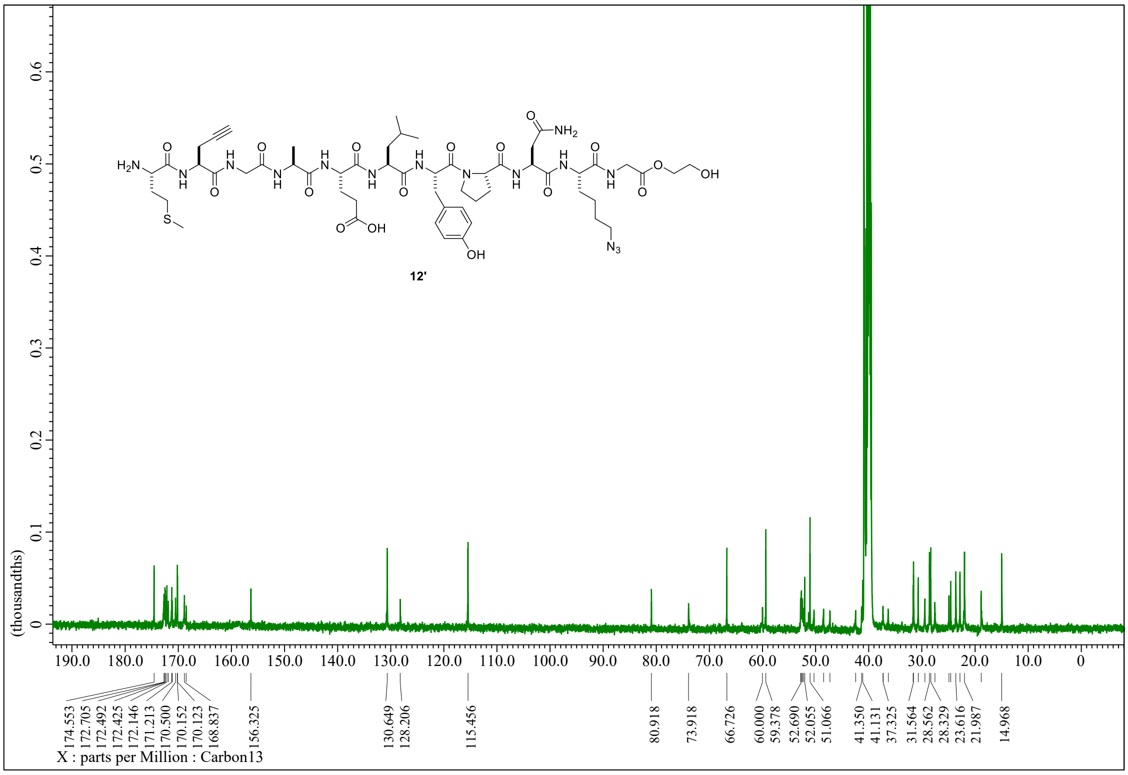

Figure S49. ^{13}C NMR spectrum of **12’** in DMSO-d_6 (125 MHz)
